# Folding Wings like a Cockroach: A Review of Transverse Wing Folding Ensign Wasps (Hymenoptera: Evaniidae: *Afrevania* and *Trissevania*)

**DOI:** 10.1371/journal.pone.0094056

**Published:** 2014-05-02

**Authors:** István Mikó, Robert S. Copeland, James P. Balhoff, Matthew J. Yoder, Andrew R. Deans

**Affiliations:** 1 Frost Entomological Museum, Pennsylvania State University, University Park, Pennsylvania, United States of America; 2 International Centre of Insect Physiology and Ecology, Nairobi, Kenya, and National Museums of Kenya, Nairobi, Kenya; 3 National Evolutionary Synthesis Center, Durham, North Carolina, United States of America; 4 Department of Biology, University of North Carolina, Chapel Hill, North Carolina, United States of America; 5 Illinois Natural History Survey, University of Illinois, Champaign, Illinois, United States of America; CNRS, France

## Abstract

We revise two relatively rare ensign wasp genera, whose species are restricted to Sub-Saharan Africa: *Afrevania* and *Trissevania*. *Afrevania longipetiolata* sp. nov., *Trissevania heatherae* sp. nov., *T. hugoi* sp. nov., *T. mrimaensis* sp. nov. and *T. slideri* sp. nov. are described, males and females of *T. anemotis* and *Afrevania leroyi* are redescribed, and an identification key for Trissevaniini is provided. We argue that *Trissevania mrimaensis* sp. nov. and *T. heatherae* sp. nov. populations are vulnerable, given their limited distributions and threats from mining activities in Kenya. We hypothesize that these taxa together comprise a monophyletic lineage, Trissevaniini, tr. nov., the members of which share the ability to fold their fore wings along two intersecting fold lines. Although wing folding of this type has been described for the hind wing of some insects four-plane wing folding of the fore wing has never been documented. The wing folding mechanism and the pattern of wing folds of Trissevaniini is shared only with some cockroach species (Blattodea). It is an interesting coincidence that all evaniids are predators of cockroach eggs. The major wing fold lines of Trissevaniini likely are not homologous to any known longitudinal anatomical structures on the wings of other Evaniidae. Members of the new tribe share the presence of a coupling mechanism between the fore wing and the mesosoma that is composed of a setal patch on the mesosoma and the retinaculum of the fore wing. While the setal patch is an evolutionary novelty, the retinaculum, which originally evolved to facilitate fore and hind wing coupling in Hymenoptera, exemplifies morphological exaptation. We also refine and clarify the Semantic Phenotype approach used in previous taxonomic revisions and explore the consequences of merging new with existing data. The way that semantic statements are formulated can evolve in parallel, alongside improvements to the ontologies themselves.

## Introduction

Elongate anatomical structures, along which the wing blade is creased, occur in many different forms and serve different functions in Pterygota [1], [2]. Besides the wing veins, which are exclusively convex relative to the dorsal surface of the fore wing in Hymenoptera, two types of resilin-rich longitudinal anatomical structures are present on the fore wing. While the usually concave flexion lines are involved in the control of passive wing deformation during different stages of beat cycle, concave or convex fold lines play important roles in folding the wing to its resting position. The majority of fold lines are axillary folds that are located at the wing base. These folds act as hinges between axillary sclerites and play crucial roles in flexing the neopteran wing over the body. Occasionally other fold lines are present on the wing blade, which are involved in folding mechanisms that decrease the area of the wing blade. While flexion and axillary fold lines are arguably conservative enough to serve as landmarks for characterizing major wing regions across Pterygota, [1], [3], [4], fold lines involved in reducing wing blade area show greater diversity [5], [6]. Four plane wing folding, where four flexible fold lines intersect in one point and hinge the four stiff plates between them, occurs in Coleoptera, Blattodea and Dermaptera,[6].

Hymenoptera exhibit enormous morphological diversity in their wing venation patterns, but the system of flexion and fold lines remains relatively conserved [5], [7]. While wing venation contributes important and informative morphological characters to both higher- and lower-level classification, axillary folds are seemingly uniform throughout the order. So far, only one surface-reducing longitudinal fold has been reported for Hymenoptera [5].

Evaniidae (Hymenoptera) is a monophyletic lineage of cockroach predators, with striking morphological synapomorphies in both the meso- and metasoma that putatively reflect the shape and size of the cockroach ootheca in which they develop [8]–[10]). By contrast, evaniid wings are superficially similar to that of related taxa taxa (i.e., Gasteruptiidae and Aulacidae [11]) and are seemingly unaffected by constraints of host geometry.

Deans and Huben [8] developed numerous fore wing characters for their diagnostic key to evaniid genera. One of these character states was described as “wings often folded apically”, and the illustration of the fore wing of *Afrevania* suggest that the fore wing of some evaniid taxa might be equipped with four-plane wing folding.

We characterize this trait here, including its underlying mechanisms, and discuss the morphological traits of evaniid wing vein geometry and wing base sclerites that possibly lead to the evolution of four-plane wing fold. We examine the possibility that wing folding Evaniidae form a monophyletic group by documenting the morphological and species diversity of a new tribe Trissevaniini. Finally, we compare the wing folding mechanism of Evaniidae with that of other four-plane folding insects.

Balhoff et al. [12] published a revision of the ensign wasp (Hymenoptera: Evaniidae) fauna of New Caledonia, in which they applied a recently developed semantic phenotype (SPs [13]) model to taxonomic descriptions. The application of this model allows a broader array of researchers to search and reuse these data increasing the value of taxonomic work. Balhoff et al. [12] proposed an annotation workflow for SPs generation and discussed limitations and potential benefits such as automated data integration and reasoner-driven queries. Issues related to the longevity and compatibility of SPs from different taxonomic treatments, however, were not challenged, and solutions for this conundrum were not proposed by Balhoff et al. [12]. We refine and clarify the Semantic Phenotype approach here and explore the consequences of merging new with existing data.

## Materials and Methods

Specimens (Table S1) were studied from the following institutions (abbreviations after [14]; curators in parentheses after institutions): NMSA Natal Museum, Pietermaritzburg, Kwa-Zulu Natal, South Africa (M. Mostovski), NMKE National Museum of Kenya, Nairobi, Kenya (M. Gikungu), SAMC Iziko Museum of Cape Town, Cape Town, South Africa (S. van Noort), RMCA Musee Royal de l'Afrique Centrale, Tervuren, Belgium (Elaine de Coninck), PSUC Frost Entomological Museum, Pennsylvania State University, University Park, PA, USA (A. R. Deans).

Glycerin-stored, critical point-dried, and air-dried specimens were used for morphological observations. For the species revision, 93 morphological characters are scored based on observations of critical point-dried and air-dried specimens, using Olympus SZX16 stereomicroscope with Olympus SDF PLAPO 2XPFC objective (230X magnification). Observations on wing base sclerites, wing veins, wing creases and thoracic musculature were carried out on glycerin-stored and critical point-dried specimens, using the same Olympus stereomicroscope. Blue-Tac (Bostik, Inc., Wauwatosa, Wisconsin, USA) was used as a base medium for stabilizing the specimens during dissections made with razor blades, #2 insect pins and #5 forceps.

**Figure 1:**
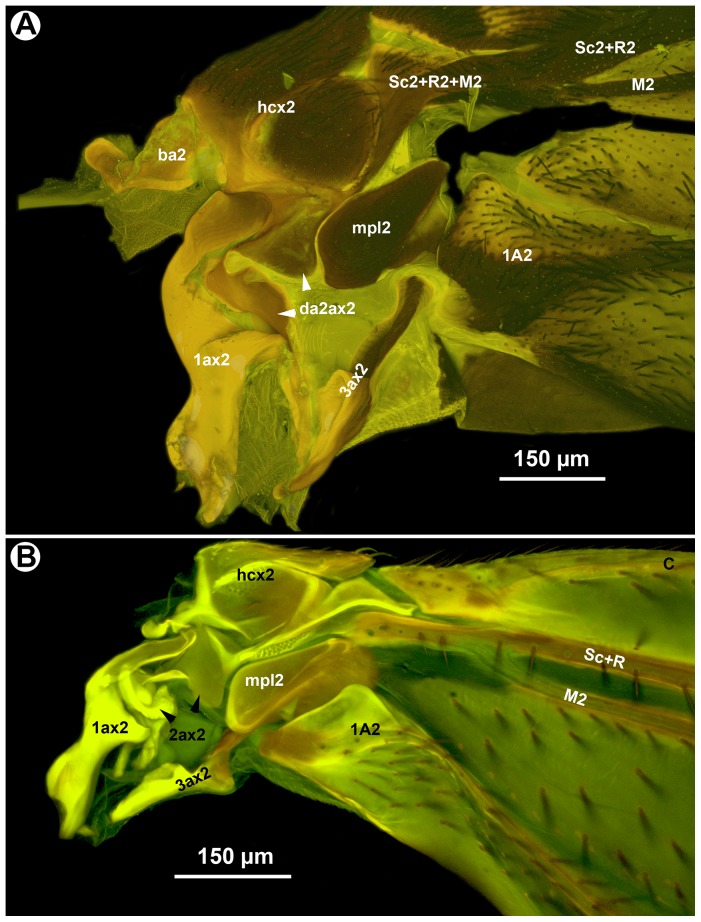
CLSM micrographs of the right wing base of Evaniidae (anterior to the top). A: *Evania albofascialis*. B: *Afrevania longipetiolata* sp. nov.

**Figure 2:**
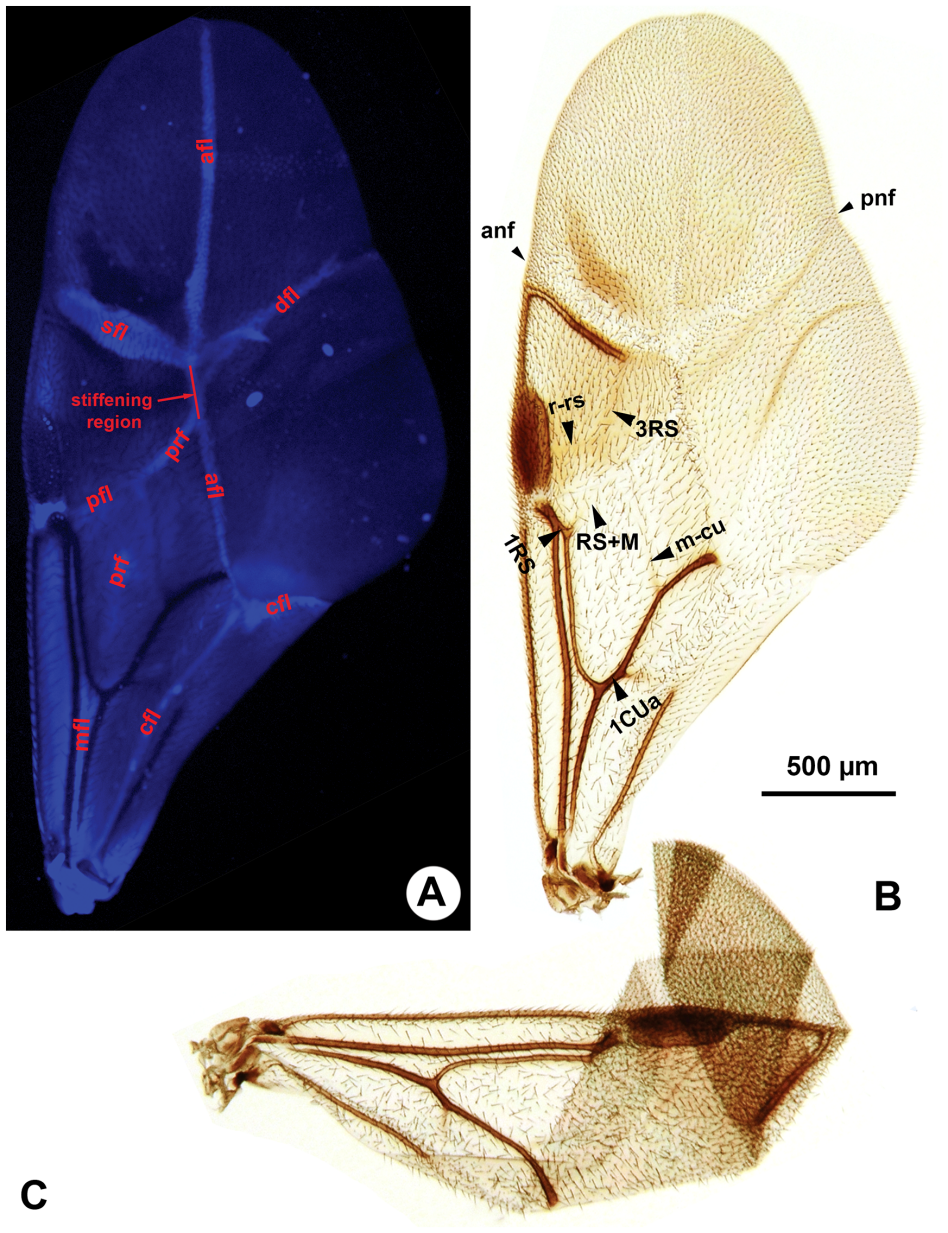
Fore wing of *Afrevania longipetiolata* sp. nov. A: CLSM micrograph of the fully unfolded fore wing, anterior to the left. B: Brightfield image of the fully unfolded fore wing, anterior to the left. C: Brightfield image of the folded fore wing, anterior to the top.

Anatomical structures were visualized with bright field, confocal laser scanning microscopy (CLSM) and scanning electron microscopy (SEM) techniques. Bright field images were taken with an Olympus DP71 digital camera attached to an Olympus ZX41 compound microscope and subsequently processed with CombineZM [15] executing the “do stack” command. CLSM images were taken on glycerin-stored specimens with Zeiss LSM 710 Confocal Microscope. We used an excitation wavelength of 488 and an emission wavelength of 510–680 nm, detected using two channels and visualized separately with two pseudocolors (510–580 nm = green; 580–680 nm = red; Figs 1A, B). To visualizing resilin we used an excitation wavelength of 405 nm and an emission wavelength of 510–680 nm, detected on one channel and visualized with a blue pseudocolor (Fig. 2A). The single SEM image was taken with FEI Nova 400 NanoSEM (Florida State University) on Au-Pd coated specimens. To facilitate understanding of the wing folding mechanism in Trissevaniini, and to demonstrate the rich flexion line system of the tribe relative to that of other Evaniidae, we have created a print-cut-fold model of the fore wing of *Trissevania anemotis* and *Evania albofascialis*. (Figure S1). Images and volume rendered media files were deposited at Figshare (http://dx.doi.org/10.6084/m9.figshare.961802) and at Morphbank (http://www.morphbank.net/?id=835698). Abbreviations of anatomical structures used in Figures are listed in Table S2.

Taxonomic nomenclature, specimen data, supporting images, OTU concepts and natural language (NL) phenotypes were compiled in mx (http://purl.org/NET/mx-database). Taxonomic history, Description, and Material examined sections of taxonomic treatments were rendered from this software. Terminology of morphological statements used in descriptions, identification key and diagnoses are mapped to classes in phenotype-relevant ontologies (Hymenoptera Anatomy Ontology (HAO), Phenotypic Quality Ontology (PATO), Biospatial Ontology (BSPO), Common Anatomy Reference Ontology (CARO); available at http://obofoundry.org/).

NL phenotypes are represented in an EQ format: Entity attribute: value (Table S3). Semantic statements for phenotype descriptions (Table S4) were created in Protégé 4.1 (http://protege.stanford.edu/) using the OWL Manchester syntax (http://www.w3.org/TR/owl2-manchester-syntax/) following Balhoff et al. [12]. Semantic phenotype expression classes about the same phenotype concept from the present and earlier published taxonomic treatments [12], [16] were matched and made “equivalent” (definition following OWL semantics) using Protégé (Table S4). Class equivalences were captured in a separate OWL file, which imported the three taxonomic treatment OWL files of the present and former taxonomic treatments. The full data set, represented in OWL (Web Ontology Language; http://www.w3.org/TR/owl2-overview/ last accessed February 4, 2014), was deposited as two Resource Description Framework (RDF)-XML file (http://www.w3.org/TR/REC-rdf-syntax/ last accessed March 12) in the Dryad repository (http://dx.doi.org/10.5061/dryad.jg909).

The electronic edition of this article conforms to the requirements of the amended International Code of Zoological Nomenclature, and hence the new names contained herein are available under that Code from the electronic edition of this article. This published work and the nomenclatural acts it contains have been registered in ZooBank, the online registration system for the ICZN. The ZooBank LSIDs (Life Science Identifiers) can be resolved and the associated information viewed through any standard web browser by appending the LSID to the prefix “http://zoobank.org/”. The LSID for this publication is: *urn:lsid:zoobank.org:pub:ECF5A640-93F5-4DA6-B210-E404A7BCAAC7.* The electronic edition of this work was published in a journal with an ISSN, and has been archived. It is available from the following digital repositories: PubMed Central and LOCKSS.

## Results

### Semantic representations

In the following addenda we propose an expansion and elaboration of the semantic statement types described by Balhoff et al. [12] and Mullins et al. [16] and briefly discuss NL statements that we were not able express logically.

#### Sculpture

Sculpture is one of the most ambiguous character systems in Hymenoptera morphology descriptions [17]. Not only is the sculpture terminology flooded with synonymy [18], but the semantic representations of sculptural terms also are under almost constant debate and are highly context dependent. Sculptural terms are often used as quality descriptors,

“Axillae reticulate…”

but also as entity terms:

“Mesoscutum with raised and strong reticulation…”

(both statements from [19]).

The homonymic nature of surface sculpture terminology is perhaps the largest obstacle to developing a broadly accepted standard and to placing the concepts in an ontological framework. Here we propose a solution, in which we consider sculpture types as synonyms of the subclasses of the quality descriptor texture:

‘has part’ some (axilla and ‘bearer of’ some reticulate)

and express sculpture-related entity terms as anatomical regions defined by the texture itself:

‘has part’ some (mesoscutum and ‘has part’ (some ‘anatomical region’ and ‘bearer of’ some reticulate’))

Following our reasoning, Hymenoptera-specific sculpture terms can be easily incorporated into the general texture class system of PATO, making Hymenoptera sculptural phenotypes broadly accessible. On the other hand, further quality descriptors can be attached to the post-composed “sculpture defined” anatomical regions making possible the logical representation of statements using “sculpture” as entity terms. This approach makes it possible to handle widely controversial and mostly subjective complex sculptures like “punctate-foveate” (note that “fovea” is a synonym for depression). For the NL statement

“Upper face sculpture: punctuate and foveate”

the logical representation is

‘has part’ some (‘upper face’ and (‘has part’ some (region and (‘bearer of’ some foveate) and (‘has part’ some ((not (depression)) and (‘bearer of’ some punctate)))))).

#### Views

Views are critical when certain qualities are used (e.g., subclasses of distances between anatomical structures and shapes). We have expressed views using BSPO anatomical sides. For the NL statement

“Anterolateral mesopectal projection 2D shape lateral view: square shaped.”

the logical representation is

‘has part’ some (‘anterolateral mesopectal projection’ and ‘has part’ (some ‘lateral side’ and ‘bearer of’ some square)).

#### Length

Distances between anatomical structures play a central role in species diagnoses [20] and embody perhaps the most standardized portion of taxonomic descriptions [21]. Balhoff et al. [12] and Mullins et al. [16] used the PATO class length (http://purl.obolibrary.org/obo/PATO_0000122) in the formal representation of NL phenotypes describing anatomical lines (immaterial anatomical entities) that extends between anatomical structures (material anatomical entities) and can be measured. Although these phenotypes provide information about the nature of morphological characters (i.e. the given phenotypes provide some metric data of an anatomical structure), using the PATO class “length” in these instances result in ambiguous metric data that are neither comparable nor searchable. For instance, in phenotypes concerning “scape length” or “mesoscutum length”, following the logic of PATO “length”, we refer and compare any distance that is defined by any anatomical structures of the “scape” or “anteromesoscutum”.

Ontologies provide the opportunity to define anatomical lines that can be used for refining the formal representation of length characters. Eye height can be explicitly defined as the anatomical line that is the longest of those lines that connect the dorsalmost and ventralmost points of the compound eye (http://purl.obolibrary.org/obo/HAO_0002254). Moreover, we can create a subclass hierarchy of these anatomical lines and post-compose individual lines using these general classes. In the present treatment we use two general anatomical lines, the “median anatomical line” (http://purl.obolibrary.org/obo/HAO_0002272) and the “proximodistal anatomical line” (http://purl.obolibrary.org/obo/HAO_0002273), that can be applied to anatomical structures of the body and the appendages respectively. In the descriptions we refer these concepts as anatomical entities and provide their PATO lengths as numerical entities in the semantic statements. For the NL statement

“Mesoscutellum median length vs. anteromesoscutum median length: mesoscutellum longer than or equals anteromesoscutum”

the logical representation is

‘has part’ some (mesoscutellum and (‘has part’ some (‘dorsal side’ and ((‘has part’ some ‘median anatomical line’) and (‘bearer of’ some (length and (‘is quality measured as’ some ((‘has measurement unit label’ some (length and (‘inheres in’ some (‘median anatomical line’ and ((part_of some ‘dorsal side’) and (part_of some anteromesoscutum)))))) and (‘has measurement value’ some float[> = 1.0f])))))))))

and for the NL statement

“Female scape length vs. compound eye height: eye height is at least 2× as long as scape length”

the logical representation is

‘has_part some (‘eye height’ and (‘bearer of’ some (length and (‘is quality measured as’ some ((‘has measurement unit label’ some (length and (inheres_in some (‘proximodistal anatomical line’ and (part_of some scape))))) and (‘has measurement value’ some float[> = 2.0]))))))).

#### Comparing parts of an anatomical structure with remaining parts of an anatomical structure

Quality differences between a part of an anatomical structure and the remaining parts are often provided in taxonomic descriptions especially when color phenotypes are given. In the statement

“Mandible color: black with dark brown mandibular teeth.”

The coloration of the mandibular teeth is compared with the rest of the mandible. We expressed this statement logically in Balhoff et al. [12] as

(has_part some (mandible and (has_part some (‘proximal region’ and (‘bearer of’ some black))))) and (has_part some (tooth and (‘bearer of’ some ‘dark brown’)))

where the proximal region of the mandible was vaguely defined and certainly did not refer to the mandible exclusive of the teeth. In the present treatment we have corrected this ambiguity and expressed the phenotype as

(‘has part’ some ((not (tooth) and (part_of some (mandible and (bearer_of some black))))) and (has_part some (tooth and (bearer_of some ‘dark brown’))).

### Taxonomic treatment

**Trissevaniini tr. nov.**

#### Diagnosis

**Figure 3:**
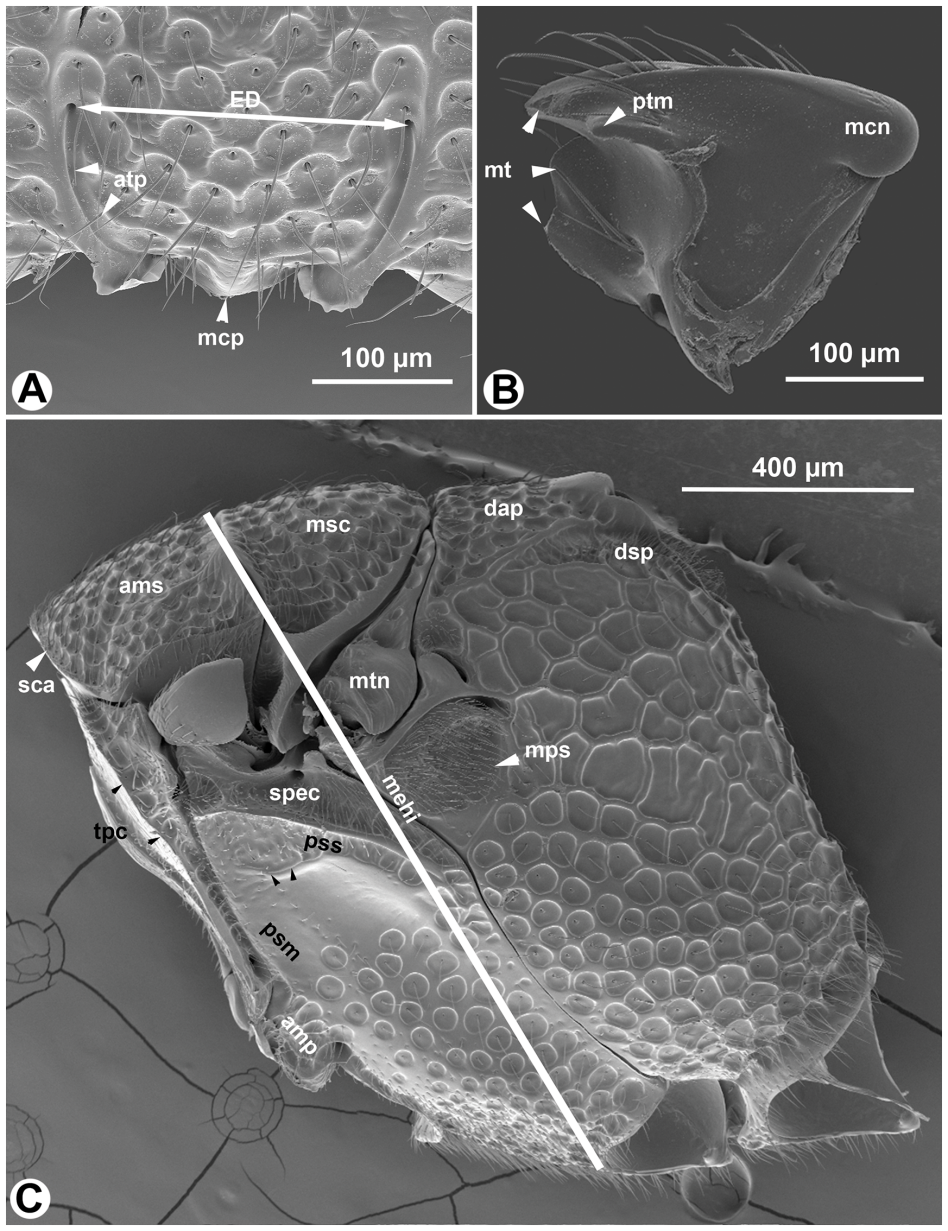
SEM micrographs of *Afrevania longipetiolata* sp. nov. A: Anteroventral region of the face, anterior view, dorsal to the top. B: mandible, posterior view, distal to the left. C: Mesosoma, lateral view, anterior to the left.

**Figure 4:**
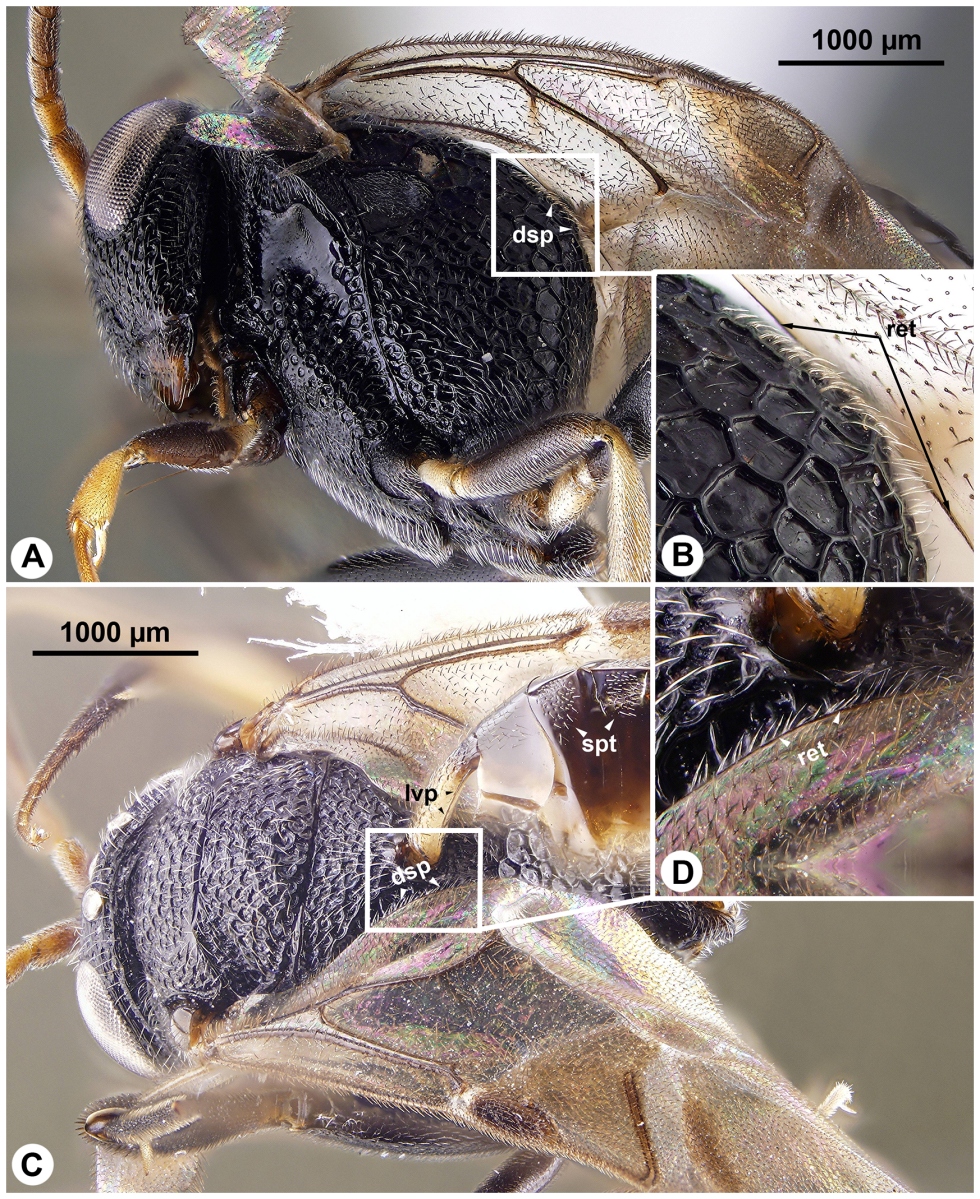
Brigthfield images of *Afrevania longipetiolata*, anterior to the left. A: Head and mesosoma, lateral view. B: Dorsal region of the metapectal-propodeal complex, lateral view. C: Habitus, posterodorsal view. D: Posterolateral region of the metapectal-propodeal complex, posterodorsal view.

**Figure 5:**
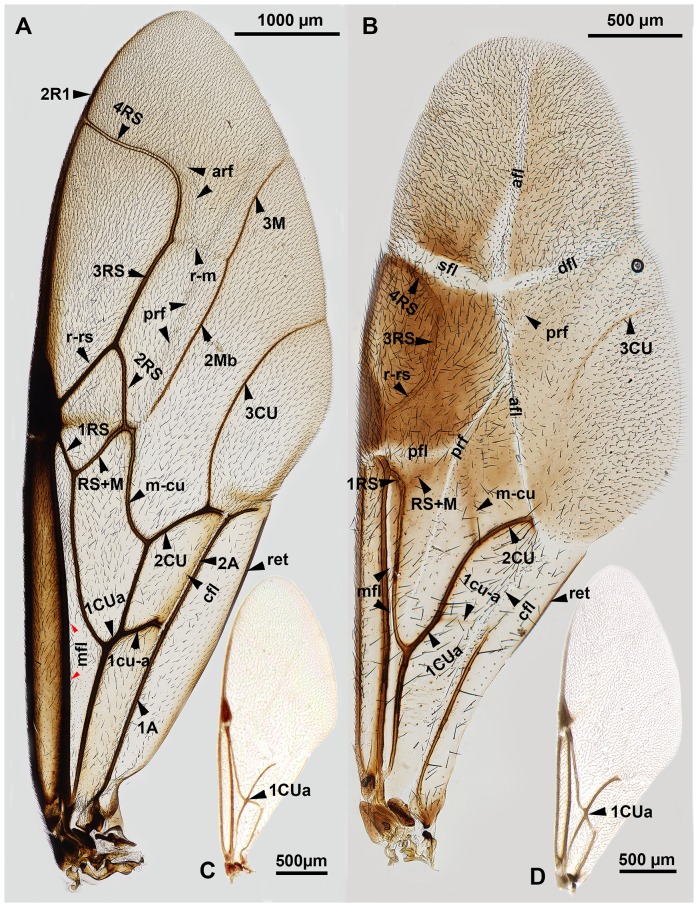
Brightfield images of the fore wing of Evaniidae, anterior to the left. A: *Evania albofascialis*. B: *Trissevania anemotis*. C: *Rothevania valdiviana*. D: *Brachygaster minutus*.

Mesoscutellum posterior margin vs. metapectal-propodeal complex anterior margin medially: Posterior margin of mesoscutellum adjacent medially to anterior margin of metapectal-propodeal complex (msc, dap: Fig. 3C). Metanoto-metapectal-propodeal complex conjunctiva count: absent (metanotum and metapectal-propodeal complex fused). Dorsolateral setal patch of the metapectal-propodeal complex count: present (dsp: Figs 3C, 4A, C). Anterodistal notch of the fore wing count: present (anf: Fig. 2B). Posterodistal notch of the fore wing count: present (pnf: 2B). Prestigmal fold line count: present (pfl: Figs 2A, 5B). Discal fold line count: present (dfl: Figs 2A, 5B). Anal-marginal fold line count: present (afl: Figs 2A, 5B). Poststigmal fold line count: present (sfl: Figs 2A, 5B).

#### Description

Mandibular teeth count: 3. Posterior tooth of the mandible count: present. Epistomal distance vs. clypeo-compound eye distance: epistomal distance shorter than clypeo-compound eye distance. Anterior tentorial pit count: present. Anterior tentorial pit shape: elongate. Antennal shelf count: absent. Preorbital carina count: absent. Subantennal groove count: present. Subantennal carina count: absent. Upper face sculpture: foveate. Vertex sculpture: foveate. Carinae on gena parallel with posterior margin of compound eye count: present. Female flagellum ventral sensillar patch spatial arrangement: F6–F11. Female apical flagellomere shape: about 2× as long as wide. Female scape length vs. compound eye height: eye height is at least 2× as long as scape length. Pronotal lobe carina count: absent. Cranial scrobe of anteromesoscutum count: present. Scrobal carina of the anteromesoscutum: present. Anteromesoscutum sculpture: foveate. Distance between depressions vs. diameter of depressions on foveate region of anteromesoscutum: less than the diameter of one depression. Areas in between depressions on foveate region of anteromesoscutum sculpture: crenulate. Depression diameter on foveate region of median area of the anteromesoscutum vs. depression diameter on foveate region of lateral area of the anteromesoscutum: depressions larger on median area of the anteromesocutum than on lateral area of the anteromesosocutum. Notaulus count: present. Notaulus shape: falciform. Mesoscutal humeral sulcus anterior end vs. preaxilla anterior end: mesoscutal humeral sulcus anterior end is adjacent to dorsal margin of preaxilla anterior end. Parapsidal signum count: present. Scutoscutellar suture sculpture: foveate. Mesoscutellum posterior margin vs. metapectal-propodeal complex anterior margin medially: Posterior margin of mesoscutellum is adjacent medially to anterior margin of metapectal-propodeal complex. Metanoto-metapectal-propodeal complex conjunctiva count: absent (metanotum and metapectal-propodeal complex fused). Ventro-lateral region of mesosoma texture: foveate. Anterolateral mesopectal projection count: present. Anterolateral mesopectal projection 2-d shape: square shape. Profemoral scrobe of the mesopectus count: present. Speculum count: present. Carina delimiting ventrally anterior region of prespecular sulcus count: present. Ventral margin of mesopectus length: longer than ventral margin of metapectus length. Metapleural sulcus position: horizontal. Transmetapectal line count: absent. Dorsolateral setal patch of the metapectal-propodeal complex count: present. Dorsal area of the metapectal-propodeal complex sculpture: foveate. Gastral scrobe count: present. Submedian propodeal projection count: absent. Nucha count: absent. Fore wing distal margin in flexed position vs. metasoma distal margin: extending beyond posterior margin of metasoma. Anterodistal notch of the fore wing count: present. Posterodistal notch of the fore wing count: present. Prestigmal flexion line count: present. Discal fold line count: present. Anal-marginal fold line count: present. Poststigmal fold line count: present. Fore wing m-cu structure: not tubular, marked by dark line. Fore wing 1CUa length vs. width: more than 2× as long as wide. Fore wing 1CUa orientation: oriented posterodistally. Fore wing 1cu-a structure: not tubular, marked by darker line. Fore wing 2R1 count: absent. Fore wing 2A count: absent. Fore wing 1RS count: absent. Fore wing RS+M structure: not tubular, marked by dark line. Fore wing 2RS count: absent. Fore wing 3CU distal region count: absent. Fore wing 3M count: absent. Female metatibial spines count: present. Metatibia length vs. metabasitarsus length: metatibia 1.4× to 1.6× as long as metabasitarsus. Petiole texture: foveolate. Petiole pilosity: dense. Lateroventral carina of the petiole count: present. Setiferous patch on dorsal region of abdominal terga 4–7 in female count: present.

#### Comments

Due to the limited number of specimens available for *Afrevania leroyi* and *Trissevania mrimaensis* we were not able to perform dissections and repositioning of these species. Only one female represents *Trissevania mrimaensis*. Given this situation we were not able to score some otherwise invariable characters, and thus they were not involved in the description of the new tribe. The phenotypes shared by all scored Trissevaniini taxa are: Median clypeal projection count: present; Median clypeal projection sharpness: blunt; Median conjunctiva of abdominal tergum 9 presence: present.

#### Key of world species of Trissevaniini

**Figure 6:**
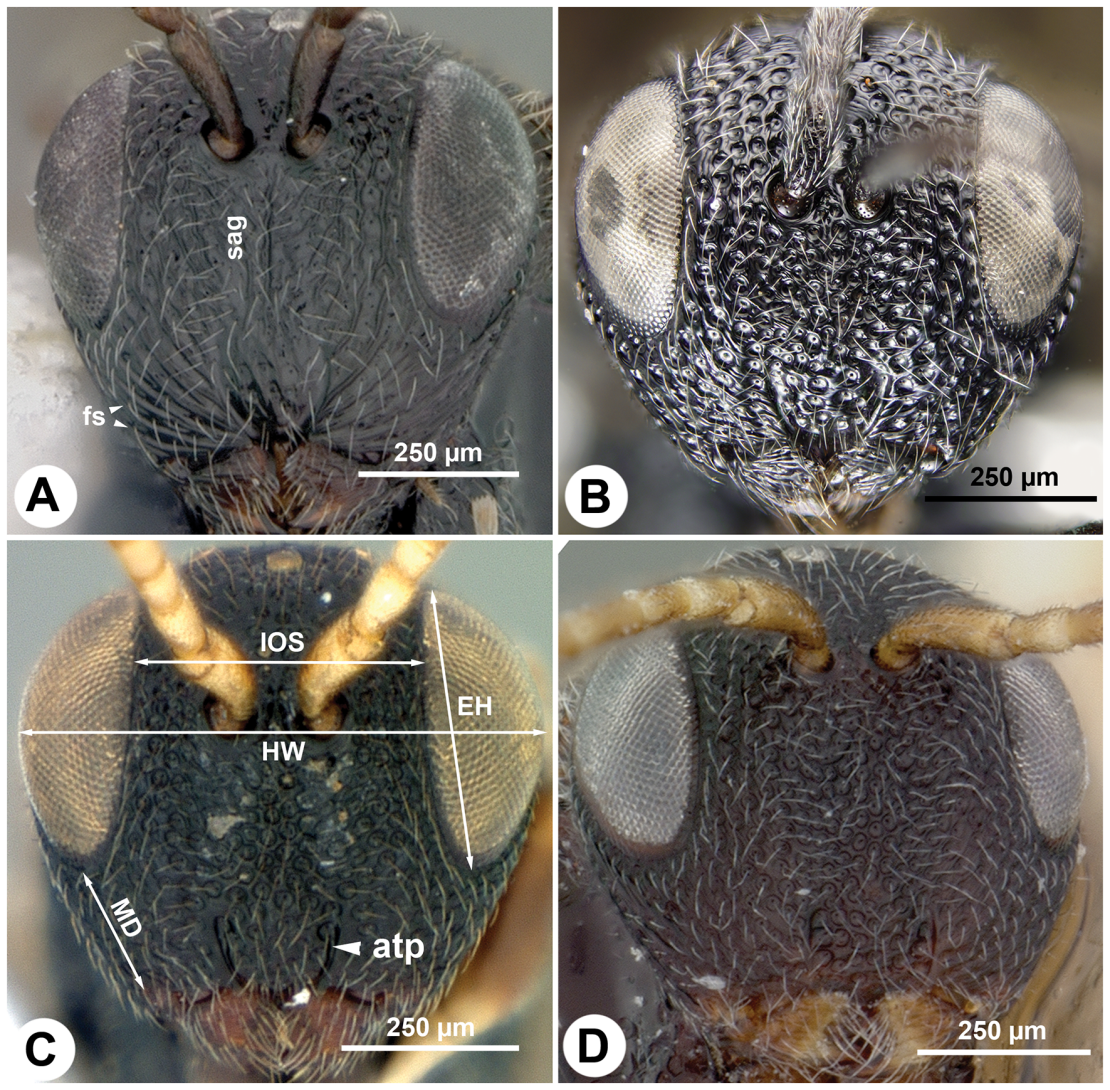
Brightfield images of the head of Trissevaniini, anterior view, dorsal to the top. A: *Trissevania anemotis* Kieffer, 1913. B: *Trissevania slideri* sp. nov. C: *Afrevania longipetiolata* sp. nov. D: *Afrevania leroyi* Benoit, 1953.

**Figure 7:**
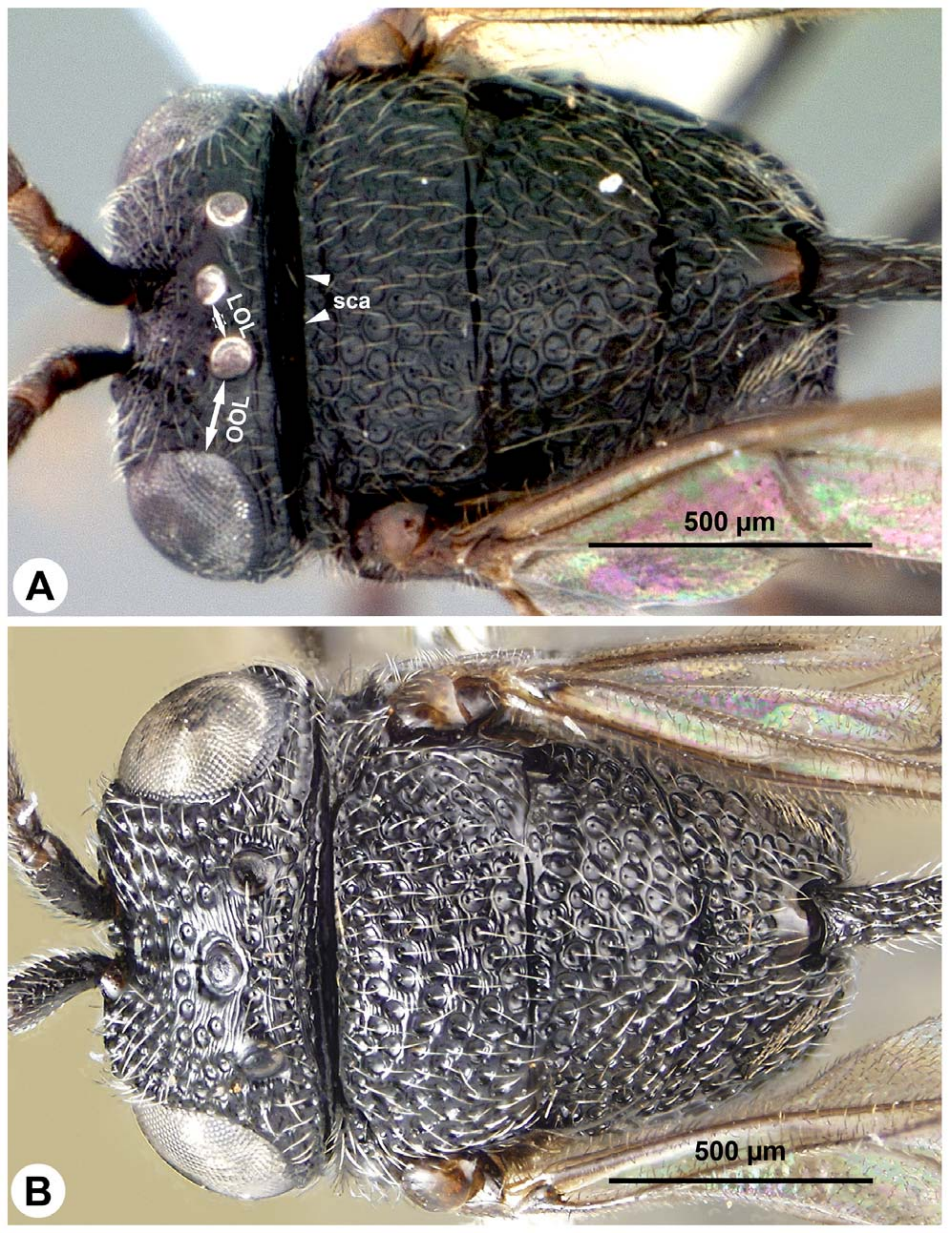
Brightfield images of the head and the mesosoma of *Trissevania* species, dorsal view, anterior to the left. A: *Trissevania anemotis* Kieffer 1913. B: *Trissevania slideri* sp. nov.

**Figure 8:**
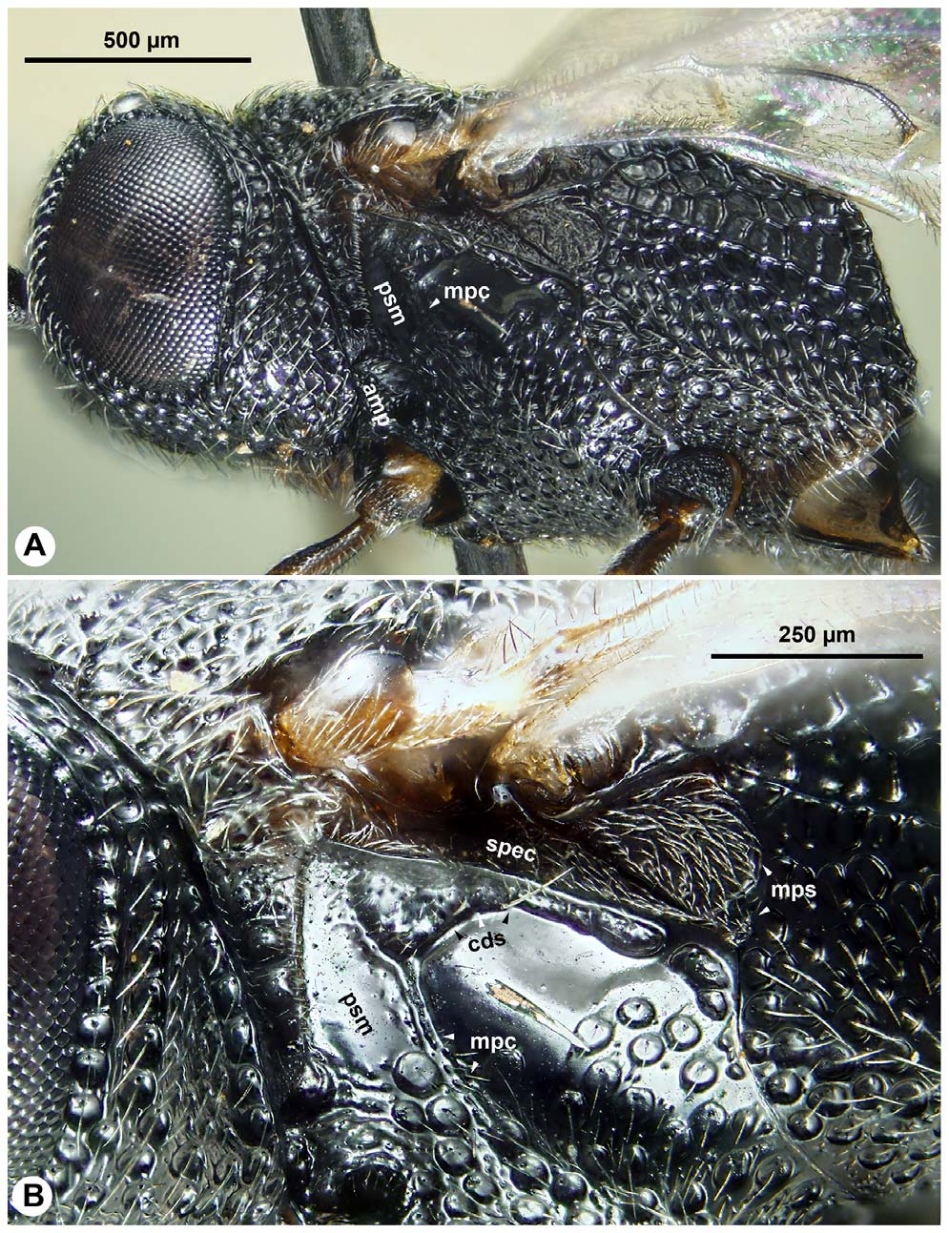
Brightfield images of *Trissevania mrimaensis* sp. nov., lateral view, anterior to the left. A: Head and mesosoma. B: dorsolateral region of the mesothorax.

**Figure 9:**
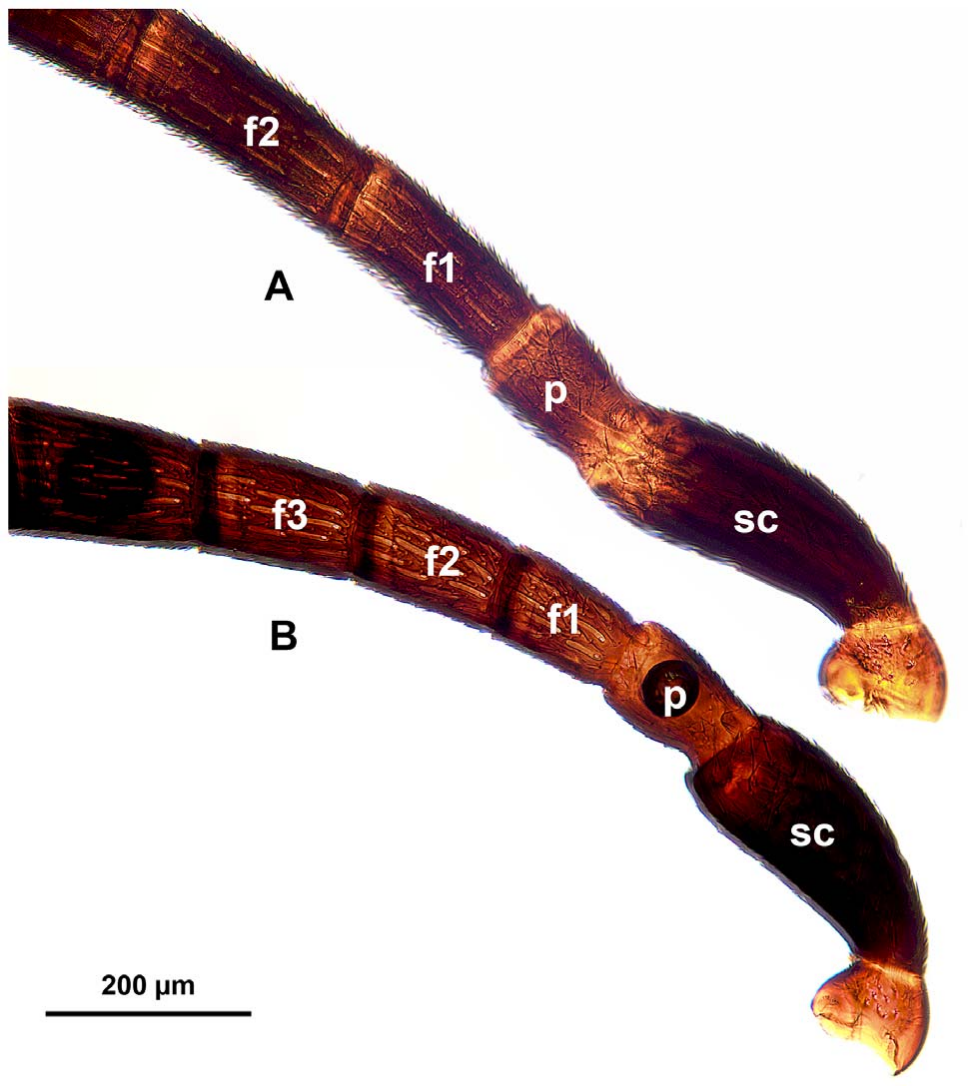
Brightfield images showing the proximal part of the male antenna of *Trissevania* species, distal to the left. A: *Trissevania slideri* sp. nov. B: *Trissevania hugoi* sp. nov.

**Figure 10:**
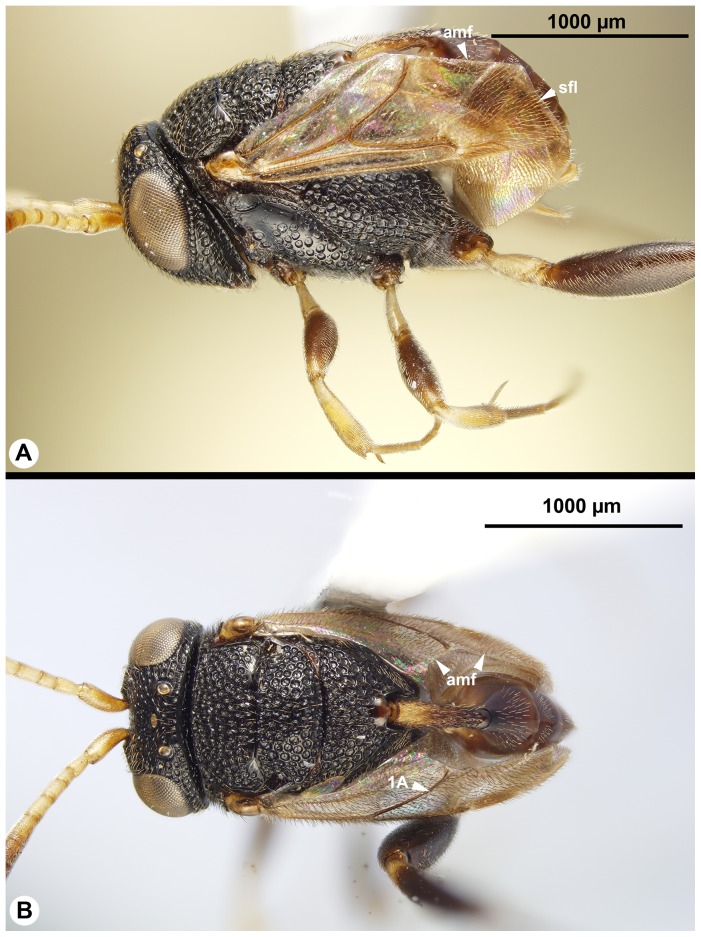
Brightfield images of *Afrevania longipetiolata*, anterior to the left. A: Dorsolateral view. B: Dorsal view.

**Figure 11:**
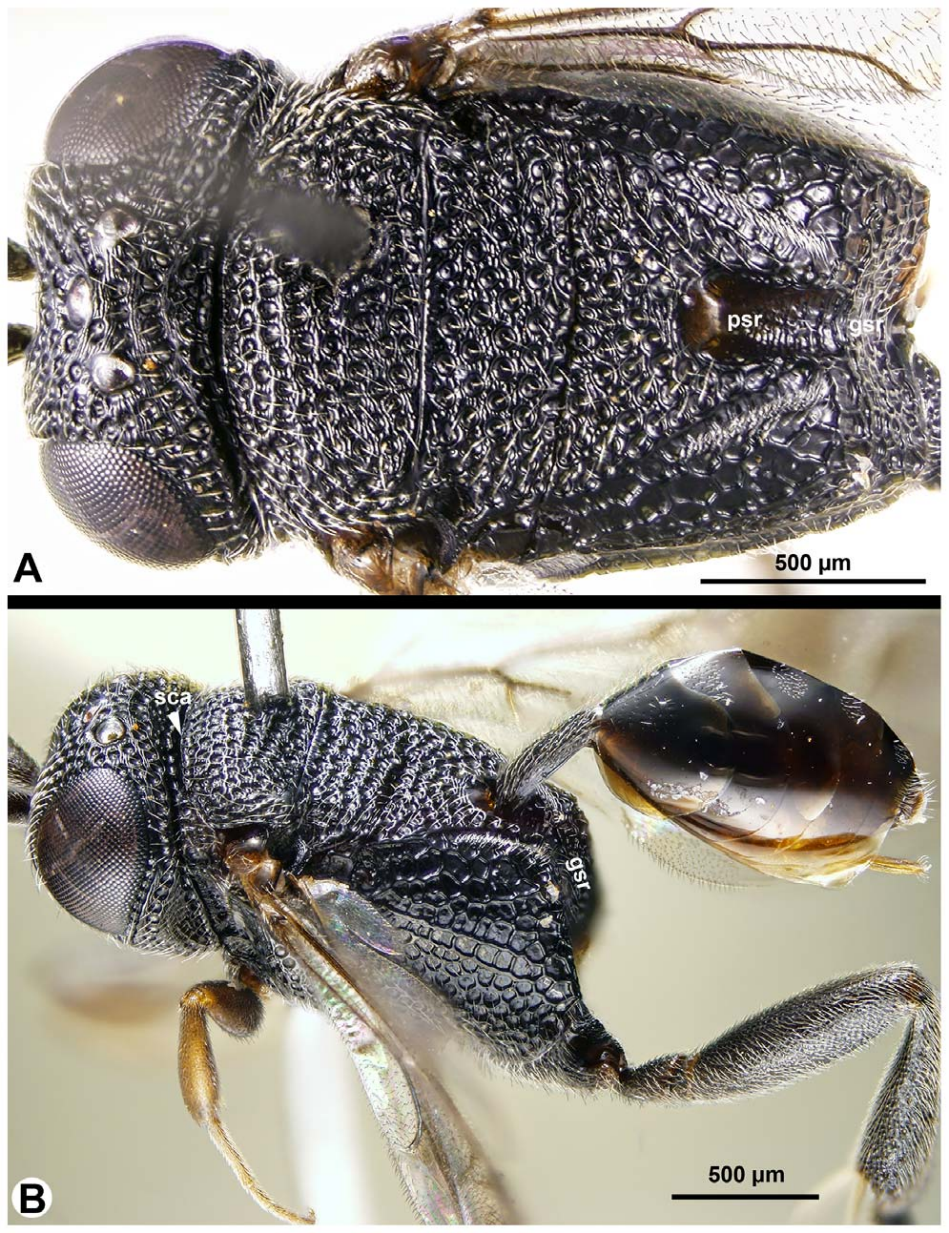
Brightfield images of *Trissevania mrimaensis* sp. nov., anterior to the left. A: Head and mesosoma, dorsal view. B: Habitus, dorsolateral view.

**Figure 12:**
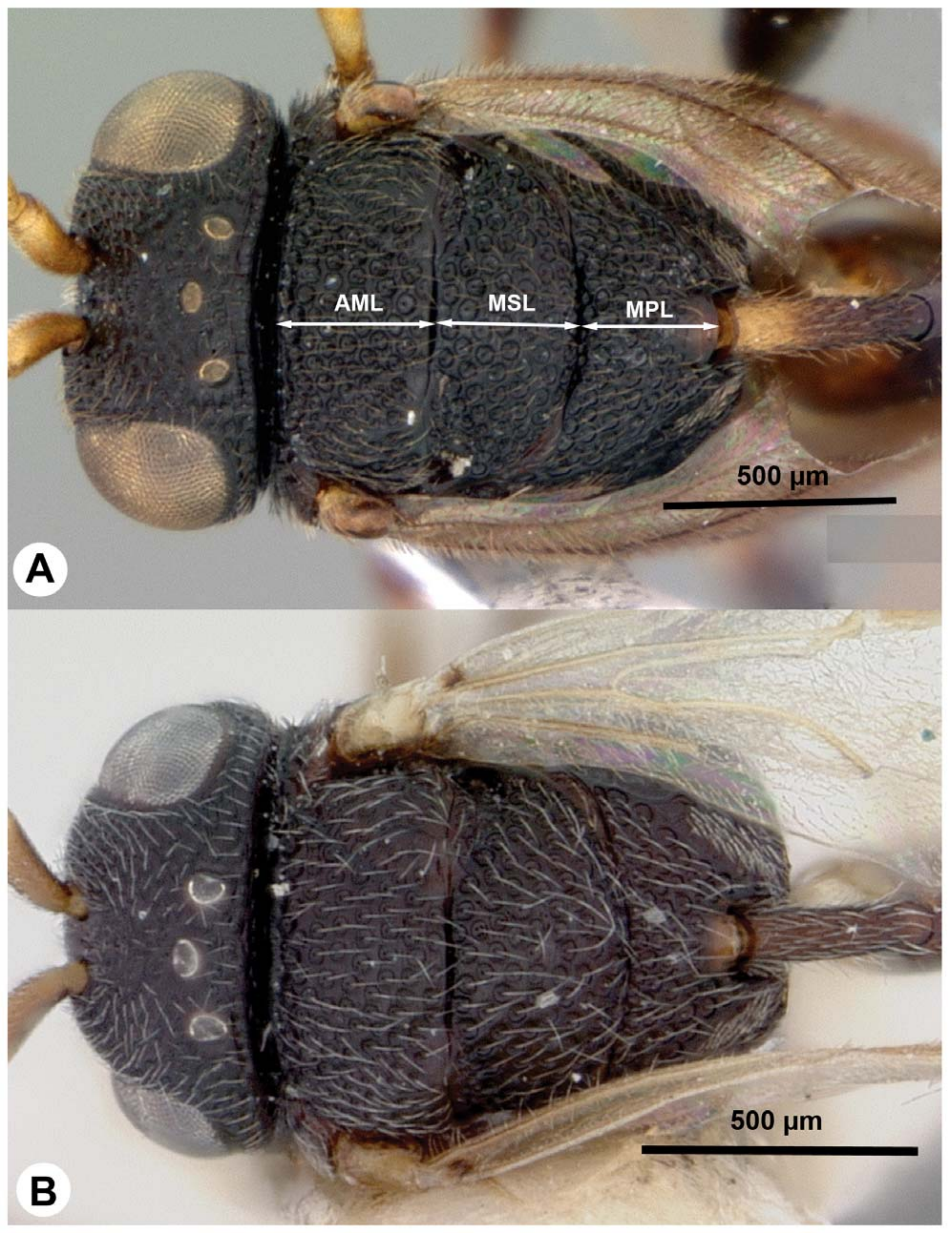
Brightfield images of the head and the mesosoma of *Afrevania* species, dorsal view, anterior to the left. A: *Afrevania longipetiolata* sp. nov. B: *Afrevania leroyi* Benoit 1953.

1. Fore wing vein 3RS structure: tubular (Fig 5B); Fore wing vein r-rs structure: tubular (Fig 5B)…**2** (*Trissevania*)**-** Fore wing vein 3RS structure: not tubular, marked by dark line (Figs 2A–C); Fore wing vein r-rs structure: not tubular, marked by dark line (Figs 2A–C)…**6** (*Afrevania*)
2. Facial striae presence: present (fs: Fig. 6A); Malar distance vs. eye height: eye 1.5× as high as malar distance (Fig. 6A); Female OOL vs. LOL: OOL 1.9–2.1× as long as LOL (Fig. 7A); Antenna pilosity: thin, appressed, brownish (Figs 6A, 7A)…***Trissevania anemotis*** Kieffer, 1913**-** Facial striae presence: absent (Fig. 6B); Malar distance vs. eye height: eye 2.0× as high as malar distance (Fig. 6B); Female OOL vs. LOL: OOL 1.0–1.1× as LOL (Fig. 7B); Antenna pilosity: thick, semierect, whitish (Figs 6A, 7A)…**3**
3. Mesopleural carina count: present (Figs 8A, B); Proximodistal length of pedicel vs. proximodistal length of first flagellomere in male: pedicel at least as long as first flagellomere (Fig. 9B)…**4**-Mesopleural carina count: absent (Fig. 10A); Proximodistal length of pedicel vs. proximodistal length of first flagellomere in male: pedicel distinctly shorter than first flagellomere (Fig. 9A)…***Trissevania hugoi*** sp. nov.
4. Mesoscutellum length vs. anteromesoscutum length: mesoscutellum shorter than anteromesoscutum (Figs 11A, B); Dorsal area of the metapectal-propodeal complex median length vs. mesoscutellum median length: mesoscutellum 1.0–1.2× as long as metapectal-propodeal complex (Figs 11A, B); Head+mesosoma median length vs. mesosoma height: Head+mesosoma median length 1.8× as long as mesosoma height (Fig. 11B; also compare Figs 8A and 11A)…**5** **-** Mesoscutellum length vs. anteromesoscutum length: mesoscutellum longer than or equals anteromesoscutum (Figs 7A, B); Dorsal area of the metapectal-propodeal complex median length vs. mesoscutellum median length: mesoscutellum 1.4–1.6× as long as metapectal-propodeal complex (Figs 7A, B); Head+mesosoma median length vs. mesosoma height: 0.9–1.1× as long as mesosoma height (compare Figs 10A and B)…***Trissevania slideri*** sp. nov.
5. Dorsal margin of mesosoma lateral view shape: straight (Figs 8A, 11B)…***Trissevania mrimaensis*** sp. nov.**-** Dorsal margin of mesosoma lateral view shape: convex (Fig. 10A)…***Trissevania heatherae*** sp. nov.
6. Cranium color: black (Figs 6C, 12B). Antenna color female: scape, pedicel, flagellomeres 1–3 yellow, flagellomeres 4, 5 light brown, flagellomeres 6–11 brown; Petiole color: anterior region yellowish posterior region brown (Figs 4C, 10B, 12A); Female OOL vs. LOL: OOL 1.1× as long as OOL (Fig. 12A); Head width vs. IOS: head 1.8–2.0× as wide as IOS (Fig. 6C); Dorsal area of the metapectal-propodeal complex median length vs. mesoscutellum median length: mesoscutellum 1.0–1.2× as long as metapectal-propodeal complex (Fig. 12A); female petiole length vs. petiole width: 3.9–4.4× as long as wide; Male petiole length vs. petiole width: 4.9–5.2× as long as wide…***Afrevania longipetiolata*** sp. nov.**-** Cranium color: brown (Fig. 6D, 12B); Antenna color female: brown; Petiole color: brown; Female OOL vs. LOL: OOL 1.9–2.1× as long as LOL (Fig. 7A); Head width vs. IOS: head about 1.5× as wide as IOS (Fig. 6B). Dorsal area of the metapectal-propodeal complex median length vs. mesoscutellum median length: mesoscutellum 1.6–1.8× as long as metapectal-propodeal complex (Fig. 12B). Female petiole length vs. petiole width: 3.1× as long as wide. Male petiole length vs. width: 3.0× as long as wide…***Afrevania leroyi*** Benoit, 1951

***Trissevania*** **Kieffer, 1913**

*Trissevania*: Kieffer, J. J., 1913: 33 (original description)

#### Diagnosis

Fore wing vein 3RS structure: tubular (Fig. 5B). Fore wing vein r-rs structure: tubular (Fig. 5B).

#### Description

Cranium color: black. Head width vs. IOS: head 1.8–2.0× as wide as IOS. Mesosoma color: black tegula dark brown. Petiolar scrobe pubescence: absent. Fore wing vein r-rs presence: present. Fore wing vein 3RS presence: present. Female petiole length vs. petiole width: about 3× as long as wide. Petiole color: black. Gaster color: dark brown.

***Trissevania anemotis*** **Kieffer 1913**

(Figs 5B, 6A, 7A)

*Trissevania anemotis*: Kieffer, J. J. 1913: 33–34 (original description)

#### Diagnosis

Differs from all other *Trissevania* species in:

Facial striae count: present; Carinae laterally on frons count: present; Female OOL vs. LOL: OOL 1.9–2.1× as long as LOL; Antenna pilosity: thin, decumbent, brownish; Malar distance vs. eye height: eye 1.5× as high as malar distance.

#### Description

Body length: 2.4–3.0 mm. Mandible color: brown; black with dark brown mandibular teeth. Antenna color female: dark brown. Malar distance vs. eye height: eye 1.5× as high as malar distance. Facial striae count: present. Carinae laterally on frons count: present. Female OOL vs. LOL: OOL 1.9–2.1× as long as LOL. Antenna pilosity: thin, decumbent, brownish. Proximodistal length of pedicel vs. proximodistal length of first flagellomere in male: pedicel at least as long as first flagellomere. Dorsal margin of mesosoma lateral view shape: convex. Head+mesosoma median length vs. mesosoma height: Head+mesosoma median length 0.9–1.1× as long as mesosoma height. Mesoscutellum median length vs. anteromesoscutum median length: mesoscutellum longer than or equals anteromesoscutum. Mesopleural carina count: absent. Dorsal area of the metapectal-propodeal complex median length vs. mesoscutellum median length: mesoscutellum 1.5–1.6× as long as metapectal-propodeal complex. Male petiole length vs. width: 3.5–4.0× as long as wide. Median conjunctiva of male abdominal tergum 9 count: present.

#### Material Examined

Other material (9 males, 8 females): CAMEROON: 1 male. IM 5194 (PSUC). KENYA: 7 males, 8 females. NCSU 43257, 53297, 53299, 53504; PSUC_FEM 79773-79774 (PSUC); NCSU 43254-43256, 53503, 53505; PSUC_FEM 79770, 79772 (NMKE); NCSU 52051 (MRAC); NCSU 53298 (CAS).

#### Comments

Although the repository for type specimen(s) of *Trissevania anemotis* Kieffer, 1913 is currently unknown, we were able to recognize specimens of this species based on the original description. The only Trissevaniini species with malar striation is *T. anemotis* (in Kieffer's description [22]): “…joues fortement striées en long, convexes comme le front, sans sillon, égalant presque la longueur des yeux…”, which we translate as: “cheeks strongly striated all along, convex as the forehead, without ridges, almost as long as the eye's length”).

***Trissevania heatherae*** **sp. nov.**

*urn:lsid:zoobank.org:act:ACBF3813-5794-4E7F-9B5E-25A48CCBFB75*

#### Diagnosis

*Trissevania heatherae* sp. nov. shares the presence of the mesopleural carina and the elongate body (head+mesosoma median length 1.8× as long as mesosoma height) with *T. mrimaensis* sp. nov. and differs from it in the convex dorsal side of the mesosoma (*T. mrimaensis* is the only Trissevaniini with the dorsal side of the mesosoma flat).

#### Description

Body length: 2.6–2.7 mm. Mandible color: black with dark brown mandibular teeth. Malar distance vs. eye height: eye 2× as high as malar distance. Facial striae count: absent. Carinae laterally on frons count: absent. Antenna pilosity: thick, semierect, whitish; thin, decumbent, brownish. Proximodistal length of pedicel vs. proximodistal length of first flagellomere in male: pedicel at least as long as first flagellomere. Dorsal margin of mesosoma lateral view shape: convex. Head+mesosoma median length vs. mesosoma height: Head+mesosoma median length 1.8× as long as mesosoma height. Mesoscutellum median length vs. anteromesoscutum median length: mesoscutellum shorter than anteromesoscutum. Mesopleural carina count: present. Dorsal area of the metapectal-propodeal complex median length vs. mesoscutellum median length: mesoscutellum 1.0–1.2× as long as metapectal-propodeal complex. Male petiole length vs. width: 3.5–4.0× as long as wide.

#### Material examined

Holotype male: KENYA, Coast Prov. Mrima Hill Forest 4.48576S, 39.25845E 212m Malaise trap, indigenous forest edge 8–22 Aug 2011 R. Copeland, PSUC_FEM 79769 (deposited in NMKE). Paratypes (3 males): KENYA: 3 males. PSUC_FEM 79766–79767 (PSUC); PSUC_FEM 79768 (NMKE).

#### Etymology

The species epithet “heatherae” honors Dr. Heather M. Hines, professor of Biology and Entomology at Pennsylvania State University, University Park, PA, USA.

***Trissevania hugoi*** **sp. nov.**

*urn:lsid:zoobank.org:act:B1466AB4-42B2-4972-82B5-0BD754E4AD7A*

#### Diagnosis

*Trissevania hugoi* sp. nov. differs from all other *Trissevania* species in: Proximodistal length of pedicel vs. proximodistal length of first flagellomere in male: pedicel distinctly shorter than first flagellomere. The species shares the presence of the mesopleural carina with *Trissevania mrimaensis* and *T. heatherae* and differs from them in: Head+mesosoma median length vs. mesosoma height: Head+mesosoma median length 0.9–1.1× as long as mesosoma height. Mesoscutellum median length vs. anteromesoscutum median length: mesoscutellum longer than or equals anteromesoscutum.

#### Description

Body length: 2.0–2.3 mm. Mandible color: black with dark brown mandibular teeth. Antenna color female: brown. Malar distance vs. eye height: eye 2× as high as malar distance. Facial striae count: absent. Carinae laterally on frons count: absent. Female OOL vs. LOL: OOL 1.0–1.2× as long as LOL. Antenna pilosity: thick, semierect, whitish. Proximodistal length of pedicel vs. proximodistal length of first flagellomere in male: pedicel distinctly shorter than first flagellomere. Dorsal margin of mesosoma lateral view shape: convex. Head+mesosoma median length vs. mesosoma height: Head+mesosoma median length 0.9–1.1× as long as mesosoma height. Mesoscutellum median length vs. anteromesoscutum median length: mesoscutellum longer than or equals anteromesoscutum. Mesopleural carina count: absent. Dorsal area of the metapectal-propodeal complex median length vs. mesoscutellum median length: mesoscutellum 1.4–1.5× as long as metapectal-propodeal complex. Male petiole length vs. width: 3.0× as long as wide. Median conjunctiva of male abdominal tergum 9 count: present.

#### Material examined

Holotype female: BURUNDI, Ruvubu NP, 1382 m, 2.98144°S, 30.45531°E, Malaise trap 5–21 JAN 2010, R Copeland, Photo 1186–1189, E11, NCSU 53509 (deposited in NMKE). Paratypes (3 males): BURUNDI: NCSU 43252 (PSUC); NCSU 43283; PSUC_FEM 79771 (NMKE).

#### Etymology

The species epithet honors Hugo W. Deans.

***Trissevania mrimaensis*** **sp. nov.**

*urn:lsid:zoobank.org:act:3FFF55E8-B090-464C-A758-8B7CB516D7A7*

(Figs 8, 11)

#### Diagnosis

*Trissevania mrimaensis* shares the following character states with *T. heatherae*: Mesoscutellum median length vs. anteromesoscutum median length: mesoscutellum shorter than anteromesoscutum; Dorsal area of the metapectal-propodeal complex median length vs. mesoscutellum median length: mesoscutellum 1.0–1.2× as long as metapectal-propodeal complex. It differs from *Trissevania heatherae* in: Head+mesosma median length 1.8× as long as mesosoma height.

#### Description

Body length: 2.6–2.7 mm. Mandible color: brown. Antenna color female: dark brown. Malar distance vs. eye height: eye 2× as high as malar distance. Facial striae count: absent. Carinae laterally on frons count: absent. Female OOL vs. LOL: OOL 1.0–1.2× as long as LOL. Antenna pilosity: thick, semierect, whitish. Proximodistal length of pedicel vs. proximodistal length of first flagellomere in male: pedicel at least as long as first flagellomere. Dorsal margin of mesosoma lateral view shape: straight. Head+mesosoma median length vs. mesosoma height: Head+mesosoma median length 1.8× as long as mesosoma height. Mesoscutellum median length vs. anteromesoscutum median length: mesoscutellum shorter than anteromesoscutum. Mesopleural carina count: present. Dorsal area of the metapectal-propodeal complex median length vs. mesoscutellum median length: mesoscutellum 1.0–1.2× as long as metapectal-propodeal complex. Male petiole length vs. width: 3.5–4.0× as long as wide.

#### Material Examined

Holotype female: KENYA, Coast Prov. Mrima Hill Forest 4.48576S, 39.25845E 212 m Malaise trap, indigenous forest edge 11–25 July 2011 R. Copeland 14482-TrissevaniaB12, NCSU 43253 (NMKE). Paratype (male): KENYA, PSUC_FEM 79765 (NMKE).

#### Etymology

The name of the new species refers to the locality of the holotype: Mrima Hill Forest in Kenya.

***Trissevania slideri*** **sp. nov.**

*urn:lsid:zoobank.org:act:7E0BB974-4891-413A-8C23-32C274B30F19*

(Fig. 6B, 7B, 9A)

#### Diagnosis

*Trissevania slideri* sp. nov. shares the absence of the facial striae and the short mesosoma (mesosoma height equals head+mesosoma length) with *Trissevania hugoi* sp. nov. and differs from it in the presence of mesopleural carina and the pedicel as long as the first flagellomere (*T. hugoi* is the only Trissevaniini with the first flagellomere being distinctly longer than pedicel (almost 2× as long as pedicel)).

#### Description

Body length: 2.0–2.3 mm. Mandible color: brown. Antenna color female: brown. Malar distance vs. eye height: eye 2× as high as malar distance. Facial striae count: absent. Carinae laterally on frons count: absent. Female OOL vs. LOL: OOL 1.0–1.2× as long as LOL. Antenna pilosity: thick, semierect, whitish. Proximodistal length of pedicel vs. proximodistal length of first flagellomere in male: pedicel at least as long as first flagellomere. Dorsal margin of mesosoma lateral view shape: convex. Head+mesosoma median length vs. mesosoma height: Head+mesosoma median length 0.9–1.1× as long as mesosoma height. Mesoscutellum median length vs. anteromesoscutum median length: mesoscutellum longer than or equals anteromesoscutum. Mesopleural carina count: present. Dorsal area of the metapectal-propodeal complex median length vs. mesoscutellum median length: mesoscutellum 1.6–1.8× as long as metapectal-propodeal complex. Male petiole length vs. width: 3.0× as long as wide.

#### Material examined

Holotype male: BURUNDI, Rusizi Nat. Pk. Degraded bush/grassland 774 m, 3.34364° S, 29.27246° E Malaise trap, near 3 small trees 5–19 DEC 2009 R. Copeland, NCSU 53507 (deposited in NMKE). Paratypes (1 female, 1 male): BURUNDI: 1 female, 1 male. NCSU 53506 (PSUC); NCSU 53508 (NMKE).

#### Etymology

The species epithet honors David “Slider” Love, coordinator of Farm and Greenhouse Operations at the Department of Entomology at the Pennsylvania State University, for his extensive work in helping to revitalize the Frost Entomological Museum.

***Afrevania*** **Benoit 1953**

*Afrevania*: Benoit, P. L. G. 1953: 259 (original description)

#### Diagnosis

Fore wing vein 3RS structure: not tubular, marked by dark line (Fig. 2B). Fore wing vein r-rs structure: not tubular, marked by dark line (Fig. 2B).

#### Description

Median clypeal projection presence: present. Median clypeal projection sharpness: blunt. Malar distance vs. eye height: eye 2× as high as malar distance. Facial striae presence: absent. Carinae laterally on frons presence: absent. Antenna pilosity: thin, appressed, brownish. Male apical flagellomere shape: about 3× as long as wide. Mesosoma color: black tegula light brown. Dorsal margin of mesosoma lateral view shape: convex. Head+mesosoma median length vs. mesosoma height: Head+mesosma median length 0.9–1.1× as long as mesosoma height. Mesoscutellum median length vs. anteromesoscutum median length: mesoscutellum longer than or equals anteromesoscutum. Mesopleural carina presence: absent. Petiolar scrobe pubescence: present. Fore wing vein r-rs presence: absent. Fore wing vein 3RS presence: absent. Gaster color: light brown.

***Afrevania*** **leroyi Benoit, 1953**

(Figs 6D, 12B)

*Afrevania leroyi*: Benoit, 1953: 259 (original description) holotype female, deposited at MRAC, labels: “Nord du lac Kivu: Rwankwi 15-XI-1950 (J. V. Leroy)”

#### Description/Diagnosis

Cranium color: dark brown. Head width vs. IOS: head about 1.5× as wide as IOS. Female OOL vs. LOL: OOL 1.9–2.1× as long as LOL. Antenna color female: brown. Dorsal area of the metapectal-propodeal complex median length vs. mesoscutellum median length: mesoscutellum 1.6–1.8× as long as metapectal-propodeal complex. Female petiole length vs. petiole width: 2.2–3.1× as long as wide. Petiole color: brown. Male petiole length vs. width: 2.5× as long as wide. Body length: 1.8–1.9 mm.

#### Material Examined

Holotype female: DEMOCRATIC REPUBLIC OF CONGO: Nord du lac Kivu: Rwankwi, 15.11.1950, J. V. Leroy, (deposited in MRAC). Other material (1 female, 1 male): CONGO: 1 female. NCSU 53950 (MRAC). SOMALIA: 1 male. NCSU 52616 (ISNB).

***Afrevania longipetiolata*** **sp. nov.**

*urn:lsid:zoobank.org:act:273AF41D-A10C-4A51-916C-6A40D56A9598*

(Figs 1B, 2A–C, 3A–C, 4A–D, 6C, 10A–B, 12A)

#### Description/Diagnosis

Cranium color: black. Head width vs IOS: head 1.8–2.0× as wide as IOS. Female OOL vs LOL: OOL 1.0–1.1× as long as LOL. Antenna color female: scape, pedicel, flagellomeres 1–3 yellow, flagellomeres 4, 5 light brown, flagellomeres 6–11 brown. Dorsal area of the metapectal-propodeal complex median length vs. mesoscutellum median length: mesoscutellum 1.0–1.2× as long as metapectal-propodeal complex. Female petiole length vs. petiole width: 3.9–4.4× as long as wide. Petiole color: anterior region yellowish posterior region brownish. Male petiole length vs. width: 4.9–5.2× as long as wide. Median conjunctiva of male abdominal tergum 9 count: present. Body length: 2.4–2.6 mm.

#### Material Examined

Holotype female: SOUTH AFRICA: Ramsgate Butterfly Sanctuary, nr. stream, 3–30.10.2004, M. Mostovski, NCSU 2330 (deposited in SAMC). Paratypes (24 males, 10 females): SOUTH AFRICA: 24 males, 10 females. NCSU 42244–42246 (MRAC); NCSU 2309–2310, 2313, 2329, 2331 (NMSA); NCSU 2328, 18846, 42243, 42247–42248, 42250, 51866, 52052, 53951 (PSUC); NCSU 2308, 2314, 2333–2334, 42249 (SANC); NCSU 2306, 2335, 53953–53955 (CNC); NCSU 2307, 2311–2312, 53952 (SAMC); NCSU 2305, 2332, 42242 (CAS). Other material (1 male): SOUTH AFRICA: 1 male. NCSU 53138 (PSUC).

#### Etymology

The species name refers to the elongated petiole relative to other Trissevaniini.

### Morphological observations on the Trissevaniini fore wing

Although we were unable to observe live specimens, we were able to move and manipulate the fore wings of glycerin-stored specimens, transforming them between the “fully folded” (Fig. 2C) and “fully unfolded” (Fig. 2B) positions.

The wing blade is “collapsed” (Fig. 4C) along the dorsally convex anal-marginal fold line in most available specimens. We were able to force the wings from this “collapsed” position into a stable, “fully unfolded” position by gently pressing the area of the anal-marginal fold line (afl), delimited proximally by the intersection with the prestigmal fold line (pfl) and distally by the intersection with the poststigmal fold line (sfl) and the discal fold line (dfl) (stiffening region: Fig. 2A). This region is concave when the wing is in the “fully unfolded” (Fig. 2B) but convex if the wing is in the “collapsed” (Fig. 4C) or “fully folded” (Fig. 2C) positions. We could not force the wing blade into a stable “fully folded” position (Fig. 2C) because it always restored itself to the “collapsed” position. In order to achieve a complete wing folding the wing must be folded first along the longitudinal fold line (afl: Figs 5, S1) and then along the transverse fold line (sfl+dfl: Figs 5, S1). The surface of the back-folded distal wing region (composed of the wing regions distal to sfl and dfl on Figs 5, S1) is curved in the fully folded wing. This curved region fits well in-between the anteroventral portion of the gaster and the posterior concavity on the mesosoma that receives the gaster (gastral scrobe). In two dried specimens with fully folded wings, they were folded in the same way, with the back-folded distal wing region tightly packed between the gaster and the mesosoma (Figs 10A, B).

While maneuvering the fore wing of glycerin-stored specimens, we observed that the posterior fore wing margin was often hitched to the dorsolateral part of the mesosoma. In these cases, setae of the dorsolateral setal patch (dsp: 3C, 4A, C) were inserted into the retinaculum (ventrally curved, gutter-like region on the posterior fore wing margin) (ret: Fig. 4B, 5A, B). Based on dissection of the mesosoma of critical point dried specimens, Trissevaniini shares the characteristics of the direct wing muscles (including the mesopleuro-basalare muscle) of other Evaniidae (fig. 10D in [10]), and we were unable to define any differences in the trissevaniine wing base relative to other Evaniidae (Figs 1A, B). Our CLSM studies with the 405 nm laser show that fold lines on the fore wing of *Afrevania longipetiolata* are resilin rich (Fig. 2A).

## Discussion

### Unresolved phenotypes and the compatibility of semantic statements

Fully representing the meaning of NL descriptions is impossible with current ontologies and OWL syntaxes. Textualizing our phenotype concepts with any syntax, however, limits expressivity, as even NL descriptions need incremental improvements. Although we have refined the semantics of numerous statements that were proposed in earlier taxonomic treatments [16], [12], there remain many statements in need of further refinement (e.g., the HAO lacks many wing part classes).

This situation highlights an important issue related to the longevity of semantic statements: How can we trace refinements in Manchester syntax expressions of the same phenotype statement during the evolution of ontologies? Is it possible to connect specimens from earlier treatments to the new, refined statements? We propose here a simple mechanism to reattach refined statements to already published specimen data (see Materials and Methods). The resulting equivalence OWL file could be considered a long-term repository for further taxonomic treatments, and it provides the backbone for a semantic model system that meets the requirements of emerging eScience applications [23].

### Trissevaniini and the evaniid phylogeny

Despite recent advances in evaniid systematics the phylogeny of Evaniidae is still unstable. Trissevaniini appears to be sister to *Evania*, based on analyses that included only one exemplar of the tribe, *Trissevania anemotis* [24]. Morphology seemingly contradicts these results. *Evania* spp. are relatively large (body length = 4–5 mm), elongate species with perhaps the most complex evaniid wing venation pattern (more than 10 cells on the fore wing). By contrast, genera of Trissevaniini are small (body length = 1–2 mm), stocky wasps, with less than 3 cells on the fore wing. The fore wing itself is relatively wider than those of the larger evaniids [8]. Apart from these general habitus (*Gestalt*) characters, Trissevaniini share numerous distinct phenotypes that appear to place them close to the genus *Brachygaster*: presence of posterior tooth of mandible (ptn: Fig. 3B), presence of profemoral scrobe on the mesopectus (psm: Figs 3C, 8A, B), presence of cranial scrobe and limiting scrobal carina on the mesonotum (sca: Figs 3C, 7A, 11B), the carina delimiting ventrally the anterior region of prespecular sulcus (cds: Figs 3C, 8B) the absence of the transmetapectal carina (Figs 3C, 4A, 11B; although the carina is present in some *Brachygaster* species), and the elongate anterior tentorial pit (atp: Fig. 3A). Most of these traits, however, may be driven by spatial environmental constraints like the host geometry (scrobes on the mesosoma accommodate appressed appendages and the head) and might be evolutionary adaptations [25].

Evolutionary adaptation of the above listed morphological traits in Trissevaniini and *Brachygaster* is also supported by the differences in fore wing venation pattern between these taxa. The point-like or short and anterodistally-oriented fore wing 1CUa vein, and the absence of fore wing 1RS vein and the 1^st^ submarginal cell are shared by many short bodied evaniid taxa, such as *Rothevania* (Fig. 5C), *Brachygaster* (Fig. 5D) and *Semaeomyia*. Trissevaniini share the posteriorly oriented fore wing 1CUa vein and the presence of 1RS and RS+M and 2RS veins (Fig. 5B) with large bodied Evaniidae such as *Evania* (Fig. 5A) and *Szepligetella*.

Besides Trissevaniini, there is only one evaniid genus with the apparent ability to fold its wings, *Brachevania*. However, *Brachevania* has two parallel wing folds (fig. 10 in [8]), and most importantly, does not share any of the diagnostic characters of the new tribe (e.g., the presence of dorsolateral setal patch of the metapectal-propodeal complex). *Brachevania* is monotypic and known only from one specimen. This situation limits further morphological examination.

### Homology of wing creases across Evaniidae

The presence and pattern of wing folding/flexion lines are relatively conservative, and thus these structures render themselves to homologization across Hymenoptera [7], [5], [26]. According to Mason [27], only concave flexion lines are represented in Hymenoptera, so flexion lines are easy to differentiate from the always convex wing veins even if they are not tubular or less melanized. Wootton [1], however, reported a convex flexion line from Hymenoptera (median flexion line; mfl: Figs 2A, 5A, B) extending just posterior of the Sc+R vein on the fore wing. This flexion line was somehow ignored in the aforementioned works. Nevertheless, besides the convex median flexion line (*sensu* Wootton [1], five flexion lines can be present in Hymenoptera: the claval flexion line (cfl: Figs 2A, 5A, B) extending just anteriorly of the first anal vein, the anterior (arf: Fig. 5A) and posterior radial flexion lines (prf: Fig. 5A) extending between the medial and radial veins and the anterior and posterior medial flexion lines (sensu Mason [27])  =  medial folds sensu Richards [7]), posterior to the median vein (not illustrated; the anterior and posterior flexion lines are absent from Evaniidae). Unlike flexion lines, the single fold line of some Hymenoptera is always convex above (similarly to wing veins and Wootton's median flexion line), and thus the ventral wing surfaces are closed together during the wing folding process [5]. These straight wing folds are apparently not homologous with wing veins and flexion lines.

Homologizing the fold lines of Trissevaniini is seemingly difficult, due to the distally reduced wing venation of the new tribe (Figs 5A, B, S1). Based on their position relative to the pterostigma and medial vein, however, it is possible that the prestigmal flexion line is derived from the stigmal break [28] of other Hymenoptera, the poststigmal crease from the anterior radial flexion line, and the discal crease from the posterior radial flexion line of other Evaniidae (arf, prf: Fig. 5A). We were unable to find any crease on the fore wing of other Hymenoptera from which we could derive the anal-marginal fold line. The evaniid fore wing 4RS vein and the anterior radial flexion line [26] are bent and oriented anteriorly (4RS, arf: Fig. 5A). This phenotype is unique to Evaniidae and might favor the development of the transverse wing fold that extends parallel to the anteriorly curved portion of 4RS (dfl, sfl: Fig. 5B).

### Possible wing folding/unfolding mechanism of Trissevaniini

Some Blattodea, Dermaptera, and Coleoptera have four plane wing folding on their hind wings, primarily for reducing the exposed wing blade area so that it can be tucked under the modified fore wing (elytron or tegmin; see also [6]). These orders share a basic wing fold pattern, in which four flexible fold lines intersect in one point and delimit four stiff plates, but their wing folding/unfolding strategies are different. For example, hind wing folding/unfolding is facilitated by modified direct wing muscles and wing base sclerites in beetles allowing them to promote/remote and unfold/fold their wings independently [29] while the wings are unfolded and folded solely by wing promotion and remotion in Blattodea [6] and in some Dermaptera [30]. The simplest wing folding mechanism and fold pattern are arguably that of the Blattodea. In this taxon the hind wing blade is folded longitudinally and then, in some species (e.g. Blaberidae), the apical region is folded over resulting in a small part of the wing tip lying on top of the folded wing [6]. This fold pattern consists of three convex and one concave folds and is named “EXT+” by Haas and Wootton (fig. 2 in [31]). Until now, this simple pattern of wing folds was only known from some blaberid species [31].

Since we were not able to detect any differences between the wing base and direct wing muscles of Trissevaniini and that of other Evaniidae, it is the most likely that wings are folded/unfolded during promotion and remotion, similar to Blattodea and Dermaptera. The basic pattern of Trissevaniini wing folds is most similar to that of Blattodea (Figs 2, 5, S1), representing the second known case of “EXT+” arrangement of wing folds [31].

Locking mechanisms play important roles in securing an unfolded wing position, a necessary condition for flight [5]. Wing regions that are involved in these mechanisms are usually at the intersection of concave and convex wing creases [5]. The stiffening region for the locking mechanism of trissevaniine fore wing is most likely on the anal-marginal fold line (stiffening region: Fig. 2A). The prestigmal fold line and the posterior radial fold line are not part of the main four plane folding line system, though they seem to play important roles in the locking mechanism. Without these creases the stiffening region would be restricted to one point at the intersection of the longitudinal fold and the transverse fold (intersection of sfl, dfl and afl on Fig. 5), which would result in a less stable unfolded position (Fig. S1).

High resilin content is necessary but not sufficient for the complete wing folding mechanism in Trissevaniini. External forces are required to keep the wing in the fully folded position (we were unable to find any wing blade based stiffening mechanism that stabilized the wing in a fully folded position), as well as for transforming it between the “fully folded” and “fully unfolded” positions. Based on our observations of specimens with fully folded wings, it is likely that the wing is secured in the fully folded position by being packed between the gaster and the mesosoma (Figs 10A, B). Dislodging the wing from its folded position does not require much force, and thus the hind legs can probably initiate this activity. Reaching a stable, unfolded position, however, appears to be a more involved process that most likely requires the blade to be anchored at some points. The dorsolateral setal patch and the retinaculum of the fore wing serves most like as the anchoring device in the wing folding system of Trissevaniini (dsp: Figs 3C, 4A, C).

### The cooption of the retinaculum

The presence of a dorsolateral setal patch (dsp: Figs 3, 4) is unique for the tribe. We hypothesize that this evolutionary novelty evolved in concert with the four plane wing folding mechanism in Trissevaniini. The retinaculum (ret: Figs 3, 4) is present in all winged Hymenoptera and is involved in wing coupling [32]. In Hymenoptera, a row of hook-like setae on the anterior hind wing margin, the hamuli, inserts into the retinaculum, locking the fore and hind wings together during the flight [33]. The involvement of the retinaculum in wing unfolding is an example of a morphological exaptation [34]. That is, the structure was produced by natural selection for wing coupling function and was then co-opted for wing folding function.

### Conservation status of Kenyan Trissevania species

The specimens of *Trissevania* treated herein were collected as part of a widespread Malaise trap survey of Kenyan insects conducted between 2004 and 2012. They were found across the breadth of Kenya from coastal forest in the east to highland forests in the central and far western parts of the country. Wasps occurred at altitudes ranging from near sea level on the coast (Mrima Hill, 212 m above sea level) to 2507 m in the central highlands (Itieni Forest, Nyambene Hills) and 2474 m on Mt Elgon in western Kenya. They were also collected in Burundi in Malaise traps set in degraded shrub/grassland (Rusizi National Park, alongside Lake Tanganyika, 774 m), in mid-altitude gallery forest (Ruvubu National Park, 1382 m), and in Bururi National Forest at 1955 m.

The conservation status of the five Kenyan sites differs greatly. Itieni forest is managed by the Kenya Forest Service and is largely intact and under little threat from about 2100 m to over 2500 m. Mt. Elgon Forest lies within the national park and is also well protected. Gatamayu Forest is in the southernmost part of the Aberdare range, which has recently been completely fenced, and Castle Forest is above the altitude at which farming is permitted on the southern slope of Mt. Kenya. In contrast, the status of forest on Mrima Hill is precarious. Mrima Hill Forest (ca. 390 ha.) is part of the Coastal Forests of Eastern Africa biodiversity hotspot [35] comprising mostly small, isolated relict forests stretching from southern Somalia into Mozambique, and known for its high level of endemicity, with about 768 described endemic plants and animal species [36]. Many of these forests, including that of Mrima, are kaya, sites associated with religious and cultural rites of the coastal Mijikenda peoples. Recent gazettement of several kayas as National Monuments has brought them under the joint care and supervision of the National Museums of Kenya and the Kenya Forest Service. This and the past cultural importance of kayas have been largely responsible for their conservation in an otherwise vast expanse of farmland.

However, these small forests are under increasing pressure due to the declining influence of traditional customs in the face of westernization and the ever-present demands of a growing human population. Additionally, significant damage of a type specific to Mrima Hill Forest has accrued in the past few years, a result of invasive mining techniques (forest clearing for road building, hole drilling) by a foreign company hoping to extract Niobium, a rare earth mineral [37]. The operating license for this company has now been revoked. Nonetheless, we consider *Trissevania mrimaensis* and *T. heatherae* to be vulnerable (they meet criterion “B” of Vulnerable (VU) IUCN red list category [38]). Most coastal forest endemics are relict species, and except for a few plant and bird species, all have a very limited distribution even within the network of coastal forests [36].

Mrima Hill Forest is also home to several other interesting insect taxa. Recently two undescribed species of Megalyridae (Hymenoptera), a rarely collected wasp family, were sampled in Mrima (S. Shaw, unpublished data) as was a species of *Rhipidioides* (Coleoptera: Rhipiphoridae) (Z. Falin, unpublished data), a genus previously known only from Australia. Hopefully the discovery of these and other endemics such as *Trissevania mrimaensis* and *T. heatherae* will help build a case for increased vigilance in protecting Mrima and other coastal forest habitats.

## Supporting Information

Figure S1 **Print, cut and fold model of the evaniid fore wing (** ***Trissevania anemotis*** **on the left,** ***Evania albofascialis*** **on the right, anterior to the right).** Convex wing creases are marked as mountain folds whereas concave ones as valley folds following the origami terminology. The wings should be cut along their margin and folded along the mountain and valley folds. The model demonstrates the wing folding mechanism of Trissevaniini and the complexity of fold and flexion line system of the tribe relative to other Evaniidae. The necessity and possible function of each crease can be demonstrated by comparing the cut outs: 1. wings without folded median flexion line (mfl) and claval flexion line (cfl) lack the “Z-shape profile” and bend at the basal region when the blade is moved; 2. Wing locking is restricted at the intersection of transverse and longitudinal wing folds and results an unstable folded position if the prestigmal fold line (pfl) and the posterior radial flexion line (prf) are not folded. Crease first the longitudinal fold line (afl) and after that the transverse fold (sfl+pfl) for the accurate four plane wing folding!
(TIF)

Table S1 **Specimens examined.**
(DOCX)

Table S2 **List of abbreviations of anatomical structures applied on Figures.**
(DOCX)

Table S3 **Natural language representations of phenotypes in EQ format (Entity attribute: value) with comments.**
(DOCX)

Table S4 **Semantic representations of phenotypes in Manchester syntax format from different taxonomic treatments of Evaniidae (Mullins et al. 2012, Balhoff et al. 2013 and the present taxonomic treatment).**
(DOCX)
