# Supplementary figures and images for: Folding Wings like a Cockroach: A Review of Transverse Wing Folding Ensign Wasps (Hymenoptera: Evaniidae: Afrevania and Trissevania)
Source: PLoS One. 2014 May 2;9(5):e94056. doi: 10.1371/journal.pone.0094056 (PMC4008374; doi:10.1371/journal.pone.0094056)

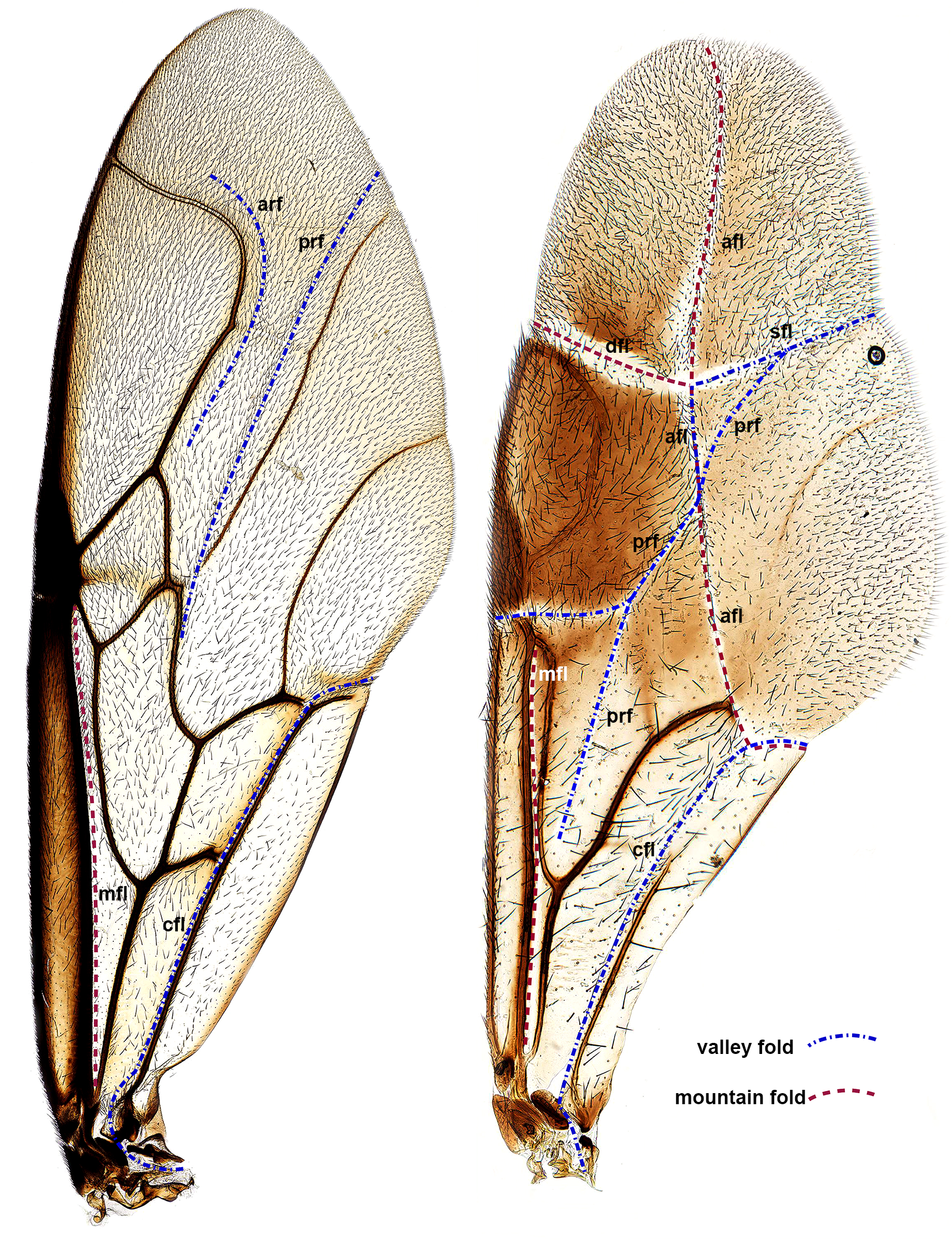

Supplement: Figure S1 — Print, cut and fold model of the evaniid fore wing ( Trissevania anemotis on the left, Evania albofascialis on the right, anterior to the right). Convex wing creases are marked as mountain folds whereas concave ones as valley folds following the origami terminology. The wings should be cut along their margin and folded along the mountain and valley folds. The model demonstrates the wing folding mechanism of Trissevaniini and the complexity of fold and flexion line system of the tribe relative to other Evaniidae. The necessity and possible function of each crease can be demonstrated by comparing the cut outs: 1. wings without folded median flexion line (mfl) and claval flexion line (cfl) lack the “Z-shape profile” and bend at the basal region when the blade is moved; 2. Wing locking is restricted at the intersection of transverse and longitudinal wing folds and results an unstable folded position if the prestigmal fold line (pfl) and the posterior radial flexion line (prf) are not folded. Crease first the longitudinal fold line (afl) and after that the transverse fold (sfl+pfl) for the accurate four plane wing folding! (TIF) [file pone.0094056.s001.tif]
