# Supplementary material for: Folding Wings like a Cockroach: A Review of Transverse Wing Folding Ensign Wasps (Hymenoptera: Evaniidae: Afrevania and Trissevania)
Source: PLoS One. 2014 May 2;9(5):e94056. doi: 10.1371/journal.pone.0094056 (PMC4008374; doi:10.1371/journal.pone.0094056)
Supplement: Table S1 — Specimens examined. (DOCX) [file pone.0094056.s002.docx]

**Table S1.** Specimens examined.

*Afrevania longipetiolata* sp. nov.

| **Identifier(s)** | **Repository** | **Sex** | **Collecting event** |
| --- | --- | --- | --- |
| NCSU 2335 | CNC | male | S. AFRICA: KwaZulu- Natal Ramsgate Butterfly Sanctuary; 3-30.x.2004 30º53.3'S 30º24.4'E; MT nr. stream; M. Mostovski |
| NCSU 2334 | SANC | female | S. AFRICA: KwaZulu- Natal Ramsgate Butterfly Sanctuary; 3-30.x.2004 30º53.3'S 30º24.4'E; MT nr. stream; M. Mostovski |
| NCSU 2328 | PSUC | male | S. AFRICA: KwaZulu- Natal Ramsgate Butterfly Sanctuary; 3-30.x.2004 30º53.3'S 30º24.4'E; MT nr. stream; M. Mostovski |
| NCSU 2329 | NMSA | male | S. AFRICA: KwaZulu- Natal Ramsgate Butterfly Sanctuary; 3-30.x.2004 30º53.3'S 30º24.4'E; MT nr. stream; M. Mostovski |
| NCSU 2330 | SAMC | female | S. AFRICA: KwaZulu- Natal Ramsgate Butterfly Sanctuary; 3-30.x.2004 30º53.3'S 30º24.4'E; MT nr. stream; M. Mostovski |
| NCSU 2331 | NMSA | male | S. AFRICA: KwaZulu- Natal Ramsgate Butterfly Sanctuary; 3-30.x.2004 30º53.3'S 30º24.4'E; MT nr. stream; M. Mostovski |
| NCSU 2332 | CAS | male | S. AFRICA: KwaZulu- Natal Ramsgate Butterfly Sanctuary; 3-30.x.2004 30º53.3'S 30º24.4'E; MT nr. stream; M. Mostovski |
| NCSU 2333 | SANC | female | S. AFRICA: KwaZulu- Natal Ramsgate Butterfly Sanctuary; 3-30.x.2004 30º53.3'S 30º24.4'E; MT nr. stream; M. Mostovski |
| NCSU 2305 | CAS | male | S. AFRICA: Kwa-Zulu Natal: Ramsgate Butterfly Sanctuary;30º53S 30º20E 1.xi-2.xii.2004; MT; near stream; M Mostovski |
| NCSU 2306 | CNC | male | S. AFRICA: Kwa-Zulu Natal: Ramsgate Butterfly Sanctuary;30º53S 30º20E 1.xi-2.xii.2004; MT; near stream; M Mostovski |
| NCSU 2307 | SAMC | female | S. AFRICA: Kwa-Zulu Natal: Ramsgate Butterfly Sanctuary;30º53S 30º20E 1.xi-2.xii.2004; MT; near stream; M Mostovski |
| NCSU 2308 | SANC | female | S. AFRICA: Kwa-Zulu Natal: Ramsgate Butterfly Sanctuary;30º53S 30º20E 1.xi-2.xii.2004; MT; near stream; M Mostovski |
| NCSU 2309 | NMSA | male | S. AFRICA: Kwa-Zulu Natal: Ramsgate Butterfly Sanctuary;30º53S 30º20E 1.xi-2.xii.2004; MT; near stream; M Mostovski |
| NCSU 2310 | NMSA | male | S. AFRICA: Kwa-Zulu Natal: Ramsgate Butterfly Sanctuary;30º53S 30º20E 1.xi-2.xii.2004; MT; near stream; M Mostovski |
| NCSU 2311 | SAMC | female | S. AFRICA: Kwa-Zulu Natal: Ramsgate Butterfly Sanctuary;30º53S 30º20E 1.xi-2.xii.2004; MT; near stream; M Mostovski |
| NCSU 2312 | SAMC | female | S. AFRICA: Kwa-Zulu Natal: Ramsgate Butterfly Sanctuary;30º53S 30º20E 1.xi-2.xii.2004; MT; near stream; M Mostovski |
| NCSU 2313 | NMSA | male | S. AFRICA: Kwa-Zulu Natal: Ramsgate Butterfly Sanctuary;30º53S 30º20E 1.xi-2.xii.2004; MT; near stream; M Mostovski |
| NCSU 2314 | SANC | female | S. AFRICA: Kwa-Zulu Natal: Ramsgate Butterfly Sanctuary;30º53S 30º20E 1.xi-2.xii.2004; MT; near stream; M Mostovski |
| NCSU 53138 | PSUC | male | S. AFRICA: KwaZulu- Natal Ramsgate Butterfly Sanctuary; 3-30.x.2004 30º53.3'S 30º24.4'E; MT nr. stream; M. Mostovski |
| NCSU 52052 | PSUC | male | SOUTH AFRICA: KwaZulu- Natal: Hluhluwe Game Reserve; ix.1997; MT; JGH Londt |
| NCSU 53951 | PSUC | male | S. AFRICA: KwaZulu- Natal: Ramsgate Butterfly Sanctuary; MT nr stream 30º53.3S 30º20.4E; 9.i- 2.ii.2005; M Mostowski |
| NCSU 53955 | CNC | male | S.AFRICA:KwaZulu-Natal Queen Elizabeth Park Res. 29º34S 30º19'E; 1.xii.2003 J Londt & M Mostovski |
| NCSU 53954 | CNC | male | S.AFRICA:KwaZulu-Natal Queen Elizabeth Park Res. 29º34S 30º19'E; 1.xii.2003 J Londt & M Mostovski |
| NCSU 53953 | CNC | male | SOUTH AFRICA, KwaZulu-Natal Hiuhluwe Game Reserve: Camp JGH Londt |
| NCSU 53952 | SAMC | female | SOUTH AFRICA: KwaZulu- Natal: Hluhluwe Game Reserve; ix.1997; MT; JGH Londt |
| NCSU 42250 | PSUC | female | S. AFRICA: KwaZulu- Natal Ramsgate Butterfly Sanctuary; 3-30.x.2004 30º53.3'S 30º24.4'E; MT nr. stream; M. Mostovski |
| NCSU 42249 | SANC | female | S. AFRICA: KwaZulu- Natal Ramsgate Butterfly Sanctuary; 3-30.x.2004 30º53.3'S 30º24.4'E; MT nr. stream; M. Mostovski |
| NCSU 42242 | CAS | male | S. AFRICA: KwaZulu- Natal Ramsgate Butterfly Sanctuary; 3-30.x.2004 30º53.3'S 30º24.4'E; MT nr. stream; M. Mostovski |
| NCSU 42243 | PSUC | male | S. AFRICA: KwaZulu- Natal Ramsgate Butterfly Sanctuary; 3-30.x.2004 30º53.3'S 30º24.4'E; MT nr. stream; M. Mostovski |
| NCSU 42244 | MRAC | male | S. AFRICA: KwaZulu- Natal Ramsgate Butterfly Sanctuary; 3-30.x.2004 30º53.3'S 30º24.4'E; MT nr. stream; M. Mostovski |
| NCSU 42245 | MRAC | male | S. AFRICA: KwaZulu- Natal Ramsgate Butterfly Sanctuary; 3-30.x.2004 30º53.3'S 30º24.4'E; MT nr. stream; M. Mostovski |
| NCSU 42246 | MRAC | male | S. AFRICA: KwaZulu- Natal Ramsgate Butterfly Sanctuary; 3-30.x.2004 30º53.3'S 30º24.4'E; MT nr. stream; M. Mostovski |
| NCSU 42247 | PSUC | male | S. AFRICA: KwaZulu- Natal Ramsgate Butterfly Sanctuary; 3-30.x.2004 30º53.3'S 30º24.4'E; MT nr. stream; M. Mostovski |
| NCSU 42248 | PSUC | male | S. AFRICA: KwaZulu- Natal Ramsgate Butterfly Sanctuary; 3-30.x.2004 30º53.3'S 30º24.4'E; MT nr. stream; M. Mostovski |
| NCSU 18846 | PSUC | male | RSA: KwaZulu-Natal; Ramsgate Butterfly Sanctuary: MT nr. stream; -30.888, 30.34; 3.xii.2004-8.i.2005; M. Mostovski |
| NCSU 51866 | PSUC | male | S. AFRICA: Kwa-Zulu Natal: Ramsgate Butterfly Sanctuary;30º53S 30º20E 1.xi-2.xii.2004; MT; near stream; M Mostovski |

*Afrevania leroyi* Benoit, 1953

| **Identifier(s)** | **Repository** | **Sex** | **Collecting event** |
| --- | --- | --- | --- |
| NCSU 53950 | MRAC | female | COLL. MUS. CONGO N. Lac Kivu : Rwankwi 15-VI-1951 J. V. Leroy |
| NCSU 52616 | ISNB | male | Coll. R. I. Sc. N. B. Somalie Mogadiscio Afgoi 1/7-IV-1977 Lower Shabelly valley Malaise Trap L. Bim |
|  | MRAC | female | Nord du lac Kivu: Rwankwi 15-XI-1950 (J. V. Leroy) |

*Trissevania anemotis* Kieffer, 1913

| **Identifier(s)** | **Repository** | **Sex** | **Collecting event** |
| --- | --- | --- | --- |
| NCSU 52051 | MRAC | male | KENYA, Gatamayu Forest, near fishing camp 0°58.68'S, 36°41.62'E 6-13.iii.99, Malaise trap R. Copeland |
| NCSU 53297 | PSUC | male | KENYA, Gatamayu Forest near fishing camp 0°58.68'S, 36°41.62'E 20-27.iii.99, Malaise Trap R. Copeland |
| NCSU 53298 | CAS | male | KENYA, Gatamayu Forest near fishing camp 0°58.68'S, 36°41.62'E 20-27.iii.99, Malaise Trap R. Copeland |
| NCSU 53299 | PSUC | male | KENYA Gatamayu Forest near fishing camp 0°58.68'S, 36°41.62E 2.x.98 R. Copeland |
| NCSU 53505 | NMKE | female | KENYA, Rift Valley Prov., Mt. Elgon Nat. Pk., 1.06995º N, 34.75168º E, 2474m Malaise trap, below Endebess Bluff 16-30APR 2006 R. Copeland, Photo 1174-1183?, E12 |
| NCSU 53503 | NMKE | male | KENYA, Rift Valley Prov., Mt. Elgon Nat. Pk., 1.06995º N, 1.06995º N, 2474m Malaise trap, below Endebess Bluff 11-25 JUN 2006 R. Copeland, Photo 1190-1193 F2 |
| NCSU 53504 | PSUC | female | KENYA, Rift Valley Prov., Mt. Elgon Nat. Pk., 1.06995º N, 34.75168º E, 2474m Malaise trap, below Endebess Bluff 30 APR - 14 MAY 2006 R. Copeland, Photo 1184-1185, F1 |
| NCSU 43254 | NMKE | female | KENYA Eastern Prov. Nyambene Hills Itieni Forest at top, 2507m 0.23417º N, 37.87635ºE Malaise trap Just inside indigenous forest, 22 JUN-6 JUL 2011 R. Copeland |
| NCSU 43255 | NMKE | female | KENYA Eastern Prov. Nyambene Hills Itieni Forest at top, 2507m 0.23417º N, 37.87635ºE Malaise trap Just inside indigenous forest, 25 MAY- 8 JUN 2011 R. Copeland |
| NCSU 43257 | PSUC | female | KENYA Eastern Prov. Nyambene Hills Itieni Forest at top, 2507m 0.23417º N, 37.87635ºE Malaise trap Just inside indigenous forest, 13-27 APR 2011 R. Copeland, 14482-TrissevaniaC1 |
| NCSU 43256 | NMKE | male | KENYA Central Province Castle Forest, southern Mt. Kenya, c. 2100m c. 0.38033ºS, 37.30983ºE Malaise trap nr. Waterfall, just up from lodge, 31 OCT-13 NOV 2008, R. Copeland, 13742-TrissevaniaB5 |
| IM 5194 | PSUC | male | CAMEROON S.W.P. Ball Nyonga 7 Oct I04J.L.Vomia atLiteLegJFCornell |
| PSUC_FEM 000079774 | PSUC | female | KENYA Eastern Prov. Nyambene Hills Itieni Forest at bottom, 2142m 0.24433º N, 37.87016ºE Malaise trap Indigenous forest near forest station 10-24 JUL 2011, R. Copeland |
| PSUC_FEM 000079773 | PSUC | male | KENYA Eastern Prov. Nyambene Hills Itieni Forest at bottom, 2142m 0.24433º N, 37.87016ºE Malaise trap Indigenous forest near forest station 25 JUL - 7 AUG 2011, R. Copeland |
| PSUC_FEM 000079772 | NMKE | female | KENYA Eastern Prov. Nyambene Hills Itieni Forest at bottom, 2142m 0.24433º N, 37.87016ºE Malaise trap Indigenous forest near forest station 15-27 NOV 2011, R. Copeland |
| PSUC_FEM 000079770 | NMKE | female | KENYA Eastern Prov. Nyambene Hills Itieni Forest at top, 2507m 0.23417º N, 37.87635ºE Malaise trap Indigenous forest near forest station 13-27 APR 2011, R. Copeland |

*Trissevania heatherae* sp. nov.

| Identifier(s) | Repository | Sex | Collecting event |
| --- | --- | --- | --- |
| PSUC_FEM 000079769 | NMKE | male | KENYA, Coast Prov. Mrima Hill Forest 4.48576S, 39.25845E 212m Malaise trap, indigenous forest edge 8-22 Aug 2011 R. Copeland |
| PSUC_FEM 000079768 | NMKE | male | KENYA, Coast Prov. Mrima Hill Forest 4.48576S, 39.25845E 212m Malaise trap, indigenous forest edge 8-22 Aug 2011 R. Copeland |
| PSUC_FEM 000079767 | PSUC | male | KENYA, Coast Prov. Mrima Hill Forest 4.48576S, 39.25845E 212m Malaise trap, indigenous forest edge 3-17 Oct 2011 R. Copeland |
| PSUC_FEM 000079766 | PSUC | male | KENYA, Coast Prov. Mrima Hill Forest 4.48576S, 39.25845E 212m Malaise trap, indigenous forest edge 8-22 Aug 2011 R. Copeland |

*Trissevania hugoi* sp. nov.

| Identifier(s) | Repository | Sex | Collecting event |
| --- | --- | --- | --- |
| NCSU 53509 | NMKE | female | BURUNDI, Ruvubu NP, 1382m, 2.98144ºS, 30.45531ºE, Malaise trap 5-21 JAN 2010 R Copeland, Photo 1186-1189, E11 |
| NCSU 0043252 | PSUC | male | BURUNDI, Bururi National Forest, 1955m, 3.93022ºS, 29.61697ºE, Malaise Trap Indigenous forest, near stream 2-16 NOV 2010, R. Copeland, 14482-TrissevaniaB8 |
| NCSU 0043283 | NMKE | male | BURUNDI, Bururi National Forest, 1955m, 3.93022ºS, 29.61697ºE, Malaise Trap Indigenous forest, near stream 19 OCT-2 NOV 2010, R. Copeland |
| PSUC_FEM 79771 | NMKE | male | BURUNDI, Bururi National Forest, 1955m, 3.93022ºS, 29.61697ºE, Malaise Trap Indigenous forest, near stream 27 AUG-07 SEP 2010, R. Copeland |

*Trissevania mrimaensis* sp. nov.

| Identifier(s) | Repository | Sex | Collecting event |
| --- | --- | --- | --- |
| NCSU 43253 | NMKE | female | KENYA, Coast Prov. Mrima Hill Forest 4.48576S, 39.25845E 212m Malaise trap, indigenous forest edge 11-25 July 2011 R. Copeland 14482-TrissevaniaB12 |
| PSUC_FEM 000079765 | NMKE | male | KENYA, Coast Prov. Mrima Hill Forest4.48576S, 39.25845E212m Malaise trap, indigenous forest edge 17-30. Oct 2011R. Copeland |

*Trissevania slideri* sp. nov.

| Identifier(s) | Repository | Sex | Collecting event |
| --- | --- | --- | --- |
| NCSU 53507 | NMKE | male | BURUNDI, Rusizi Nat. Pk. Degraded bush/grassland 774m, 3.34364º S, 29.27246º E Malaise trap, near 3 small trees 5-19 DEC 2009 R. Copeland |
| NCSU 53508 | NMKE | male | BURUNDI, Rusizi Nat. Pk. Degraded bush/grassland 774m, 3.34364º S, 29.27246º E Malaise trap, near 3 small trees 5-19 DEC 2009 R. Copeland |
| NCSU 53506 | PSUC | female | BURUNDI, Rusizi Nat. Pk. Degraded bush/grassland 774m, 3.34364º S, 29.27246º E Malaise trap, near 3 small trees 5-19 DEC 2009 R. Copeland |
