# Supplementary material for: Folding Wings like a Cockroach: A Review of Transverse Wing Folding Ensign Wasps (Hymenoptera: Evaniidae: Afrevania and Trissevania)
Source: PLoS One. 2014 May 2;9(5):e94056. doi: 10.1371/journal.pone.0094056 (PMC4008374; doi:10.1371/journal.pone.0094056)
Supplement: Table S2 — List of abbreviations of anatomical structures applied on Figures. (DOCX) [file pone.0094056.s003.docx]

**Table S2.** List of abbreviations of anatomical structures applied on Figures.

1A2 - first anal vein

1ax2 - fore wing first axillary

2ax2 - fore wing second axillary

2ax3 - fore wing third axillary

amp - anterolateral mesopectal projection

afl – anal-marginal fold line

anf - anterodistal notch of the fore wing

arf – anterior radial flexion line

atp - anterior tentorial pit

ba2 - mesobasalare

c - costal vein

cds - carina delimiting ventrally anterior region of prespecular sulcus

cfl – claval flexion line

dap - dorsal area of the metapectal-propodeal complex

dfl – discal fold line

dsp - dorsolateral setal patch of the metapectal-propodeal complex

ED - epistomal distance

EH - eye height

gsr - gastral scrobe

hcx2 - fore wing humeral complex

HW - head width

IOS - interocellar space

LOL - lateral ocellar length

lvp - lateroventral carina of petiole

M2 - medial vein

mcn - mandibular condyle

mcn - mandibular condyle

MD - malar distance

mehi – mesosoma height

mfl – median flexion line

mpc - mesopleural carina

MPL - dorsal area of the metapectal-propodeal complex median length

mpl2 - fore wing median plate

mps - metapleural sulcus

MSL - mesoscutellum median length

OOL - ocello-ocular length

pfl - prestigmal flexion line

pnf - posterodistal notch of the fore wing

prf – posterior radial fold line

psm - profemoral scrobe of the mesopectus

psr - petiolar scrobe

pss - prespecular suclus

ptn - posterior tooth of the mandible

R2 - radial vein

ret - retinaculum

Sc2 - subcostal vein

sca - scrobal carina of the anteromesoscutum

sfl - poststigmal fold line

spec - speculum

spt - setiferous patches on dorsal region of abdominal terga
