# Supplementary material for: Folding Wings like a Cockroach: A Review of Transverse Wing Folding Ensign Wasps (Hymenoptera: Evaniidae: Afrevania and Trissevania)
Source: PLoS One. 2014 May 2;9(5):e94056. doi: 10.1371/journal.pone.0094056 (PMC4008374; doi:10.1371/journal.pone.0094056)
Supplement: Table S3 — Natural language representations of phenotypes in EQ format (Entity attribute: value) with comments. (DOCX) [file pone.0094056.s004.docx]

**Table S3.** Natural language representations of phenotypes in EQ format (Entity attribute: value) with comments.

1. Anal-marginal fold line count
     0: absent
     1: present
   The presence of the anal-marginal fold line is a possible synapomorphy of Trissevaniini.
2. Antenna color female
     0: scape, pedicel, flagellomeres 1–3 yellow, flagellomeres 4, 5 light brown, flagellomeres 6–11 brown,
     1: dark brown
     2: brown
3. Antenna pilosity
     0: thin, decumbent, brown
     1: thick, semierect, white
4. Antennal shelf count
     0: present
     1: absent
   These phenotypes are from Balhoff et al. 2013. The antennal shelf is absent in all Trissevaniini.
5. Anterior tentorial pit count
     0: present
   The anterior tentorial pit is present in *Brachygaster* and Trissevaniini.
6. Anterior tentorial pit shape
     0: elongate
     1: point-like
   The anterior site of origin of the tentorium and the corresponding anterior tentorial pit are dorsoventrally elongated in *Trissevania* and *Brachygaster*.
7. Anterodistal notch of the fore wing count
     0: absent
     1: present
   The presence of the anterodistal notch of the fore wing is a possible synapomorphy for Trissevaniini.
8. Anterolateral mesopectal projection 2-d shape
     0: isosceles triangular
     1: scalene triangular
     2: square shape
   The semantic representations of the original phenotypes of Balhoff et al. 2013 have been modified for more accuracy (Table S4). The anterolateral mesopectal projection is square shaped in Trissevaniini.
9. Anterolateral mesopectal projection count
     0: absent
     1: present
   These phenotypes is from Balhoff et al. 2013. The anterolateral mesopectal projection is present in all Trissevaniini.
10. Anteromesoscutum sculpture
      1: foveate
    The anteromesoscutum is foveate on all Trissevaniini.
11. Areas in between depressions on median foveate region of anteromesoscutum sculpture
      1: crenulate
    The areas in between depressions of median foveate region of anteromesoscutum is crenulate in Trissevaniini.
12. Body length
      0: 1.8–1.9 mm
      1: 2.0–2.3 mm
      2: 2.6–2.7 mm
      3: 2.4–2.6 mm
      4: 2.4–3.0 mm
13. Carina delimiting ventrally anterior region of prespecular sulcus count
      0: absent
      1: present
    The carina delimiting ventrally the anterior region of prespecular sulcus is present in Trissevaniini.
14. Carinae laterally on frons count
      0: present
      1: absent
    These phenotypes are from Balhoff et al. 2013. Carinae laterally on frons are absent in Trissevaniini.
15. Carinae on gena parallel with posterior margin of compound eye count
      0: present
      1: absent
    Both the natural language and semantic representations of original phenotypes of Mullins et al. 2012: “Carinae on gena presence” have been modified for more accuracy (Table S4). Carinae on gena parallel with posterior margin of compound eye are absent from Trissevaniini.
16. Cranial scrobe of anteromesoscutum count
      0: absent
      1: present
    The cranial scrobe of anteromesoscutum is present in *Brachygaster* and Trissevaniini.
17. Cranium color
      0: black
      1: dark brown
    Both the natural language and semantic representations of original phenotypes of Mullins et al. 2012: “Head color” have been modified for more accuracy.
18. Depression diameter on foveate region of median area of the anteromesoscutum vs. depression diameter on foveate region of lateral area of the anteromesoscutum
      0: depressions larger on median area of the anteromesocutum than on lateral area of the anteromesoscutum
    The depressions on the foveate region of mesoscutum are larger medially than laterally in Trissevaniini. The border between the areas of larger and smaller depressions corresponds to the site of attachment of the dorsoventral and dorsolongitudinal indirect flight muscles and hence mark the notauli.
19. Discal fold line count
      0: absent
      1: present
    The presence of the discal fold line is a possible synapomorphy of Trissevaniini.
20. Distance between depressions vs. diameter of depressions on foveate region of anteromesoscutum.
      0: greater than the diameter of one depression
      1: less than the diameter of one depression
    Both the natural language and semantic representations of original phenotypes of Mullins et al. 2012: “Distance between depressions vs diameter of depressions on mesoscutum” have been modified for more accuracy (Table S4). Diameter of depressions are longer than distances between depressions of foveate region of anteromesoscutum in Trissevaniini.
21. Dorsal area of the metapectal-propodeal complex median length vs. mesoscutellum median length
      0: mesoscutellum 1.0–1.2 x as long as metapectal-propodeal complex
      1: mesoscutellum 1.6–1.8 x as long as metapectal-propodeal complex
      2: mesoscutellum 1.4–1.5 x as long as metapectal-propodeal complex
      3: mesoscutellum 1.5–1.6 x as long as metapectal-propodeal complex
22. Dorsal area of the metapectal-propodeal complex sculpture
      0: areolate
      1: foveate
      2: smooth
    The semantic representations of original phenotypes of Mullins et al. 2012 have been modified for more accuracy (Table S4). The dorsal area of the metapectal propodeal complex is foveate in Trissevaniini.
23. Dorsal margin of mesosoma lateral view shape
      0: straight
      1: convex
    The dorsal region of the mesosoma is flat in *Trissevania mrimaensis* (dorsal margin is straight in lateral view) whereas it is convex in other Trissevaniini species.
24. Dorsolateral setal patch of the metapectal-propodeal complex count
      0: present
      1: absent
    Paired, dorsolateral setal patch is present laterally of the propodeal foramen in Trissevaniini. The setae of the patch are oriented posteromedially and are inserted into the retinaculum when the fore wing is in flexed position.
25. Epistomal distance vs. clypeo-compound eye distance
      0: epistomal distance is shorter than clypeo-compound eye distance
    The epistomal distance is shorter than the clypeo-compound eye distance in Trissevaniiini and *Brachygaster*.
26. Facial striae count
      0: absent
      1: present
    The facial striae are present in *Trissevania anemotis* whereas they are absent from other Trissevaniini.
27. Female flagellum ventral sensillar patch spatial arrangement
      0: F4–F11
      1: F6–F11
      2: F5–F11
    These phenotypes are from Balhoff et al. 2013. Ventral sensillar patches are present on F6–F11 in female Trissevaniini.
28. Female metatibial spines count
      0: present
      1: absent
    Semantic description of original phenotypes of Balhoff et al. 2013: “Female metatibial spines presence” has been modified for more accuracy (Table S4).
29. Female OOL vs. LOL
      0: OOL 1.0–1.2 x as long as LOL
      1: OOL 1.9–2.1 x as long as LOL
    OOL and LOL are almost equal in *Trissevania mrimaensis* whereas OOL is almost 2.0 x as long as LOL in other Trissevaniini taxa.
30. Female petiole length vs. petiole width
      0: 3.9–4.4 x as long as wide
      1: 2.2–3.1 x as long as wide
    The female petiole is more elongate in *Afrevania longipetiolata* than in other Trissevaniini.
31. Female scape length vs. compound eye height
      0: equal to eye height
      1: greater than eye height
      2: eye height is at least 2x as long as scape length
    The semantic representations of original phenotypes of Balhoff et al. 2013: “Female scape length” has been modified for more accuracy (Table S4).
32. Fore wing 1cu-a structure
      0: tubular
      1: not tubular, marked by dark line Fore wing 1 cu-a is not tubular, marked by a darker line in Trissevaniini.
33. Fore wing 1CUa length vs. width
      0: more than two x as long as wide
      1: shorter than wide
    1CUa of the fore wing is elongated in Trissevaniini whereas it is shorter than wide in *Brachygaster* and numerous other Evaniidae (e.g. *Semaeomyia*).
34. Fore wing 1CUa orientation
      0: oriented posterodistally
      1: oriented anterodistally
    The fore wing 1CUa is oriented posterodistally in Trissevaniini whereas is oriented anterodistally in *Brachygaster* and numerous other Evaniidae (e. g. *Semaeomyia*).
35. Fore wing 1RS count
      0: present
      1: absent
    Fore wing 1RS is absent from Trissevaniini.
36. Fore wing 2A count
      0: present
      1: absent
    Fore wing 2A is absent from Trissevaniini.
37. Fore wing 2R1 count
      0: present
      1: absent
    Fore wing 2R1 is absent from Trissevaniini.
38. Fore wing 2RS count
      0: present
      1: absent
    Fore wing 2RS is absent from Trissevaniini.
39. Fore wing 3CU distal region count
      0: present
      1: absent
    Fore wing 3CU is present distally and absent proximally in Trissevaniini.
40. Fore wing 3M count
      0: melanized
      1: not melanized
    Fore wing 3M is absent from Trissevaniini.
41. Fore wing distal margin in flexed position vs. metasoma distal margin
      0: extending beyond posterior margin of metasoma
    Both the natural language and semantic representations of original phenotypes of Mullins et al. 2013: “Fore wing length” has been modified for more accuracy (Table S4). The fore wing (in unfolded position) extends beyond the posterior margin of the metasoma in Trissevaniini.
42. Fore wing m-cu structure
      0: tubular
      1: not tubular, marked by dark line
    Fore wing m-cu is not tubular but marked by a dark line in Trissevaniini.
43. Fore wing RS+M structure
      0: tubular
      1: not tubular, marked by dark line
    Fore wing RS+M is absent from Trissevaniini.
44. Fore wing vein 3RS structure
      0: tubular  
     1: not tubular, marked by dark line
    Fore wing 3RS is tubular in *Trissevania* and not tubular, but marked by dark line in *Afrevania*.
45. Fore wing vein r-rs structure
      0: tubular
      1: not tubular, marked by dark line
    Fore wing r-rs is tubular in *Trissevania* and not tubular, but marked by dark line in *Afrevania*.
46. Gaster color
      0: dark brown
      1: brown
      2: light brown
    The gaster is lighter in *Afrevania* than in *Trissevania*.
47. Gastral scrobe count
      0: absent
      1: present
    These phenotypes are from Balhoff et al. 2013. The gastral scrobe is present in Trissevaniini.
48. Head width vs. IOS
      0: head 1.8–2.0 x as wide as IOS
      1: head about 1.5 x as wide as IOS
    The compound eyes are seemingly less convex and hence the "head width/IOS" ratio is less in *Afrevania leroyi* than in other Trissevaniini.
49. Head+mesosoma median length vs. mesosoma height
      0: Head+mesosoma median length 0.9–1.1 x as long as mesosoma height
      1: Head+mesosoma median length 1.8 x as long as mesosoma height
    The mesosoma is more elongated in *Trissevania mrimaensis* and *T. heatherae* than in other Trissevaniini. The length/height ratio is expressed in the present manuscript as the "median length of the head+mesosoma in dorsal view"(http://purl.obolibrary.org/obo/HAO_0002275)/"The anatomical line that is parallel with the posterior margin of the metapectal-propodeal complex, is adjacent to the anterior margin of the mesocoxa and extends between the dorsal and ventral margins of the mesosoma" (http://purl.obolibrary.org/obo/HAO_0002276).
50. Lateroventral carina of the petiole count
      0: present
      1: absent
    The semantic description of original phenotypes of Balhoff et al. 2013: “Lateroventral carina of the petiole” has been modified for more accuracy (Table S4). The lateroventral carina of the petiole is absent from Trissevaniini.
51. Malar distance vs. eye height
      0: eye 2x as high as malar distance
      1: eye 1.5 x as high as malar distance
    The malar distance is more elongated in *Trissevania anemotis* than in other Trissevaniini.
52. Male petiole length vs. width
      0: 4.9–5.2x as long as wide
      1: 2.5 x as long as wide
      2: 3.0 x as long as wide
      3: 3.5–4.0 x as long as wide
    Both the male and female petioles are more elongated in *Afrevania longipetiolata* than in other Trissevaniini.
53. Mandible color
      0: black with dark brown mandibular teeth
      1: brown
      2: yellow
    The mandible color is useful for the diagnosis of *Afrevania* species: The mandible is yellow in *Afrevania leroyi* while brown or black with dark brown mandibular teeth in *A. longipetiolata*.
54. Mandibular teeth count
      0: 3
      1: 4
    The natural language name of original phenotypes of Mullins et al. 2012: “Mandibular teeth number” has been modified for more accuracy. Trissevaniini have 3 mandibular teeth.
55. Median clypeal projection count
      0: absent
      1: present
    These phenotypes are from Balhoff et al. 2013. The median clypeal projection is present in Trissevaniini.
56. Median clypeal projection sharpness
      0: pointed
      1: blunt
    These phenotypes are from Balhoff et al. 2013. The median clypeal projection is blunt in Trissevaniini.
57. Median conjunctiva of male abdominal tergum 9 count
      0: present
      1: absent
    Median conjunctiva of male abdominal tergum 9 present in Trissevaniini and *Brachygaster*.
58. Mesopleural carina count
      0: absent
      1: present
    The profemoral and mesofemoral scrobes are delimited by a distinct mesopleural carina in *Trissevania mrimaensis, T. heatherae and T. slideri.* The carina is absent from other Trissevaniini species.
59. Mesoscutal humeral sulcus anterior end vs. preaxilla anterior end
      0: mesoscutal humeral sulcus anterior end is posterior to dorsal margin of preaxilla anterior end
      1: mesoscutal humeral sulcus anterior end is adjacent to dorsal margin of preaxilla anterior end
    Both the natural language and semantic representations of original phenotypes of Balhoff et al. 2012: “Mesoscutal humeral sulcus continuity” have been modified for more accuracy (Table S4). The mesoscutal humeral sulcus reaches the anterior end of the preaxilla in Trissevaniini.
60. Mesoscutellum median length vs. anteromesoscutum median length
      0: mesoscutellum longer than or equals anteromesoscutum
      1: mesoscutellum shorter than anteromesoscutum
    The mesoscutellum is shorter than the anteromesoscutum in dorsal view in *Trissevania mrimaensis* and in *T. heatherae* whereas it is longer in other Trissevaniini.
61. Mesoscutellum posterior margin vs. metapectal-propodeal complex anterior margin medially
      0: Posterior margin of mesoscutellum is not adjacent medially to anterior margin of metapectal-propodeal complex
      1: Posterior margin of mesoscutellum is adjacent medially to anterior margin of metapectal-propodeal complex
    The mesoscutellum posterior margin reaches the anterior margin of the metapectal-propodeal complex in Trissevaniini. In other Evaniidae (similarly to most other Hymenoptera taxa) the posteromedian margin of the mesoscutellum is separated from the anteromedian margin of the metapectal-propodeal complex by the median part of the metanotum.
62. Mesosoma color
      0: black tegula dark brown
      1: black tegula light brown
    Although the mesosoma is black in both Trissevaniini genera, the tegula is light brown in *Afrevania* and dark brown in *Trissevania.*
63. Metanoto-metapectal-propodeal complex conjunctiva count
      0: present (metanotum and metapectal propodeal complex separated)
      1: absent (metanotum and metapectal-propodeal complex fused)
    An independent metanotum is absent from Trissevaniini. Two lateral flap-like areas are separated along the anterolater margin from the metapectal-propodeal complex bearing the pleural wing articular surfaces and receiving the insertion of metapecto-metanotal muscles. Based on these characteristics these flap-like areas are possibly homologous with the lateral parts of the metanotum of other Hymenoptera. No areas, possibly homologous with the median part of the metanotum, are differentiated on the anterior margin of the metapectal-propodeal complex of Trissevaniini.
64. Metapleural sulcus position
      0: vertical
      1: horizontal
    These phenotypes are from Balhoff et al. 2013.
65. Metatibia length vs. metabasitarsus length
      0: equal
      1: metatibia 1.4x to 1.6x as long as metabasitarsus
      2: metatibia 1.2x to 1.4x as long as metabasitarsus
      3: metatibia 2x as long as metabasitarsus
    The semantic description of original phenotypes of Mullins et al. 2012: “Metatibia length vs metabasitarsus length” has been modified for more accuracy (Table S4). The metatibia is 1.4–1.6 X as long as the metabasitarsus in Trissevaniini.
66. Notaulus count
      0: present
      1: absent
    Although it is obscured by the depressions of the foveate anteromesoscutum, the notauli (line that separates the site of attachment of the dorsoventral and dorsolongitudinal indirect flight muscles) is present in Trissevaniini and marked by the border between foveate regions of larger and smaller depressions on the anteromesoscutum.
67. Notaulus shape
      0: sigmoid
      1: falciform
    These phenotypes are from Balhoff et al. 2013. The notaulus is falciform in Trissevaniini.
68. Nucha count
      0: absent
      1: present
    These phenotypes are from Balhoff et al. 2013: The nucha is absent from Trissevaniini.
69. Parapsidal signum count
      0: present
      1: absent
    These phenotypes are from Mullins et al. 2012. The parapsidal signum is present in Trissevaniini.
70. Petiolar scrobe pubescence count
      0: present
      1: absent
    The petiolar scrobe is setaceous in *Afrevania* whereas it is glabrous in *Trissevania*.
71. Petiole color
      0: black
      1: brown
      2: anterior region yellow posterior region brown
    The petiole of *Afrevania longipetiolata* is bicolored, yellow anteriorly and brown posteriorly, while the petiole is black or brown in other Trissevaniini.
72. Petiole pilosity
      0: dense
      1: sparse
    These phenotypes are from Balhoff et al. 2013. The petiole pilosity is dense in Trissevaniini.
73. Petiole texture
      0: smooth
      1: furrowed
      2: foveolate
    Semantic description of original phenotypes of Balhoff et al. 2013 has been modified for more accuracy (Table S4). The petiole is furrowed in Trissevaniini.
74. Posterior tooth of the mandible count
      0: absent
      1: present
    The posterior tooth of the mandible is at the distal end of a carina that proximally delimits a concave area on the inner surface of the mandible (ptm: Fig. 11B). Although we have observed the carina in the majority of evaniid genera, the projection is present only in Trissevaniini and *Brachygaster*.
75. Posterodistal notch of the fore wing count
      0: absent
      1: present
    The presence of the posterodistal notch of the fore wing is a possible synapomorphy of Trissevaniini.
76. Poststigmal fold line count
      0: absent
      1: present
    The presence of the poststigmal fold line is a possible synapomorphy of Trissevaniini.
77. Preorbital carina count
      0: present
      1: absent
    These phenotypes are from Mullins et al. 2012. The preorbital carina is absent from Trissevaniini.
78. Prestigmal flexion line count
      0: absent
      1: present
    The presence of the prestigmal flexion line is a possible synapomorphy of Trissevaniini.
79. Profemoral scrobe of the mesopectus count
      1: absent
      2: present
    The dorsal and lateral surfaces of the pronotum (pronotal areas that lay in the same plane with the lateral and dorsal surfaces of the mesosoma) are reduced in Trissevaniini. As a consequence of the reduction of pronotal areas, scrobes, which accommodate the head and the fore leg when they are pressed against the mesosoma and which are usually on the pronotum in Evaniidae, are located on the mesothorax in Trissevaniini and *Brachygaster*.
80. Pronotal lobe carina count
      0: absent
      1: present
    These phenotypes are from Balhoff et al. 2013. The pronotal lobe carina is absent from Trissevaniini.
81. Proximodistal length of pedicel vs. proximodistal length of first flagellomere in male
     0: pedicel at least as long as first flagellomere
     1: pedicel distinctly shorter than first flagellomere
82. Scrobal carina of the anteromesoscutum
      0: present
      1: absent
    The presence of the scrobal carina and the anterior cranial scrobe of the mesoscutum is shared between Trissevaniini and *Brachygaster*.
83. Scutoscutellar suture sculpture
      0: foveate
      1: not foveate
    Both natural language and semantic representations of original phenotypes from Balhoff et al. 2013 have been modified for more accuracy (Table S4). The scutoscutellar suture is foveate in Trissevaniini.
84. Setiferous patch on dorsal region of abdominal terga 4–7 in female count
      0: present
      1: absent
    These phenotypes are from Balhoff et al. 2013.
85. Speculum count
      0: absent
      1: present
    These phenotypes are from Balhoff et al. 2013. The site of origin of the mesopleuro-mesofurcal muscle is delimited anteroventrally by an oblique carina in some Evaniidae, including Trissevaniini. The carina seemingly supports the anterior margin of the fore wing when it is in flexed, rested position.
86. Subantennal carina count
      0: present
      1: absent
    The subantennal carina is absent from Trissevaniini.
87. Subantennal groove count
      0: absent
      1: present
    The subantennal groove is present in Trissevaniini.
88. Submedian propodeal projection count
      0: absent
      1: present
    The semantic representations of these phenotypes have been modified after Balhoff et al. 2013 (Table S4).
89. Transmetapectal line count
      0: present
      1: absent
    The absence of the transmetapectal line is a possible synapomorphy of Trissevaniini.
90. Upper face sculpture
      0: punctate and foveate
      1: foveate
      2: smooth
    The semantic representations of these phenotypes have been modified after Mullins et al. 2012 (Table S4).
91. Ventral margin of mesopectus length
      0: longer than ventral margin of metapectus length
      1: shorter than ventral margin of metapectus length
    The semantic representations of original phenotypes from Balhoff et al. 2013 have been modified for more accuracy (Table S4).
92. Ventro-lateral region of mesosoma texture
      0: foveate
      1: areolate
    These phenotypes are from Balhoff et al. 2013.
93. Vertex sculpture
      0: foveate
      1: smooth
    These phenotypes are from Mullins et al. 2012.
