# Supplementary material for: Folding Wings like a Cockroach: A Review of Transverse Wing Folding Ensign Wasps (Hymenoptera: Evaniidae: Afrevania and Trissevania)
Source: PLoS One. 2014 May 2;9(5):e94056. doi: 10.1371/journal.pone.0094056 (PMC4008374; doi:10.1371/journal.pone.0094056)
Supplement: Table S4 — Semantic representations of phenotypes in Manchester syntax format from different taxonomic treatments of Evaniidae (Mullins et al. 2012, Balhoff et al. 2013 and the present taxonomic treatment). (DOCX) [file pone.0094056.s005.docx]

**Table S4.** Semantic representations of phenotypes in Manchester syntax format from different taxonomic treatments of Evaniidae (Mullins et al. 2012, Balhoff et al. 2013 and the present taxonomic treatment).

| Natural language representation | Present paper | Balhoff et al. 2013 | Mullins et al. 2012 |
| --- | --- | --- | --- |
| Anal-marginal fold line count: absent | not ([phenotype_23439](http://hymao.org/miko2014/phenotypes.owl#phenotype_23439)) |  |  |
| Anal-marginal fold line count: present | [has part](http://purl.obolibrary.org/obo/BFO_0000051) some [wing crease](http://purl.obolibrary.org/obo/HAO_0001092) |  |  |
| Antenna color female: brown | [has part](http://purl.obolibrary.org/obo/BFO_0000051) some ([antenna](http://purl.obolibrary.org/obo/HAO_0000101) and ([bearer of](http://purl.obolibrary.org/obo/RO_0000053) some [brown](http://purl.obolibrary.org/obo/PATO_0000952))) |  |  |
| Antenna color female: dark brown | [has part](http://purl.obolibrary.org/obo/BFO_0000051) some ([antenna](http://purl.obolibrary.org/obo/HAO_0000101) and ([bearer of](http://purl.obolibrary.org/obo/RO_0000053) some [dark brown](http://purl.obolibrary.org/obo/PATO_0001245))) |  |  |
| Antenna color female: scape, pedicel, flagellomeres 1-3 yellow, flagellomeres 4, 5 light brown, flagellomeres 6-11 brown | ([has part](http://purl.obolibrary.org/obo/BFO_0000051) some ([scape](http://purl.obolibrary.org/obo/HAO_0000908) and ([bearer of](http://purl.obolibrary.org/obo/RO_0000053) some [yellow](http://purl.obolibrary.org/obo/PATO_0000324)))) and ([has part](http://purl.obolibrary.org/obo/BFO_0000051) some ([first flagellomere](http://purl.obolibrary.org/obo/HAO_0001148) and ([bearer of](http://purl.obolibrary.org/obo/RO_0000053) some [yellow](http://purl.obolibrary.org/obo/PATO_0000324)))) and ([has part](http://purl.obolibrary.org/obo/BFO_0000051) some ([second flagellomere](http://purl.obolibrary.org/obo/HAO_0001883) and ([bearer of](http://purl.obolibrary.org/obo/RO_0000053) some [yellow](http://purl.obolibrary.org/obo/PATO_0000324)))) and ([has part](http://purl.obolibrary.org/obo/BFO_0000051) some ([tenth flagellomere](http://purl.obolibrary.org/obo/HAO_0001884) and ([bearer of](http://purl.obolibrary.org/obo/RO_0000053) some [brown](http://purl.obolibrary.org/obo/PATO_0000952)))) and ([has part](http://purl.obolibrary.org/obo/BFO_0000051) some ([fifth flagellomere](http://purl.obolibrary.org/obo/HAO_0001885) and ([bearer of](http://purl.obolibrary.org/obo/RO_0000053) some [light brown](http://purl.obolibrary.org/obo/PATO_0001246)))) and ([has part](http://purl.obolibrary.org/obo/BFO_0000051) some ([third flagellomere](http://purl.obolibrary.org/obo/HAO_0001895) and ([bearer of](http://purl.obolibrary.org/obo/RO_0000053) some [yellow](http://purl.obolibrary.org/obo/PATO_0000324)))) and ([has part](http://purl.obolibrary.org/obo/BFO_0000051) some ([fourth flagellomere](http://purl.obolibrary.org/obo/HAO_0001896) and ([bearer of](http://purl.obolibrary.org/obo/RO_0000053) some [light brown](http://purl.obolibrary.org/obo/PATO_0001246)))) and ([has part](http://purl.obolibrary.org/obo/BFO_0000051) some ([sixth flagellomere](http://purl.obolibrary.org/obo/HAO_0001897) and ([bearer of](http://purl.obolibrary.org/obo/RO_0000053) some [brown](http://purl.obolibrary.org/obo/PATO_0000952)))) and ([has part](http://purl.obolibrary.org/obo/BFO_0000051) some ([seventh flagellomere](http://purl.obolibrary.org/obo/HAO_0001898) and ([bearer of](http://purl.obolibrary.org/obo/RO_0000053) some [brown](http://purl.obolibrary.org/obo/PATO_0000952)))) and ([has part](http://purl.obolibrary.org/obo/BFO_0000051) some ([eighth flagellomere](http://purl.obolibrary.org/obo/HAO_0001899) and ([bearer of](http://purl.obolibrary.org/obo/RO_0000053) some [brown](http://purl.obolibrary.org/obo/PATO_0000952)))) and ([has part](http://purl.obolibrary.org/obo/BFO_0000051) some ([ninth flagellomere](http://purl.obolibrary.org/obo/HAO_0001900) and ([bearer of](http://purl.obolibrary.org/obo/RO_0000053) some [brown](http://purl.obolibrary.org/obo/PATO_0000952)))) and ([has part](http://purl.obolibrary.org/obo/BFO_0000051) some ([eleventh flagellomere](http://purl.obolibrary.org/obo/HAO_0001901) and ([bearer of](http://purl.obolibrary.org/obo/RO_0000053) some [brown](http://purl.obolibrary.org/obo/PATO_0000952)))) |  |  |
| Antenna pilosity: thick, semierect, whitish | [has part](http://purl.obolibrary.org/obo/BFO_0000051) some ([antenna](http://purl.obolibrary.org/obo/HAO_0000101) and ([has part](http://purl.obolibrary.org/obo/BFO_0000051) some ([pilosity](http://purl.obolibrary.org/obo/HAO_0001990) and (([bearer of](http://purl.obolibrary.org/obo/RO_0000053) some [white](http://purl.obolibrary.org/obo/PATO_0000323)) and ([bearer of](http://purl.obolibrary.org/obo/RO_0000053) some [semi erect](http://purl.obolibrary.org/obo/PATO_0002260)))))) |  |  |
| Antenna pilosity: thin, decumbent, brownish | [has part](http://purl.obolibrary.org/obo/BFO_0000051) some ([antenna](http://purl.obolibrary.org/obo/HAO_0000101) and ([has part](http://purl.obolibrary.org/obo/BFO_0000051) some ([pilosity](http://purl.obolibrary.org/obo/HAO_0001990) and (([bearer of](http://purl.obolibrary.org/obo/RO_0000053) some [brown](http://purl.obolibrary.org/obo/PATO_0000952)) and ([bearer of](http://purl.obolibrary.org/obo/RO_0000053) some [decumbent](http://purl.obolibrary.org/obo/PATO_0002343)))))) |  |  |
| Antennal shelf count: absent | not ([has part](http://purl.obolibrary.org/obo/BFO_0000051) some [antennal shelf](http://purl.obolibrary.org/obo/HAO_0000105)) | not ([has part](http://purl.obolibrary.org/obo/BFO_0000051) some [antennal shelf](http://purl.obolibrary.org/obo/HAO_0000105)) |  |
| Antennal shelf count: present | [has part](http://purl.obolibrary.org/obo/BFO_0000051) some [antennal shelf](http://purl.obolibrary.org/obo/HAO_0000105) | [has part](http://purl.obolibrary.org/obo/BFO_0000051) some [antennal shelf](http://purl.obolibrary.org/obo/HAO_0000105) |  |
| Anterior tentorial pit count: present | [has part](http://purl.obolibrary.org/obo/BFO_0000051) some [anterior tentorial pit](http://purl.obolibrary.org/obo/HAO_0000126) |  |  |
| Anterior tentorial pit shape: elongate | [has part](http://purl.obolibrary.org/obo/BFO_0000051) some ([anterior tentorial pit](http://purl.obolibrary.org/obo/HAO_0000126) and ([bearer of](http://purl.obolibrary.org/obo/RO_0000053) some [elongated](http://purl.obolibrary.org/obo/PATO_0001154))) |  |  |
| Anterior tentorial pit shape: point-like | [has part](http://purl.obolibrary.org/obo/BFO_0000051) some ([anterior tentorial pit](http://purl.obolibrary.org/obo/HAO_0000126) and ([bearer of](http://purl.obolibrary.org/obo/RO_0000053) some [punctiform](http://purl.obolibrary.org/obo/PATO_0001366))) |  |  |
| Anterodistal notch of the fore wing count: absent | not ([has part](http://purl.obolibrary.org/obo/BFO_0000051) some [anterodistal notch of the fore wing](http://purl.obolibrary.org/obo/HAO_0002260)) |  |  |
| Anterodistal notch of the fore wing count: present | [has part](http://purl.obolibrary.org/obo/BFO_0000051) some [anterodistal notch of the fore wing](http://purl.obolibrary.org/obo/HAO_0002260) |  |  |
| Anterolateral mesopectal projection 2-d shape: isosceles triangular | [has part](http://purl.obolibrary.org/obo/BFO_0000051) some ([anterolateral mesopectal projection](http://purl.obolibrary.org/obo/HAO_0002264) and ([has part](http://purl.obolibrary.org/obo/BFO_0000051) some ([lateral side](http://purl.obolibrary.org/obo/BSPO_0000066) and ([bearer of](http://purl.obolibrary.org/obo/RO_0000053) some [isosceles triangular](http://purl.obolibrary.org/obo/PATO_0002307))))) | [has part](http://purl.obolibrary.org/obo/BFO_0000051) some ([mesopectus](http://purl.obolibrary.org/obo/HAO_0000557) and ([has part](http://purl.obolibrary.org/obo/BFO_0000051) some ([corner](http://purl.obolibrary.org/obo/HAO_0000223) and ([is bearer of](http://purl.obolibrary.org/obo/BFO_0000053) some [isosceles triangular](http://purl.obolibrary.org/obo/PATO_0002307))))) |  |
| Anterolateral mesopectal projection 2-d shape: scalene triangular | [has part](http://purl.obolibrary.org/obo/BFO_0000051) some ([anterolateral mesopectal projection](http://purl.obolibrary.org/obo/HAO_0002264) and ([has part](http://purl.obolibrary.org/obo/BFO_0000051) some ([lateral side](http://purl.obolibrary.org/obo/BSPO_0000066) and ([bearer of](http://purl.obolibrary.org/obo/RO_0000053) some [scalene triangular](http://purl.obolibrary.org/obo/PATO_0002308))))) | [has part](http://purl.obolibrary.org/obo/BFO_0000051) some ([mesopectus](http://purl.obolibrary.org/obo/HAO_0000557) and ([has part](http://purl.obolibrary.org/obo/BFO_0000051) some ([corner](http://purl.obolibrary.org/obo/HAO_0000223) and ([is bearer of](http://purl.obolibrary.org/obo/BFO_0000053) some [scalene triangular](http://purl.obolibrary.org/obo/PATO_0002308))))) |  |
| Anterolateral mesopectal projection 2-d shape: square shape | [has part](http://purl.obolibrary.org/obo/BFO_0000051) some ([anterolateral mesopectal projection](http://purl.obolibrary.org/obo/HAO_0002264) and ([has part](http://purl.obolibrary.org/obo/BFO_0000051) some ([lateral side](http://purl.obolibrary.org/obo/BSPO_0000066) and ([bearer of](http://purl.obolibrary.org/obo/RO_0000053) some [square](http://purl.obolibrary.org/obo/PATO_0000413))))) |  |  |
| Anterolateral mesopectal projection count: absent | not ([has part](http://purl.obolibrary.org/obo/BFO_0000051) some [anterolateral mesopectal projection](http://purl.obolibrary.org/obo/HAO_0002264)) |  |  |
| Anterolateral mesopectal projection count: present | [has part](http://purl.obolibrary.org/obo/BFO_0000051) some [anterolateral mesopectal projection](http://purl.obolibrary.org/obo/HAO_0002264) |  |  |
| Anteromesoscutum sculpture: smooth | ([has part](http://purl.obolibrary.org/obo/BFO_0000051) some [anteromesoscutum](http://purl.obolibrary.org/obo/HAO_0001490)) and ([bearer of](http://purl.obolibrary.org/obo/RO_0000053) some [smooth](http://purl.obolibrary.org/obo/PATO_0000701)) |  |  |
| Anteromesoscutum sculpture: foveate | ([has part](http://purl.obolibrary.org/obo/BFO_0000051) some [anteromesoscutum](http://purl.obolibrary.org/obo/HAO_0001490)) and ([bearer of](http://purl.obolibrary.org/obo/RO_0000053) some [foveate](http://purl.obolibrary.org/obo/PATO_0002296)) |  |  |
| Areas in between depressions on foveate region of anteromesoscutum sculpture: smooth | [has part](http://purl.obolibrary.org/obo/BFO_0000051) some ([anteromesoscutum](http://purl.obolibrary.org/obo/HAO_0001490) and ([has part](http://purl.obolibrary.org/obo/BFO_0000051) some ([region](http://purl.obolibrary.org/obo/HAO_0000893) and (([has part](http://purl.obolibrary.org/obo/BFO_0000051) some ((not ([depression](http://purl.obolibrary.org/obo/HAO_0000241))) and ([bearer of](http://purl.obolibrary.org/obo/RO_0000053) some [smooth](http://purl.obolibrary.org/obo/PATO_0000701)))) and ([bearer of](http://purl.obolibrary.org/obo/RO_0000053) some [foveate](http://purl.obolibrary.org/obo/PATO_0002296)))))) |  |  |
| Areas in between depressions on foveate region of anteromesoscutum sculpture: crenulate | [has part](http://purl.obolibrary.org/obo/BFO_0000051) some ([anteromesoscutum](http://purl.obolibrary.org/obo/HAO_0001490) and ([has part](http://purl.obolibrary.org/obo/BFO_0000051) some ([region](http://purl.obolibrary.org/obo/SO_0000001) and (([has part](http://purl.obolibrary.org/obo/BFO_0000051) some ((not ([depression](http://purl.obolibrary.org/obo/HAO_0000241))) and ([bearer of](http://purl.obolibrary.org/obo/RO_0000053) some [ridged](http://purl.obolibrary.org/obo/PATO_0002358)))) and ([bearer of](http://purl.obolibrary.org/obo/RO_0000053) some [foveate](http://purl.obolibrary.org/obo/PATO_0002296)))))) |  |  |
| Body length universal: 4.6–8.0 mm | [has part](http://purl.obolibrary.org/obo/BFO_0000051) some ([body](http://purl.obolibrary.org/obo/HAO_0000182) and ([has part](http://purl.obolibrary.org/obo/BFO_0000051) some [median anatomical line](http://purl.obolibrary.org/obo/HAO_0002272)) and ([bearer of](http://purl.obolibrary.org/obo/RO_0000053) some ([length](http://purl.obolibrary.org/obo/PATO_0000122) and ([is quality measured as](http://purl.obolibrary.org/obo/IAO_0000417) some (([has measurement unit label](http://purl.obolibrary.org/obo/IAO_0000039) value [millimeter](http://purl.obolibrary.org/obo/UO_0000016)) and ([has measurement value](http://purl.obolibrary.org/obo/IAO_0000004) some ([float](http://www.w3.org/2001/XMLSchema#float)[>= 4.6f] and [float](http://www.w3.org/2001/XMLSchema#float)[<= 8.0f]))))))) |  |  |
| Body length universal: 5.3–6.6 mm | [has part](http://purl.obolibrary.org/obo/BFO_0000051) some ([body](http://purl.obolibrary.org/obo/HAO_0000182) and ([has part](http://purl.obolibrary.org/obo/BFO_0000051) some [median anatomical line](http://purl.obolibrary.org/obo/HAO_0002272)) and ([bearer of](http://purl.obolibrary.org/obo/RO_0000053) some ([length](http://purl.obolibrary.org/obo/PATO_0000122) and ([is quality measured as](http://purl.obolibrary.org/obo/IAO_0000417) some (([has measurement unit label](http://purl.obolibrary.org/obo/IAO_0000039) value [millimeter](http://purl.obolibrary.org/obo/UO_0000016)) and ([has measurement value](http://purl.obolibrary.org/obo/IAO_0000004) some ([float](http://www.w3.org/2001/XMLSchema#float)[>= 5.3f] and [float](http://www.w3.org/2001/XMLSchema#float)[<= 6.6f]))))))) |  |  |
| Body length universal: 1.8-1.9 | [has part](http://purl.obolibrary.org/obo/BFO_0000051) some ([body](http://purl.obolibrary.org/obo/HAO_0000182) and ([has part](http://purl.obolibrary.org/obo/BFO_0000051) some [median anatomical line](http://purl.obolibrary.org/obo/HAO_0002272)) and ([bearer of](http://purl.obolibrary.org/obo/RO_0000053) some ([length](http://purl.obolibrary.org/obo/PATO_0000122) and ([is quality measured as](http://purl.obolibrary.org/obo/IAO_0000417) some (([has measurement unit label](http://purl.obolibrary.org/obo/IAO_0000039) value [millimeter](http://purl.obolibrary.org/obo/UO_0000016)) and ([has measurement value](http://purl.obolibrary.org/obo/IAO_0000004) some ([float](http://www.w3.org/2001/XMLSchema#float)[>= 1.8f] and [float](http://www.w3.org/2001/XMLSchema#float)[<= 1.9f]))))))) |  |  |
| Body length universal: 2.4-3.0 | [has part](http://purl.obolibrary.org/obo/BFO_0000051) some ([body](http://purl.obolibrary.org/obo/HAO_0000182) and ([has part](http://purl.obolibrary.org/obo/BFO_0000051) some [median anatomical line](http://purl.obolibrary.org/obo/HAO_0002272)) and ([bearer of](http://purl.obolibrary.org/obo/RO_0000053) some ([length](http://purl.obolibrary.org/obo/PATO_0000122) and ([is quality measured as](http://purl.obolibrary.org/obo/IAO_0000417) some (([has measurement unit label](http://purl.obolibrary.org/obo/IAO_0000039) value [millimeter](http://purl.obolibrary.org/obo/UO_0000016)) and ([has measurement value](http://purl.obolibrary.org/obo/IAO_0000004) some ([float](http://www.w3.org/2001/XMLSchema#float)[>= 2.4f] and [float](http://www.w3.org/2001/XMLSchema#float)[<= 3.0f]))))))) |  |  |
| Body length universal: 2.0-2.3 | [has part](http://purl.obolibrary.org/obo/BFO_0000051) some ([body](http://purl.obolibrary.org/obo/HAO_0000182) and ([has part](http://purl.obolibrary.org/obo/BFO_0000051) some [median anatomical line](http://purl.obolibrary.org/obo/HAO_0002272)) and ([bearer of](http://purl.obolibrary.org/obo/RO_0000053) some ([length](http://purl.obolibrary.org/obo/PATO_0000122) and ([is quality measured as](http://purl.obolibrary.org/obo/IAO_0000417) some (([has measurement unit label](http://purl.obolibrary.org/obo/IAO_0000039) value [millimeter](http://purl.obolibrary.org/obo/UO_0000016)) and ([has measurement value](http://purl.obolibrary.org/obo/IAO_0000004) some ([float](http://www.w3.org/2001/XMLSchema#float)[>= 2.0f] and [float](http://www.w3.org/2001/XMLSchema#float)[<= 2.3f]))))))) |  |  |
| Body length universal: 2.6-2.7 | [Thing](http://www.w3.org/2002/07/owl#Thing) |  |  |
| Body length universal: 2.4-2.6 | [has part](http://purl.obolibrary.org/obo/BFO_0000051) some ([body](http://purl.obolibrary.org/obo/HAO_0000182) and ([has part](http://purl.obolibrary.org/obo/BFO_0000051) some [median anatomical line](http://purl.obolibrary.org/obo/HAO_0002272)) and ([bearer of](http://purl.obolibrary.org/obo/RO_0000053) some ([length](http://purl.obolibrary.org/obo/PATO_0000122) and ([is quality measured as](http://purl.obolibrary.org/obo/IAO_0000417) some (([has measurement unit label](http://purl.obolibrary.org/obo/IAO_0000039) value [millimeter](http://purl.obolibrary.org/obo/UO_0000016)) and ([has measurement value](http://purl.obolibrary.org/obo/IAO_0000004) some ([float](http://www.w3.org/2001/XMLSchema#float)[>= 2.4f] and [float](http://www.w3.org/2001/XMLSchema#float)[<= 2.6f]))))))) |  |  |
| Body length universal: 5.5-9.1 mm | [has part](http://purl.obolibrary.org/obo/BFO_0000051) some ([body](http://purl.obolibrary.org/obo/HAO_0000182) and ([has part](http://purl.obolibrary.org/obo/BFO_0000051) some [median anatomical line](http://purl.obolibrary.org/obo/HAO_0002272)) and ([bearer of](http://purl.obolibrary.org/obo/RO_0000053) some ([length](http://purl.obolibrary.org/obo/PATO_0000122) and ([is quality measured as](http://purl.obolibrary.org/obo/IAO_0000417) some (([has measurement unit label](http://purl.obolibrary.org/obo/IAO_0000039) value [millimeter](http://purl.obolibrary.org/obo/UO_0000016)) and ([has measurement value](http://purl.obolibrary.org/obo/IAO_0000004) some ([float](http://www.w3.org/2001/XMLSchema#float)[>= 5.5f] and [float](http://www.w3.org/2001/XMLSchema#float)[<= 9.1f]))))))) |  |  |
| Body length universal: 7.0-7.8 mm | [has part](http://purl.obolibrary.org/obo/BFO_0000051) some ([body](http://purl.obolibrary.org/obo/HAO_0000182) and ([has part](http://purl.obolibrary.org/obo/BFO_0000051) some [median anatomical line](http://purl.obolibrary.org/obo/HAO_0002272)) and ([bearer of](http://purl.obolibrary.org/obo/RO_0000053) some ([length](http://purl.obolibrary.org/obo/PATO_0000122) and ([is quality measured as](http://purl.obolibrary.org/obo/IAO_0000417) some (([has measurement unit label](http://purl.obolibrary.org/obo/IAO_0000039) value [millimeter](http://purl.obolibrary.org/obo/UO_0000016)) and ([has measurement value](http://purl.obolibrary.org/obo/IAO_0000004) some ([float](http://www.w3.org/2001/XMLSchema#float)[>= 7.0f] and [float](http://www.w3.org/2001/XMLSchema#float)[<= 7.8f]))))))) |  |  |
| Body length universal: 6.5-6.8 mm | [has part](http://purl.obolibrary.org/obo/BFO_0000051) some ([body](http://purl.obolibrary.org/obo/HAO_0000182) and ([has part](http://purl.obolibrary.org/obo/BFO_0000051) some [median anatomical line](http://purl.obolibrary.org/obo/HAO_0002272)) and ([bearer of](http://purl.obolibrary.org/obo/RO_0000053) some ([length](http://purl.obolibrary.org/obo/PATO_0000122) and ([is quality measured as](http://purl.obolibrary.org/obo/IAO_0000417) some (([has measurement unit label](http://purl.obolibrary.org/obo/IAO_0000039) value [millimeter](http://purl.obolibrary.org/obo/UO_0000016)) and ([has measurement value](http://purl.obolibrary.org/obo/IAO_0000004) some ([float](http://www.w3.org/2001/XMLSchema#float)[>= 6.5f] and [float](http://www.w3.org/2001/XMLSchema#float)[<= 6.8f]))))))) |  |  |
| Carina delimiting ventrally anterior region of prespecular sulcus count: absent | [has part](http://purl.obolibrary.org/obo/BFO_0000051) some ([prespecular sulcus](http://purl.obolibrary.org/obo/HAO_0000816) and ([has part](http://purl.obolibrary.org/obo/BFO_0000051) some ([anterior region](http://purl.obolibrary.org/obo/BSPO_0000071) and ([has part](http://purl.obolibrary.org/obo/BFO_0000051) some ([ventral margin](http://purl.obolibrary.org/obo/BSPO_0000684) and (not ([has part](http://purl.obolibrary.org/obo/BFO_0000051) some [carina](http://purl.obolibrary.org/obo/HAO_0000188)))))))) |  |  |
| Carina delimiting ventrally anterior region of prespecular sulcus count: present | [has part](http://purl.obolibrary.org/obo/BFO_0000051) some ([prespecular sulcus](http://purl.obolibrary.org/obo/HAO_0000816) and ([has part](http://purl.obolibrary.org/obo/BFO_0000051) some ([anterior region](http://purl.obolibrary.org/obo/BSPO_0000071) and ([has part](http://purl.obolibrary.org/obo/BFO_0000051) some ([ventral margin](http://purl.obolibrary.org/obo/BSPO_0000684) and ([has part](http://purl.obolibrary.org/obo/BFO_0000051) some [carina](http://purl.obolibrary.org/obo/HAO_0000188))))))) |  |  |
| Carinae laterally on frons count: present | [has part](http://purl.obolibrary.org/obo/BFO_0000051) some ([frons](http://purl.obolibrary.org/obo/HAO_0001523) and ([has part](http://purl.obolibrary.org/obo/BFO_0000051) some ([lateral region](http://purl.obolibrary.org/obo/BSPO_0000082) and ([has part](http://purl.obolibrary.org/obo/BFO_0000051) some [carina](http://purl.obolibrary.org/obo/HAO_0000188))))) | [has part](http://purl.obolibrary.org/obo/BFO_0000051) some ([frons](http://purl.obolibrary.org/obo/HAO_0001523) and ([has part](http://purl.obolibrary.org/obo/BFO_0000051) some ([lateral region](http://purl.obolibrary.org/obo/BSPO_0000082) and ([has part](http://purl.obolibrary.org/obo/BFO_0000051) some [carina](http://purl.obolibrary.org/obo/HAO_0000188))))) |  |
| Carinae laterally on frons count: absent | [has part](http://purl.obolibrary.org/obo/BFO_0000051) some ([frons](http://purl.obolibrary.org/obo/HAO_0001523) and ([has part](http://purl.obolibrary.org/obo/BFO_0000051) some ([lateral region](http://purl.obolibrary.org/obo/BSPO_0000082) and (not ([has part](http://purl.obolibrary.org/obo/BFO_0000051) some [carina](http://purl.obolibrary.org/obo/HAO_0000188)))))) | [has part](http://purl.obolibrary.org/obo/BFO_0000051) some ([frons](http://purl.obolibrary.org/obo/HAO_0001523) and ([has part](http://purl.obolibrary.org/obo/BFO_0000051) some ([lateral region](http://purl.obolibrary.org/obo/BSPO_0000082) and (not ([has part](http://purl.obolibrary.org/obo/BFO_0000051) some [carina](http://purl.obolibrary.org/obo/HAO_0000188)))))) |  |
| Carinae on gena parallel with posterior margin of compound eye count: present | [has part](http://purl.obolibrary.org/obo/BFO_0000051) some ([gena](http://purl.obolibrary.org/obo/HAO_0000371) and ([has part](http://purl.obolibrary.org/obo/BFO_0000051) some ([carina](http://purl.obolibrary.org/obo/HAO_0000188) and ([parallel_to](http://purl.obolibrary.org/obo/bspo#parallel_to) some ([posterior margin](http://purl.obolibrary.org/obo/BSPO_0000672) and ([part of](http://purl.obolibrary.org/obo/BFO_0000050) some [compound eye](http://purl.obolibrary.org/obo/HAO_0000217))))))) |  | [has_part](http://purl.obolibrary.org/obo/BFO_0000051) some ([gena](http://purl.obolibrary.org/obo/HAO_0000371) and ([has_part](http://purl.obolibrary.org/obo/BFO_0000051) some [carina](http://purl.obolibrary.org/obo/HAO_0000188))) |
| Carinae on gena parallel with posterior margin of compound eye count: absent | [has part](http://purl.obolibrary.org/obo/BFO_0000051) some ([gena](http://purl.obolibrary.org/obo/HAO_0000371) and ([has part](http://purl.obolibrary.org/obo/BFO_0000051) some (not ([carina](http://purl.obolibrary.org/obo/HAO_0000188) and ([parallel_to](http://purl.obolibrary.org/obo/bspo#parallel_to) some ([posterior margin](http://purl.obolibrary.org/obo/BSPO_0000672) and ([part of](http://purl.obolibrary.org/obo/BFO_0000050) some [compound eye](http://purl.obolibrary.org/obo/HAO_0000217)))))))) |  | [has_part](http://purl.obolibrary.org/obo/BFO_0000051) some ([gena](http://purl.obolibrary.org/obo/HAO_0000371) and ([has_part](http://purl.obolibrary.org/obo/BFO_0000051) only (not ([carina](http://purl.obolibrary.org/obo/HAO_0000188))))) |
| Cranial scrobe of anteromesoscutum: absent | not ([has part](http://purl.obolibrary.org/obo/BFO_0000051) some [cranial scrobe of mesoscutum](http://purl.obolibrary.org/obo/HAO_0002255)) |  |  |
| Cranial scrobe of anteromesoscutum: present | [has part](http://purl.obolibrary.org/obo/BFO_0000051) some [cranial scrobe of mesoscutum](http://purl.obolibrary.org/obo/HAO_0002255) |  |  |
| Cranium color: dark brown | [has part](http://purl.obolibrary.org/obo/BFO_0000051) some ([cranium](http://purl.obolibrary.org/obo/HAO_0000234) and ([bearer of](http://purl.obolibrary.org/obo/RO_0000053) some [dark brown](http://purl.obolibrary.org/obo/PATO_0001245))) |  |  |
| Cranium color: black | [has part](http://purl.obolibrary.org/obo/BFO_0000051) some ([cranium](http://purl.obolibrary.org/obo/HAO_0000234) and ([bearer of](http://purl.obolibrary.org/obo/RO_0000053) some [black](http://purl.obolibrary.org/obo/PATO_0000317))) |  |  |
| Depression diameter on foveate region of median area of the anteromesoscutum vs. depression diameter on foveate region of lateral area of the anteromesoscutum: depressions larger on median area of the anteromesocutum than on lateral area of the anteromesosocutum | [has part](http://purl.obolibrary.org/obo/BFO_0000051) some ([median area of the mesoscutum](http://purl.obolibrary.org/obo/HAO_0000520) and ([has part](http://purl.obolibrary.org/obo/BFO_0000051) some ([region](http://purl.obolibrary.org/obo/HAO_0000893) and (([has part](http://purl.obolibrary.org/obo/BFO_0000051) some ([depression](http://purl.obolibrary.org/obo/HAO_0000241) and ([bearer of](http://purl.obolibrary.org/obo/RO_0000053) some ([diameter](http://purl.obolibrary.org/obo/PATO_0001334) and ([increased_in_magnitude_relative_to](http://purl.obolibrary.org/obo/pato#increased_in_magnitude_relative_to) some ([diameter](http://purl.obolibrary.org/obo/PATO_0001334) and ([inheres in](http://purl.obolibrary.org/obo/RO_0000052) some ([depression](http://purl.obolibrary.org/obo/HAO_0000241) and ([part of](http://purl.obolibrary.org/obo/BFO_0000050) some ([region](http://purl.obolibrary.org/obo/HAO_0000893) and (([part of](http://purl.obolibrary.org/obo/BFO_0000050) some [lateral area of the mesoscutum](http://purl.obolibrary.org/obo/HAO_0000466)) and ([bearer of](http://purl.obolibrary.org/obo/RO_0000053) some [foveate](http://purl.obolibrary.org/obo/PATO_0002296))))))))))))) and ([bearer of](http://purl.obolibrary.org/obo/RO_0000053) some [foveate](http://purl.obolibrary.org/obo/PATO_0002296)))))) |  |  |
| Discal fold line count: absent | not ([phenotype_23437](http://hymao.org/miko2014/phenotypes.owl#phenotype_23437)) |  |  |
| Discal fold line count: present | [has part](http://purl.obolibrary.org/obo/BFO_0000051) some [wing crease](http://purl.obolibrary.org/obo/HAO_0001092) |  |  |
| Distance between depressions vs. diameter of depressions on foveate region of anteromesoscutum: less than the diameter of one depression | [has part](http://purl.obolibrary.org/obo/BFO_0000051) some ([anteromesoscutum](http://purl.obolibrary.org/obo/HAO_0001490) and ([has part](http://purl.obolibrary.org/obo/BFO_0000051) some ([region](http://purl.obolibrary.org/obo/HAO_0000893) and ([has part](http://purl.obolibrary.org/obo/BFO_0000051) some ((not ([depression](http://purl.obolibrary.org/obo/HAO_0000241))) and ([bearer of](http://purl.obolibrary.org/obo/RO_0000053) some ([length](http://purl.obolibrary.org/obo/PATO_0000122) and ([decreased_in_magnitude_relative_to](http://purl.obolibrary.org/obo/pato#decreased_in_magnitude_relative_to) some ([length](http://purl.obolibrary.org/obo/PATO_0000122) and ([inheres in](http://purl.obolibrary.org/obo/RO_0000052) some ([depression](http://purl.obolibrary.org/obo/HAO_0000241) and ([part of](http://purl.obolibrary.org/obo/BFO_0000050) some ([region](http://purl.obolibrary.org/obo/HAO_0000893) and ([bearer of](http://purl.obolibrary.org/obo/RO_0000053) some ([foveate](http://purl.obolibrary.org/obo/PATO_0002296) and ([part of](http://purl.obolibrary.org/obo/BFO_0000050) some [mesoscutum](http://purl.obolibrary.org/obo/HAO_0000575)))))))))))))) and ([bearer of](http://purl.obolibrary.org/obo/RO_0000053) some [foveate](http://purl.obolibrary.org/obo/PATO_0002296))))) |  | [has_part](http://purl.obolibrary.org/obo/BFO_0000051) some ([interstice](http://purl.obolibrary.org/obo/HAO_0000438) and ([is bearer of](http://purl.obolibrary.org/obo/BFO_0000053) some ([length](http://purl.obolibrary.org/obo/PATO_0000122) and ([decreased_in_magnitude_relative_to](http://purl.obolibrary.org/obo/pato#decreased_in_magnitude_relative_to) some ([length](http://purl.obolibrary.org/obo/PATO_0000122) and ([inheres in](http://purl.obolibrary.org/obo/BFO_0000052) some (depression and ([part_of](http://purl.obolibrary.org/obo/BFO_0000050) some [mesoscutum](http://purl.obolibrary.org/obo/HAO_0000575))))))))) |
| Distance between depressions vs. diameter of depressions on foveate region of anteromesoscutum: greater than the diameter of one depression | [has part](http://purl.obolibrary.org/obo/BFO_0000051) some ([anteromesoscutum](http://purl.obolibrary.org/obo/HAO_0001490) and ([has part](http://purl.obolibrary.org/obo/BFO_0000051) some ([region](http://purl.obolibrary.org/obo/HAO_0000893) and (([has part](http://purl.obolibrary.org/obo/BFO_0000051) some ((not ([depression](http://purl.obolibrary.org/obo/HAO_0000241))) and ([bearer of](http://purl.obolibrary.org/obo/RO_0000053) some ([length](http://purl.obolibrary.org/obo/PATO_0000122) and ([increased_in_magnitude_relative_to](http://purl.obolibrary.org/obo/pato#increased_in_magnitude_relative_to) some ([length](http://purl.obolibrary.org/obo/PATO_0000122) and ([inheres in](http://purl.obolibrary.org/obo/RO_0000052) some ([depression](http://purl.obolibrary.org/obo/HAO_0000241) and ([part of](http://purl.obolibrary.org/obo/BFO_0000050) some ([region](http://purl.obolibrary.org/obo/HAO_0000893) and ([bearer of](http://purl.obolibrary.org/obo/RO_0000053) some ([foveate](http://purl.obolibrary.org/obo/PATO_0002296) and ([part of](http://purl.obolibrary.org/obo/BFO_0000050) some [mesoscutum](http://purl.obolibrary.org/obo/HAO_0000575)))))))))))))) and ([bearer of](http://purl.obolibrary.org/obo/RO_0000053) some [foveate](http://purl.obolibrary.org/obo/PATO_0002296)))))) |  | [has_part](http://purl.obolibrary.org/obo/BFO_0000051) some ([interstice](http://purl.obolibrary.org/obo/HAO_0000438) and ([is bearer of](http://purl.obolibrary.org/obo/BFO_0000053) some ([length](http://purl.obolibrary.org/obo/PATO_0000122) and ([increased_in_magnitude_relative_to](http://purl.obolibrary.org/obo/pato#increased_in_magnitude_relative_to) some ([length](http://purl.obolibrary.org/obo/PATO_0000122) and ([inheres in](http://purl.obolibrary.org/obo/BFO_0000052) some (depressionand ([part_of](http://purl.obolibrary.org/obo/BFO_0000050) some [mesoscutum](http://purl.obolibrary.org/obo/HAO_0000575))))))))) |
| Dorsal area of the metapectal-propodeal complex median length vs. mesoscutellum median length: mesoscutellum 1.5–1.6 times as long as metapectal-propodeal complex | [has part](http://purl.obolibrary.org/obo/BFO_0000051) some ([mesoscutellum](http://purl.obolibrary.org/obo/HAO_0000574) and ([has part](http://purl.obolibrary.org/obo/BFO_0000051) some ([dorsal side](http://purl.obolibrary.org/obo/BSPO_0000063) and (([has part](http://purl.obolibrary.org/obo/BFO_0000051) some [median anatomical line](http://purl.obolibrary.org/obo/HAO_0002272)) and ([bearer of](http://purl.obolibrary.org/obo/RO_0000053) some ([length](http://purl.obolibrary.org/obo/PATO_0000122) and ([is quality measured as](http://purl.obolibrary.org/obo/IAO_0000417) some (([has measurement unit label](http://purl.obolibrary.org/obo/IAO_0000039) some ([length](http://purl.obolibrary.org/obo/PATO_0000122) and ([inheres in](http://purl.obolibrary.org/obo/RO_0000052) some ([median anatomical line](http://purl.obolibrary.org/obo/HAO_0002272) and ([part of](http://purl.obolibrary.org/obo/BFO_0000050) some ([dorsal side](http://purl.obolibrary.org/obo/BSPO_0000063) and ([part of](http://purl.obolibrary.org/obo/BFO_0000050) some [dorsal area of the metapectal-propodeal complex](http://purl.obolibrary.org/obo/HAO_0001982)))))))) and ([has measurement value](http://purl.obolibrary.org/obo/IAO_0000004) some [float](http://www.w3.org/2001/XMLSchema#float)[>= 1.5f , <= 1.6f]))))))))) |  |  |
| Dorsal area of the metapectal-propodeal complex median length vs. mesoscutellum median length: mesoscutellum 1.0–1.2 times as long as metapectal-propodeal complex | [has part](http://purl.obolibrary.org/obo/BFO_0000051) some ([mesoscutellum](http://purl.obolibrary.org/obo/HAO_0000574) and ([has part](http://purl.obolibrary.org/obo/BFO_0000051) some ([dorsal side](http://purl.obolibrary.org/obo/BSPO_0000063) and (([has part](http://purl.obolibrary.org/obo/BFO_0000051) some [median anatomical line](http://purl.obolibrary.org/obo/HAO_0002272)) and ([bearer of](http://purl.obolibrary.org/obo/RO_0000053) some ([length](http://purl.obolibrary.org/obo/PATO_0000122) and ([is quality measured as](http://purl.obolibrary.org/obo/IAO_0000417) some (([has measurement unit label](http://purl.obolibrary.org/obo/IAO_0000039) some ([length](http://purl.obolibrary.org/obo/PATO_0000122) and ([inheres in](http://purl.obolibrary.org/obo/RO_0000052) some ([median anatomical line](http://purl.obolibrary.org/obo/HAO_0002272) and ([part of](http://purl.obolibrary.org/obo/BFO_0000050) some ([dorsal side](http://purl.obolibrary.org/obo/BSPO_0000063) and ([part of](http://purl.obolibrary.org/obo/BFO_0000050) some [dorsal area of the metapectal-propodeal complex](http://purl.obolibrary.org/obo/HAO_0001982)))))))) and ([has measurement value](http://purl.obolibrary.org/obo/IAO_0000004) some [float](http://www.w3.org/2001/XMLSchema#float)[>= 1.0f , <= 1.2f]))))))))) |  |  |
| Dorsal area of the metapectal-propodeal complex median length vs. mesoscutellum median length: mesoscutellum 1.6–1.8 times as long as metapectal-propodeal complex | [has part](http://purl.obolibrary.org/obo/BFO_0000051) some ([mesoscutellum](http://purl.obolibrary.org/obo/HAO_0000574) and ([has part](http://purl.obolibrary.org/obo/BFO_0000051) some ([dorsal side](http://purl.obolibrary.org/obo/BSPO_0000063) and (([has part](http://purl.obolibrary.org/obo/BFO_0000051) some [median anatomical line](http://purl.obolibrary.org/obo/HAO_0002272)) and ([bearer of](http://purl.obolibrary.org/obo/RO_0000053) some ([length](http://purl.obolibrary.org/obo/PATO_0000122) and ([is quality measured as](http://purl.obolibrary.org/obo/IAO_0000417) some (([has measurement unit label](http://purl.obolibrary.org/obo/IAO_0000039) some ([length](http://purl.obolibrary.org/obo/PATO_0000122) and ([inheres in](http://purl.obolibrary.org/obo/RO_0000052) some ([median anatomical line](http://purl.obolibrary.org/obo/HAO_0002272) and ([part of](http://purl.obolibrary.org/obo/BFO_0000050) some ([dorsal side](http://purl.obolibrary.org/obo/BSPO_0000063) and ([part of](http://purl.obolibrary.org/obo/BFO_0000050) some [dorsal area of the metapectal-propodeal complex](http://purl.obolibrary.org/obo/HAO_0001982)))))))) and ([has measurement value](http://purl.obolibrary.org/obo/IAO_0000004) some [float](http://www.w3.org/2001/XMLSchema#float)[>= 1.6f , <= 1.8f]))))))))) |  |  |
| Dorsal area of the metapectal-propodeal complex median length vs. mesoscutellum median length: mesoscutellum 1.4–1.5 times as long as metapectal-propodeal complex | [has part](http://purl.obolibrary.org/obo/BFO_0000051) some ([mesoscutellum](http://purl.obolibrary.org/obo/HAO_0000574) and ([has part](http://purl.obolibrary.org/obo/BFO_0000051) some ([dorsal side](http://purl.obolibrary.org/obo/BSPO_0000063) and (([has part](http://purl.obolibrary.org/obo/BFO_0000051) some [median anatomical line](http://purl.obolibrary.org/obo/HAO_0002272)) and ([bearer of](http://purl.obolibrary.org/obo/RO_0000053) some ([length](http://purl.obolibrary.org/obo/PATO_0000122) and ([is quality measured as](http://purl.obolibrary.org/obo/IAO_0000417) some (([has measurement unit label](http://purl.obolibrary.org/obo/IAO_0000039) some ([length](http://purl.obolibrary.org/obo/PATO_0000122) and ([inheres in](http://purl.obolibrary.org/obo/RO_0000052) some ([median anatomical line](http://purl.obolibrary.org/obo/HAO_0002272) and ([part of](http://purl.obolibrary.org/obo/BFO_0000050) some ([dorsal side](http://purl.obolibrary.org/obo/BSPO_0000063) and ([part of](http://purl.obolibrary.org/obo/BFO_0000050) some [dorsal area of the metapectal-propodeal complex](http://purl.obolibrary.org/obo/HAO_0001982)))))))) and ([has measurement value](http://purl.obolibrary.org/obo/IAO_0000004) some [float](http://www.w3.org/2001/XMLSchema#float)[>= 1.4f , <= 1.5f]))))))))) |  |  |
| Dorsal area of the metapectal-propodeal complex sculpture: areolate | [has part](http://purl.obolibrary.org/obo/BFO_0000051) some ([dorsal area of the metapectal-propodeal complex](http://purl.obolibrary.org/obo/HAO_0001982) and ([bearer of](http://purl.obolibrary.org/obo/RO_0000053) some [areolate](http://purl.obolibrary.org/obo/PATO_0002295))) |  | [has_part](http://purl.obolibrary.org/obo/BFO_0000051) some ([metapectal-propodeal complex](http://purl.obolibrary.org/obo/HAO_0000604) and ([has_part](http://purl.obolibrary.org/obo/BFO_0000051) some ([dorsal side](http://purl.obolibrary.org/obo/BSPO_0000063) and ([is bearer of](http://purl.obolibrary.org/obo/BFO_0000053) some [areolate](http://purl.obolibrary.org/obo/PATO_0002295))))) |
| Dorsal area of the metapectal-propodeal complex sculpture: foveate | [has part](http://purl.obolibrary.org/obo/BFO_0000051) some ([dorsal area of the metapectal-propodeal complex](http://purl.obolibrary.org/obo/HAO_0001982) and ([bearer of](http://purl.obolibrary.org/obo/RO_0000053) some [foveate](http://purl.obolibrary.org/obo/PATO_0002296))) |  | [has_part](http://purl.obolibrary.org/obo/BFO_0000051) some ([metapectal-propodeal complex](http://purl.obolibrary.org/obo/HAO_0000604) and ([has_part](http://purl.obolibrary.org/obo/BFO_0000051) some ([dorsal side](http://purl.obolibrary.org/obo/BSPO_0000063) and ([is bearer of](http://purl.obolibrary.org/obo/BFO_0000053) some [foveate](http://purl.obolibrary.org/obo/PATO_0002296))))) |
| Dorsal area of the metapectal-propodeal complex sculpture: smooth | [has part](http://purl.obolibrary.org/obo/BFO_0000051) some ([dorsal area of the metapectal-propodeal complex](http://purl.obolibrary.org/obo/HAO_0001982) and ([bearer of](http://purl.obolibrary.org/obo/RO_0000053) some [smooth](http://purl.obolibrary.org/obo/PATO_0000701))) |  | [has_part](http://purl.obolibrary.org/obo/BFO_0000051) some ([metapectal-propodeal complex](http://purl.obolibrary.org/obo/HAO_0000604) and ([has_part](http://purl.obolibrary.org/obo/BFO_0000051) some ([dorsal side](http://purl.obolibrary.org/obo/BSPO_0000063) and ([is bearer of](http://purl.obolibrary.org/obo/BFO_0000053) some [smooth](http://purl.obolibrary.org/obo/PATO_0000701))))) |
| Dorsal margin of mesosoma lateral view shape: convex | [has part](http://purl.obolibrary.org/obo/BFO_0000051) some ([mesosoma](http://purl.obolibrary.org/obo/HAO_0000576) and ([has part](http://purl.obolibrary.org/obo/BFO_0000051) some ([lateral side](http://purl.obolibrary.org/obo/BSPO_0000066) and ([has part](http://purl.obolibrary.org/obo/BFO_0000051) some ([dorsal margin](http://purl.obolibrary.org/obo/BSPO_0000679) and ([bearer of](http://purl.obolibrary.org/obo/RO_0000053) some [convex](http://purl.obolibrary.org/obo/PATO_0001355))))))) |  |  |
| Dorsal margin of mesosoma lateral view shape: straight | [has part](http://purl.obolibrary.org/obo/BFO_0000051) some ([mesosoma](http://purl.obolibrary.org/obo/HAO_0000576) and ([has part](http://purl.obolibrary.org/obo/BFO_0000051) some ([lateral side](http://purl.obolibrary.org/obo/BSPO_0000066) and ([has part](http://purl.obolibrary.org/obo/BFO_0000051) some ([dorsal margin](http://purl.obolibrary.org/obo/BSPO_0000679) and ([bearer of](http://purl.obolibrary.org/obo/RO_0000053) some [straight](http://purl.obolibrary.org/obo/PATO_0002180))))))) |  |  |
| Dorsolateral setal patch of the metapectal-propodeal complex count: absent | not ([has part](http://purl.obolibrary.org/obo/BFO_0000051) some [dorsolateral setal patch of propodeum](http://purl.obolibrary.org/obo/HAO_0002259)) |  |  |
| Dorsolateral setal patch of the metapectal-propodeal complex count: present | [has part](http://purl.obolibrary.org/obo/BFO_0000051) some [dorsolateral setal patch of propodeum](http://purl.obolibrary.org/obo/HAO_0002259) |  |  |
| Epistomal distance vs. clypeo-compound eye distance: epistomal distance is shorter than clypeo-compound eye distance | [has part](http://purl.obolibrary.org/obo/BFO_0000051) some ([epistomal distance](http://purl.obolibrary.org/obo/HAO_0002263) and ([bearer of](http://purl.obolibrary.org/obo/RO_0000053) some ([length](http://purl.obolibrary.org/obo/PATO_0000122) and ([decreased_in_magnitude_relative_to](http://purl.obolibrary.org/obo/pato#decreased_in_magnitude_relative_to) some ([length](http://purl.obolibrary.org/obo/PATO_0000122) and ([inheres in](http://purl.obolibrary.org/obo/RO_0000052) some [clypeo-compound eye distance](http://purl.obolibrary.org/obo/HAO_0002253))))))) |  |  |
| Facial striae count: present | [has part](http://purl.obolibrary.org/obo/BFO_0000051) some [periepistomal striation](http://purl.obolibrary.org/obo/HAO_0001770) |  |  |
| Facial striae count: absent | not ([has part](http://purl.obolibrary.org/obo/BFO_0000051) some [periepistomal striation](http://purl.obolibrary.org/obo/HAO_0001770)) |  |  |
| Female flagellum ventral sensillar patch spatial arrangement: F4–F11 | (([has part](http://purl.obolibrary.org/obo/BFO_0000051) some [tenth flagellomere](http://purl.obolibrary.org/obo/HAO_0001884)) and ([has part](http://purl.obolibrary.org/obo/BFO_0000051) some ([ventral side](http://purl.obolibrary.org/obo/BSPO_0000068) and ([has part](http://purl.obolibrary.org/obo/BFO_0000051) some [sensillar patch](http://purl.obolibrary.org/obo/HAO_0000931))))) and (([has part](http://purl.obolibrary.org/obo/BFO_0000051) some [fifth flagellomere](http://purl.obolibrary.org/obo/HAO_0001885)) and ([has part](http://purl.obolibrary.org/obo/BFO_0000051) some ([ventral side](http://purl.obolibrary.org/obo/BSPO_0000068) and ([has part](http://purl.obolibrary.org/obo/BFO_0000051) some [sensillar patch](http://purl.obolibrary.org/obo/HAO_0000931))))) and (([has part](http://purl.obolibrary.org/obo/BFO_0000051) some [fourth flagellomere](http://purl.obolibrary.org/obo/HAO_0001896)) and ([has part](http://purl.obolibrary.org/obo/BFO_0000051) some ([ventral side](http://purl.obolibrary.org/obo/BSPO_0000068) and ([has part](http://purl.obolibrary.org/obo/BFO_0000051) some [sensillar patch](http://purl.obolibrary.org/obo/HAO_0000931))))) and (([has part](http://purl.obolibrary.org/obo/BFO_0000051) some [sixth flagellomere](http://purl.obolibrary.org/obo/HAO_0001897)) and ([has part](http://purl.obolibrary.org/obo/BFO_0000051) some ([ventral side](http://purl.obolibrary.org/obo/BSPO_0000068) and ([has part](http://purl.obolibrary.org/obo/BFO_0000051) some [sensillar patch](http://purl.obolibrary.org/obo/HAO_0000931))))) and (([has part](http://purl.obolibrary.org/obo/BFO_0000051) some [seventh flagellomere](http://purl.obolibrary.org/obo/HAO_0001898)) and ([has part](http://purl.obolibrary.org/obo/BFO_0000051) some ([ventral side](http://purl.obolibrary.org/obo/BSPO_0000068) and ([has part](http://purl.obolibrary.org/obo/BFO_0000051) some [sensillar patch](http://purl.obolibrary.org/obo/HAO_0000931))))) and (([has part](http://purl.obolibrary.org/obo/BFO_0000051) some [eighth flagellomere](http://purl.obolibrary.org/obo/HAO_0001899)) and ([has part](http://purl.obolibrary.org/obo/BFO_0000051) some ([ventral side](http://purl.obolibrary.org/obo/BSPO_0000068) and ([has part](http://purl.obolibrary.org/obo/BFO_0000051) some [sensillar patch](http://purl.obolibrary.org/obo/HAO_0000931))))) and (([has part](http://purl.obolibrary.org/obo/BFO_0000051) some [ninth flagellomere](http://purl.obolibrary.org/obo/HAO_0001900)) and ([has part](http://purl.obolibrary.org/obo/BFO_0000051) some ([ventral side](http://purl.obolibrary.org/obo/BSPO_0000068) and ([has part](http://purl.obolibrary.org/obo/BFO_0000051) some [sensillar patch](http://purl.obolibrary.org/obo/HAO_0000931))))) and (([has part](http://purl.obolibrary.org/obo/BFO_0000051) some [eleventh flagellomere](http://purl.obolibrary.org/obo/HAO_0001901)) and ([has part](http://purl.obolibrary.org/obo/BFO_0000051) some ([ventral side](http://purl.obolibrary.org/obo/BSPO_0000068) and ([has part](http://purl.obolibrary.org/obo/BFO_0000051) some [sensillar patch](http://purl.obolibrary.org/obo/HAO_0000931))))) and ([has part](http://purl.obolibrary.org/obo/BFO_0000051) some ([apical flagellomere](http://purl.obolibrary.org/obo/HAO_0000137) and ([has part](http://purl.obolibrary.org/obo/BFO_0000051) some ([ventral side](http://purl.obolibrary.org/obo/BSPO_0000068) and (not ([has part](http://purl.obolibrary.org/obo/BFO_0000051) some [sensillar patch](http://purl.obolibrary.org/obo/HAO_0000931))))))) and ([has part](http://purl.obolibrary.org/obo/BFO_0000051) some ([first flagellomere](http://purl.obolibrary.org/obo/HAO_0001148) and ([has part](http://purl.obolibrary.org/obo/BFO_0000051) some ([ventral side](http://purl.obolibrary.org/obo/BSPO_0000068) and (not ([has part](http://purl.obolibrary.org/obo/BFO_0000051) some [sensillar patch](http://purl.obolibrary.org/obo/HAO_0000931))))))) and ([has part](http://purl.obolibrary.org/obo/BFO_0000051) some ([second flagellomere](http://purl.obolibrary.org/obo/HAO_0001883) and ([has part](http://purl.obolibrary.org/obo/BFO_0000051) some ([ventral side](http://purl.obolibrary.org/obo/BSPO_0000068) and (not ([has part](http://purl.obolibrary.org/obo/BFO_0000051) some [sensillar patch](http://purl.obolibrary.org/obo/HAO_0000931))))))) and ([has part](http://purl.obolibrary.org/obo/BFO_0000051) some ([third flagellomere](http://purl.obolibrary.org/obo/HAO_0001895) and ([has part](http://purl.obolibrary.org/obo/BFO_0000051) some ([ventral side](http://purl.obolibrary.org/obo/BSPO_0000068) and (not ([has part](http://purl.obolibrary.org/obo/BFO_0000051) some [sensillar patch](http://purl.obolibrary.org/obo/HAO_0000931))))))) | ([has part](http://purl.obolibrary.org/obo/BFO_0000051) some ([apical flagellomere](http://purl.obolibrary.org/obo/HAO_0000137) and [eleventh flagellomere](http://purl.obolibrary.org/obo/HAO_0001901) and ([has part](http://purl.obolibrary.org/obo/BFO_0000051) some ([ventral side](http://purl.obolibrary.org/obo/BSPO_0000068) and ([has part](http://purl.obolibrary.org/obo/BFO_0000051) some [sensillar patch](http://purl.obolibrary.org/obo/HAO_0000931)))))) and ([has part](http://purl.obolibrary.org/obo/BFO_0000051) some ([tenth flagellomere](http://purl.obolibrary.org/obo/HAO_0001884) and ([has part](http://purl.obolibrary.org/obo/BFO_0000051) some ([ventral side](http://purl.obolibrary.org/obo/BSPO_0000068) and ([has part](http://purl.obolibrary.org/obo/BFO_0000051) some [sensillar patch](http://purl.obolibrary.org/obo/HAO_0000931)))))) and ([has part](http://purl.obolibrary.org/obo/BFO_0000051) some ([fifth flagellomere](http://purl.obolibrary.org/obo/HAO_0001885) and ([has part](http://purl.obolibrary.org/obo/BFO_0000051) some ([ventral side](http://purl.obolibrary.org/obo/BSPO_0000068) and ([has part](http://purl.obolibrary.org/obo/BFO_0000051) some [sensillar patch](http://purl.obolibrary.org/obo/HAO_0000931)))))) and ([has part](http://purl.obolibrary.org/obo/BFO_0000051) some ([fourth flagellomere](http://purl.obolibrary.org/obo/HAO_0001896) and ([has part](http://purl.obolibrary.org/obo/BFO_0000051) some ([ventral side](http://purl.obolibrary.org/obo/BSPO_0000068) and ([has part](http://purl.obolibrary.org/obo/BFO_0000051) some [sensillar patch](http://purl.obolibrary.org/obo/HAO_0000931)))))) and ([has part](http://purl.obolibrary.org/obo/BFO_0000051) some ([sixth flagellomere](http://purl.obolibrary.org/obo/HAO_0001897) and ([has part](http://purl.obolibrary.org/obo/BFO_0000051) some ([ventral side](http://purl.obolibrary.org/obo/BSPO_0000068) and ([has part](http://purl.obolibrary.org/obo/BFO_0000051) some [sensillar patch](http://purl.obolibrary.org/obo/HAO_0000931)))))) and ([has part](http://purl.obolibrary.org/obo/BFO_0000051) some ([seventh flagellomere](http://purl.obolibrary.org/obo/HAO_0001898) and ([has part](http://purl.obolibrary.org/obo/BFO_0000051) some ([ventral side](http://purl.obolibrary.org/obo/BSPO_0000068) and ([has part](http://purl.obolibrary.org/obo/BFO_0000051) some [sensillar patch](http://purl.obolibrary.org/obo/HAO_0000931)))))) and ([has part](http://purl.obolibrary.org/obo/BFO_0000051) some ([eighth flagellomere](http://purl.obolibrary.org/obo/HAO_0001899) and ([has part](http://purl.obolibrary.org/obo/BFO_0000051) some ([ventral side](http://purl.obolibrary.org/obo/BSPO_0000068) and ([has part](http://purl.obolibrary.org/obo/BFO_0000051) some [sensillar patch](http://purl.obolibrary.org/obo/HAO_0000931)))))) and ([has part](http://purl.obolibrary.org/obo/BFO_0000051) some ([ninth flagellomere](http://purl.obolibrary.org/obo/HAO_0001900) and ([has part](http://purl.obolibrary.org/obo/BFO_0000051) some ([ventral side](http://purl.obolibrary.org/obo/BSPO_0000068)and ([has part](http://purl.obolibrary.org/obo/BFO_0000051) some [sensillar patch](http://purl.obolibrary.org/obo/HAO_0000931)))))) |  |
| Female flagellum ventral sensillar patch spatial arrangement: F6-F11 | (([has part](http://purl.obolibrary.org/obo/BFO_0000051) some [tenth flagellomere](http://purl.obolibrary.org/obo/HAO_0001884)) and ([has part](http://purl.obolibrary.org/obo/BFO_0000051) some ([ventral side](http://purl.obolibrary.org/obo/BSPO_0000068) and ([has part](http://purl.obolibrary.org/obo/BFO_0000051) some [sensillar patch](http://purl.obolibrary.org/obo/HAO_0000931))))) and (([has part](http://purl.obolibrary.org/obo/BFO_0000051) some [fifth flagellomere](http://purl.obolibrary.org/obo/HAO_0001885)) and ([has part](http://purl.obolibrary.org/obo/BFO_0000051) some ([ventral side](http://purl.obolibrary.org/obo/BSPO_0000068) and (not ([has part](http://purl.obolibrary.org/obo/BFO_0000051) some [sensillar patch](http://purl.obolibrary.org/obo/HAO_0000931)))))) and (([has part](http://purl.obolibrary.org/obo/BFO_0000051) some [fourth flagellomere](http://purl.obolibrary.org/obo/HAO_0001896)) and ([has part](http://purl.obolibrary.org/obo/BFO_0000051) some ([ventral side](http://purl.obolibrary.org/obo/BSPO_0000068) and (not ([has part](http://purl.obolibrary.org/obo/BFO_0000051) some [sensillar patch](http://purl.obolibrary.org/obo/HAO_0000931)))))) and (([has part](http://purl.obolibrary.org/obo/BFO_0000051) some [sixth flagellomere](http://purl.obolibrary.org/obo/HAO_0001897)) and ([has part](http://purl.obolibrary.org/obo/BFO_0000051) some ([ventral side](http://purl.obolibrary.org/obo/BSPO_0000068) and ([has part](http://purl.obolibrary.org/obo/BFO_0000051) some [sensillar patch](http://purl.obolibrary.org/obo/HAO_0000931))))) and (([has part](http://purl.obolibrary.org/obo/BFO_0000051) some [seventh flagellomere](http://purl.obolibrary.org/obo/HAO_0001898)) and ([has part](http://purl.obolibrary.org/obo/BFO_0000051) some ([ventral side](http://purl.obolibrary.org/obo/BSPO_0000068) and ([has part](http://purl.obolibrary.org/obo/BFO_0000051) some [sensillar patch](http://purl.obolibrary.org/obo/HAO_0000931))))) and (([has part](http://purl.obolibrary.org/obo/BFO_0000051) some [eighth flagellomere](http://purl.obolibrary.org/obo/HAO_0001899)) and ([has part](http://purl.obolibrary.org/obo/BFO_0000051) some ([ventral side](http://purl.obolibrary.org/obo/BSPO_0000068) and ([has part](http://purl.obolibrary.org/obo/BFO_0000051) some [sensillar patch](http://purl.obolibrary.org/obo/HAO_0000931))))) and (([has part](http://purl.obolibrary.org/obo/BFO_0000051) some [ninth flagellomere](http://purl.obolibrary.org/obo/HAO_0001900)) and ([has part](http://purl.obolibrary.org/obo/BFO_0000051) some ([ventral side](http://purl.obolibrary.org/obo/BSPO_0000068) and ([has part](http://purl.obolibrary.org/obo/BFO_0000051) some [sensillar patch](http://purl.obolibrary.org/obo/HAO_0000931))))) and (([has part](http://purl.obolibrary.org/obo/BFO_0000051) some [eleventh flagellomere](http://purl.obolibrary.org/obo/HAO_0001901)) and ([has part](http://purl.obolibrary.org/obo/BFO_0000051) some ([ventral side](http://purl.obolibrary.org/obo/BSPO_0000068) and ([has part](http://purl.obolibrary.org/obo/BFO_0000051) some [sensillar patch](http://purl.obolibrary.org/obo/HAO_0000931))))) and ([has part](http://purl.obolibrary.org/obo/BFO_0000051) some ([apical flagellomere](http://purl.obolibrary.org/obo/HAO_0000137) and ([has part](http://purl.obolibrary.org/obo/BFO_0000051) some ([ventral side](http://purl.obolibrary.org/obo/BSPO_0000068) and (not ([has part](http://purl.obolibrary.org/obo/BFO_0000051) some [sensillar patch](http://purl.obolibrary.org/obo/HAO_0000931))))))) and ([has part](http://purl.obolibrary.org/obo/BFO_0000051) some ([first flagellomere](http://purl.obolibrary.org/obo/HAO_0001148) and ([has part](http://purl.obolibrary.org/obo/BFO_0000051) some ([ventral side](http://purl.obolibrary.org/obo/BSPO_0000068) and (not ([has part](http://purl.obolibrary.org/obo/BFO_0000051) some [sensillar patch](http://purl.obolibrary.org/obo/HAO_0000931))))))) and ([has part](http://purl.obolibrary.org/obo/BFO_0000051) some ([second flagellomere](http://purl.obolibrary.org/obo/HAO_0001883) and ([has part](http://purl.obolibrary.org/obo/BFO_0000051) some ([ventral side](http://purl.obolibrary.org/obo/BSPO_0000068) and (not ([has part](http://purl.obolibrary.org/obo/BFO_0000051) some [sensillar patch](http://purl.obolibrary.org/obo/HAO_0000931))))))) and ([has part](http://purl.obolibrary.org/obo/BFO_0000051) some ([third flagellomere](http://purl.obolibrary.org/obo/HAO_0001895) and ([has part](http://purl.obolibrary.org/obo/BFO_0000051) some ([ventral side](http://purl.obolibrary.org/obo/BSPO_0000068) and (not ([has part](http://purl.obolibrary.org/obo/BFO_0000051) some [sensillar patch](http://purl.obolibrary.org/obo/HAO_0000931))))))) | ([has part](http://purl.obolibrary.org/obo/BFO_0000051) some ([apical flagellomere](http://purl.obolibrary.org/obo/HAO_0000137) and [eleventh flagellomere](http://purl.obolibrary.org/obo/HAO_0001901) and ([has part](http://purl.obolibrary.org/obo/BFO_0000051) some ([ventral side](http://purl.obolibrary.org/obo/BSPO_0000068) and ([has part](http://purl.obolibrary.org/obo/BFO_0000051) some [sensillar patch](http://purl.obolibrary.org/obo/HAO_0000931)))))) and ([has part](http://purl.obolibrary.org/obo/BFO_0000051) some ([tenth flagellomere](http://purl.obolibrary.org/obo/HAO_0001884) and ([has part](http://purl.obolibrary.org/obo/BFO_0000051) some ([ventral side](http://purl.obolibrary.org/obo/BSPO_0000068) and ([has part](http://purl.obolibrary.org/obo/BFO_0000051) some [sensillar patch](http://purl.obolibrary.org/obo/HAO_0000931)))))) and ([has part](http://purl.obolibrary.org/obo/BFO_0000051) some ([fifth flagellomere](http://purl.obolibrary.org/obo/HAO_0001885) and ([has part](http://purl.obolibrary.org/obo/BFO_0000051) some ([ventral side](http://purl.obolibrary.org/obo/BSPO_0000068) and (not ([has part](http://purl.obolibrary.org/obo/BFO_0000051) some [sensillar patch](http://purl.obolibrary.org/obo/HAO_0000931))))))) and ([has part](http://purl.obolibrary.org/obo/BFO_0000051) some ([fourth flagellomere](http://purl.obolibrary.org/obo/HAO_0001896) and ([has part](http://purl.obolibrary.org/obo/BFO_0000051) some ([ventral side](http://purl.obolibrary.org/obo/BSPO_0000068) and (not ([has part](http://purl.obolibrary.org/obo/BFO_0000051) some [sensillar patch](http://purl.obolibrary.org/obo/HAO_0000931))))))) and ([has part](http://purl.obolibrary.org/obo/BFO_0000051) some ([sixth flagellomere](http://purl.obolibrary.org/obo/HAO_0001897) and ([has part](http://purl.obolibrary.org/obo/BFO_0000051) some ([ventral side](http://purl.obolibrary.org/obo/BSPO_0000068) and ([has part](http://purl.obolibrary.org/obo/BFO_0000051) some [sensillar patch](http://purl.obolibrary.org/obo/HAO_0000931)))))) and ([has part](http://purl.obolibrary.org/obo/BFO_0000051) some ([seventh flagellomere](http://purl.obolibrary.org/obo/HAO_0001898) and ([has part](http://purl.obolibrary.org/obo/BFO_0000051) some ([ventral side](http://purl.obolibrary.org/obo/BSPO_0000068) and ([has part](http://purl.obolibrary.org/obo/BFO_0000051) some [sensillar patch](http://purl.obolibrary.org/obo/HAO_0000931)))))) and ([has part](http://purl.obolibrary.org/obo/BFO_0000051) some ([eighth flagellomere](http://purl.obolibrary.org/obo/HAO_0001899) and ([has part](http://purl.obolibrary.org/obo/BFO_0000051) some ([ventral side](http://purl.obolibrary.org/obo/BSPO_0000068) and ([has part](http://purl.obolibrary.org/obo/BFO_0000051) some [sensillar patch](http://purl.obolibrary.org/obo/HAO_0000931)))))) and ([has part](http://purl.obolibrary.org/obo/BFO_0000051) some ([ninth flagellomere](http://purl.obolibrary.org/obo/HAO_0001900) and ([has part](http://purl.obolibrary.org/obo/BFO_0000051) some ([ventral side](http://purl.obolibrary.org/obo/BSPO_0000068) and ([has part](http://purl.obolibrary.org/obo/BFO_0000051) some [sensillar patch](http://purl.obolibrary.org/obo/HAO_0000931)))))) |  |
| Female flagellum ventral sensillar patch spatial arrangement: F5–F11 | (([has part](http://purl.obolibrary.org/obo/BFO_0000051) some [tenth flagellomere](http://purl.obolibrary.org/obo/HAO_0001884)) and ([has part](http://purl.obolibrary.org/obo/BFO_0000051) some ([ventral side](http://purl.obolibrary.org/obo/BSPO_0000068) and ([has part](http://purl.obolibrary.org/obo/BFO_0000051) some [sensillar patch](http://purl.obolibrary.org/obo/HAO_0000931))))) and (([has part](http://purl.obolibrary.org/obo/BFO_0000051) some [fifth flagellomere](http://purl.obolibrary.org/obo/HAO_0001885)) and ([has part](http://purl.obolibrary.org/obo/BFO_0000051) some ([ventral side](http://purl.obolibrary.org/obo/BSPO_0000068) and ([has part](http://purl.obolibrary.org/obo/BFO_0000051) some [sensillar patch](http://purl.obolibrary.org/obo/HAO_0000931))))) and (([has part](http://purl.obolibrary.org/obo/BFO_0000051) some [fourth flagellomere](http://purl.obolibrary.org/obo/HAO_0001896)) and ([has part](http://purl.obolibrary.org/obo/BFO_0000051) some ([ventral side](http://purl.obolibrary.org/obo/BSPO_0000068) and (not ([has part](http://purl.obolibrary.org/obo/BFO_0000051) some [sensillar patch](http://purl.obolibrary.org/obo/HAO_0000931)))))) and (([has part](http://purl.obolibrary.org/obo/BFO_0000051) some [sixth flagellomere](http://purl.obolibrary.org/obo/HAO_0001897)) and ([has part](http://purl.obolibrary.org/obo/BFO_0000051) some ([ventral side](http://purl.obolibrary.org/obo/BSPO_0000068) and ([has part](http://purl.obolibrary.org/obo/BFO_0000051) some [sensillar patch](http://purl.obolibrary.org/obo/HAO_0000931))))) and (([has part](http://purl.obolibrary.org/obo/BFO_0000051) some [seventh flagellomere](http://purl.obolibrary.org/obo/HAO_0001898)) and ([has part](http://purl.obolibrary.org/obo/BFO_0000051) some ([ventral side](http://purl.obolibrary.org/obo/BSPO_0000068) and ([has part](http://purl.obolibrary.org/obo/BFO_0000051) some [sensillar patch](http://purl.obolibrary.org/obo/HAO_0000931))))) and (([has part](http://purl.obolibrary.org/obo/BFO_0000051) some [eighth flagellomere](http://purl.obolibrary.org/obo/HAO_0001899)) and ([has part](http://purl.obolibrary.org/obo/BFO_0000051) some ([ventral side](http://purl.obolibrary.org/obo/BSPO_0000068) and ([has part](http://purl.obolibrary.org/obo/BFO_0000051) some [sensillar patch](http://purl.obolibrary.org/obo/HAO_0000931))))) and (([has part](http://purl.obolibrary.org/obo/BFO_0000051) some [ninth flagellomere](http://purl.obolibrary.org/obo/HAO_0001900)) and ([has part](http://purl.obolibrary.org/obo/BFO_0000051) some ([ventral side](http://purl.obolibrary.org/obo/BSPO_0000068) and ([has part](http://purl.obolibrary.org/obo/BFO_0000051) some [sensillar patch](http://purl.obolibrary.org/obo/HAO_0000931))))) and (([has part](http://purl.obolibrary.org/obo/BFO_0000051) some [eleventh flagellomere](http://purl.obolibrary.org/obo/HAO_0001901)) and ([has part](http://purl.obolibrary.org/obo/BFO_0000051) some ([ventral side](http://purl.obolibrary.org/obo/BSPO_0000068) and ([has part](http://purl.obolibrary.org/obo/BFO_0000051) some [sensillar patch](http://purl.obolibrary.org/obo/HAO_0000931))))) and ([has part](http://purl.obolibrary.org/obo/BFO_0000051) some ([apical flagellomere](http://purl.obolibrary.org/obo/HAO_0000137) and ([has part](http://purl.obolibrary.org/obo/BFO_0000051) some ([ventral side](http://purl.obolibrary.org/obo/BSPO_0000068) and (not ([has part](http://purl.obolibrary.org/obo/BFO_0000051) some [sensillar patch](http://purl.obolibrary.org/obo/HAO_0000931))))))) and ([has part](http://purl.obolibrary.org/obo/BFO_0000051) some ([first flagellomere](http://purl.obolibrary.org/obo/HAO_0001148) and ([has part](http://purl.obolibrary.org/obo/BFO_0000051) some ([ventral side](http://purl.obolibrary.org/obo/BSPO_0000068) and (not ([has part](http://purl.obolibrary.org/obo/BFO_0000051) some [sensillar patch](http://purl.obolibrary.org/obo/HAO_0000931))))))) and ([has part](http://purl.obolibrary.org/obo/BFO_0000051) some ([second flagellomere](http://purl.obolibrary.org/obo/HAO_0001883) and ([has part](http://purl.obolibrary.org/obo/BFO_0000051) some ([ventral side](http://purl.obolibrary.org/obo/BSPO_0000068) and (not ([has part](http://purl.obolibrary.org/obo/BFO_0000051) some [sensillar patch](http://purl.obolibrary.org/obo/HAO_0000931))))))) and ([has part](http://purl.obolibrary.org/obo/BFO_0000051) some ([third flagellomere](http://purl.obolibrary.org/obo/HAO_0001895) and ([has part](http://purl.obolibrary.org/obo/BFO_0000051) some ([ventral side](http://purl.obolibrary.org/obo/BSPO_0000068) and (not ([has part](http://purl.obolibrary.org/obo/BFO_0000051) some [sensillar patch](http://purl.obolibrary.org/obo/HAO_0000931))))))) | ([has part](http://purl.obolibrary.org/obo/BFO_0000051) some ([apical flagellomere](http://purl.obolibrary.org/obo/HAO_0000137) and [eleventh flagellomere](http://purl.obolibrary.org/obo/HAO_0001901) and ([has part](http://purl.obolibrary.org/obo/BFO_0000051) some ([ventral side](http://purl.obolibrary.org/obo/BSPO_0000068) and ([has part](http://purl.obolibrary.org/obo/BFO_0000051) some [sensillar patch](http://purl.obolibrary.org/obo/HAO_0000931)))))) and ([has part](http://purl.obolibrary.org/obo/BFO_0000051) some ([tenth flagellomere](http://purl.obolibrary.org/obo/HAO_0001884) and ([has part](http://purl.obolibrary.org/obo/BFO_0000051) some ([ventral side](http://purl.obolibrary.org/obo/BSPO_0000068) and ([has part](http://purl.obolibrary.org/obo/BFO_0000051) some [sensillar patch](http://purl.obolibrary.org/obo/HAO_0000931)))))) and ([has part](http://purl.obolibrary.org/obo/BFO_0000051) some ([fifth flagellomere](http://purl.obolibrary.org/obo/HAO_0001885) and ([has part](http://purl.obolibrary.org/obo/BFO_0000051) some ([ventral side](http://purl.obolibrary.org/obo/BSPO_0000068) and ([has part](http://purl.obolibrary.org/obo/BFO_0000051) some [sensillar patch](http://purl.obolibrary.org/obo/HAO_0000931)))))) and ([has part](http://purl.obolibrary.org/obo/BFO_0000051) some ([fourth flagellomere](http://purl.obolibrary.org/obo/HAO_0001896) and ([has part](http://purl.obolibrary.org/obo/BFO_0000051) some ([ventral side](http://purl.obolibrary.org/obo/BSPO_0000068) and (not ([has part](http://purl.obolibrary.org/obo/BFO_0000051) some [sensillar patch](http://purl.obolibrary.org/obo/HAO_0000931))))))) and ([has part](http://purl.obolibrary.org/obo/BFO_0000051) some ([sixth flagellomere](http://purl.obolibrary.org/obo/HAO_0001897) and ([has part](http://purl.obolibrary.org/obo/BFO_0000051) some ([ventral side](http://purl.obolibrary.org/obo/BSPO_0000068) and ([has part](http://purl.obolibrary.org/obo/BFO_0000051) some [sensillar patch](http://purl.obolibrary.org/obo/HAO_0000931)))))) and ([has part](http://purl.obolibrary.org/obo/BFO_0000051) some ([seventh flagellomere](http://purl.obolibrary.org/obo/HAO_0001898) and ([has part](http://purl.obolibrary.org/obo/BFO_0000051) some ([ventral side](http://purl.obolibrary.org/obo/BSPO_0000068) and ([has part](http://purl.obolibrary.org/obo/BFO_0000051) some [sensillar patch](http://purl.obolibrary.org/obo/HAO_0000931)))))) and ([has part](http://purl.obolibrary.org/obo/BFO_0000051) some ([eighth flagellomere](http://purl.obolibrary.org/obo/HAO_0001899) and ([has part](http://purl.obolibrary.org/obo/BFO_0000051) some ([ventral side](http://purl.obolibrary.org/obo/BSPO_0000068) and ([has part](http://purl.obolibrary.org/obo/BFO_0000051) some [sensillar patch](http://purl.obolibrary.org/obo/HAO_0000931)))))) and ([has part](http://purl.obolibrary.org/obo/BFO_0000051) some ([ninth flagellomere](http://purl.obolibrary.org/obo/HAO_0001900) and ([has part](http://purl.obolibrary.org/obo/BFO_0000051) some ([ventral side](http://purl.obolibrary.org/obo/BSPO_0000068)and ([has part](http://purl.obolibrary.org/obo/BFO_0000051) some [sensillar patch](http://purl.obolibrary.org/obo/HAO_0000931)))))) |  |
| Female metatibial spines count: present | ([has part](http://purl.obolibrary.org/obo/BFO_0000051) some [metatibia](http://purl.obolibrary.org/obo/HAO_0000631)) and ([has part](http://purl.obolibrary.org/obo/BFO_0000051) some ([dorsal side](http://purl.obolibrary.org/obo/BSPO_0000063) and ([has part](http://purl.obolibrary.org/obo/BFO_0000051) some [row of spurs](http://purl.obolibrary.org/obo/HAO_0001686)))) | [has part](http://purl.obolibrary.org/obo/BFO_0000051) some ([metatibia](http://purl.obolibrary.org/obo/HAO_0000631) and ([has part](http://purl.obolibrary.org/obo/BFO_0000051) some [spine](http://purl.obolibrary.org/obo/HAO_0000949))) |  |
| Female metatibial spines count: absent | [has part](http://purl.obolibrary.org/obo/BFO_0000051) some ([metatibia](http://purl.obolibrary.org/obo/HAO_0000631) and ([has part](http://purl.obolibrary.org/obo/BFO_0000051) some ([dorsal side](http://purl.obolibrary.org/obo/BSPO_0000063) and (not ([has part](http://purl.obolibrary.org/obo/BFO_0000051) some [row of spurs](http://purl.obolibrary.org/obo/HAO_0001686)))))) | [has part](http://purl.obolibrary.org/obo/BFO_0000051) some ([metatibia](http://purl.obolibrary.org/obo/HAO_0000631) and (not ([has part](http://purl.obolibrary.org/obo/BFO_0000051) some [spine](http://purl.obolibrary.org/obo/HAO_0000949)))) |  |
| Female OOL vs. LOL: OOL 1.0–1.2 x as long as LOL | [has part](http://purl.obolibrary.org/obo/BFO_0000051) some ([ocular ocellar line](http://purl.obolibrary.org/obo/HAO_0000662) and ([bearer of](http://purl.obolibrary.org/obo/RO_0000053) some ([length](http://purl.obolibrary.org/obo/PATO_0000122) and ([is quality measured as](http://purl.obolibrary.org/obo/IAO_0000417) some (([has measurement unit label](http://purl.obolibrary.org/obo/IAO_0000039) some ([length](http://purl.obolibrary.org/obo/PATO_0000122) and ([inheres in](http://purl.obolibrary.org/obo/RO_0000052) some [lateral ocellar line](http://purl.obolibrary.org/obo/HAO_0000480)))) and ([has measurement value](http://purl.obolibrary.org/obo/IAO_0000004) some [float](http://www.w3.org/2001/XMLSchema#float)[>= 1.0f , <= 1.1f])))))) |  |  |
| Female OOL vs. LOL: OOL 1.9–2.1 x as long as LOL | [has part](http://purl.obolibrary.org/obo/BFO_0000051) some ([ocular ocellar line](http://purl.obolibrary.org/obo/HAO_0000662) and ([bearer of](http://purl.obolibrary.org/obo/RO_0000053) some ([length](http://purl.obolibrary.org/obo/PATO_0000122) and ([is quality measured as](http://purl.obolibrary.org/obo/IAO_0000417) some (([has measurement unit label](http://purl.obolibrary.org/obo/IAO_0000039) some ([length](http://purl.obolibrary.org/obo/PATO_0000122) and ([inheres in](http://purl.obolibrary.org/obo/RO_0000052) some [lateral ocellar line](http://purl.obolibrary.org/obo/HAO_0000480)))) and ([has measurement value](http://purl.obolibrary.org/obo/IAO_0000004) some [float](http://www.w3.org/2001/XMLSchema#float)[>= 1.9f , <= 2.1f])))))) |  |  |
| Female petiole length vs. petiole width: 3.9–4.4 x as long as wide | [has part](http://purl.obolibrary.org/obo/BFO_0000051) some ([petiole length](http://purl.obolibrary.org/obo/HAO_0002262) and ([bearer of](http://purl.obolibrary.org/obo/RO_0000053) some ([length](http://purl.obolibrary.org/obo/PATO_0000122) and ([is quality measured as](http://purl.obolibrary.org/obo/IAO_0000417) some (([has measurement unit label](http://purl.obolibrary.org/obo/IAO_0000039) some ([width](http://purl.obolibrary.org/obo/PATO_0000921) and ([inheres in](http://purl.obolibrary.org/obo/RO_0000052) some ([dorsal side](http://purl.obolibrary.org/obo/BSPO_0000063) and ([part of](http://purl.obolibrary.org/obo/BFO_0000050) some [abdominal segment 2](http://purl.obolibrary.org/obo/HAO_0000020)))))) and ([has measurement value](http://purl.obolibrary.org/obo/IAO_0000004) some [float](http://www.w3.org/2001/XMLSchema#float)[>= 3.9f , <= 4.4f])))))) |  |  |
| Female petiole length vs. petiole width: 2.2–3.1 x as long as wide | [has part](http://purl.obolibrary.org/obo/BFO_0000051) some ([petiole length](http://purl.obolibrary.org/obo/HAO_0002262) and ([bearer of](http://purl.obolibrary.org/obo/RO_0000053) some ([length](http://purl.obolibrary.org/obo/PATO_0000122) and ([is quality measured as](http://purl.obolibrary.org/obo/IAO_0000417) some (([has measurement unit label](http://purl.obolibrary.org/obo/IAO_0000039) some ([width](http://purl.obolibrary.org/obo/PATO_0000921) and ([inheres in](http://purl.obolibrary.org/obo/RO_0000052) some ([dorsal side](http://purl.obolibrary.org/obo/BSPO_0000063) and ([part of](http://purl.obolibrary.org/obo/BFO_0000050) some [abdominal segment 2](http://purl.obolibrary.org/obo/HAO_0000020)))))) and ([has measurement value](http://purl.obolibrary.org/obo/IAO_0000004) some [float](http://www.w3.org/2001/XMLSchema#float)[>= 2.2f , <= 3.1f])))))) |  |  |
| Female scape length vs. compound eye height: eye height is at least 2x as long as scape length | [has part](http://purl.obolibrary.org/obo/BFO_0000051) some ([eye height](http://purl.obolibrary.org/obo/HAO_0002254) and ([bearer of](http://purl.obolibrary.org/obo/RO_0000053) some ([length](http://purl.obolibrary.org/obo/PATO_0000122) and ([is quality measured as](http://purl.obolibrary.org/obo/IAO_0000417) some (([has measurement unit label](http://purl.obolibrary.org/obo/IAO_0000039) some ([length](http://purl.obolibrary.org/obo/PATO_0000122) and ([inheres in](http://purl.obolibrary.org/obo/RO_0000052) some ([proximodistal anatomical line](http://purl.obolibrary.org/obo/HAO_0002273) and ([part of](http://purl.obolibrary.org/obo/BFO_0000050) some [scape](http://purl.obolibrary.org/obo/HAO_0000908)))))) and ([has measurement value](http://purl.obolibrary.org/obo/IAO_0000004) some [float](http://www.w3.org/2001/XMLSchema#float)[>= 2.0])))))) |  |  |
| Female scape length vs. compound eye height: greater than eye height | [has part](http://purl.obolibrary.org/obo/BFO_0000051) some ([scape](http://purl.obolibrary.org/obo/HAO_0000908) and ([has part](http://purl.obolibrary.org/obo/BFO_0000051) some ([proximodistal anatomical line](http://purl.obolibrary.org/obo/HAO_0002273) and ([bearer of](http://purl.obolibrary.org/obo/RO_0000053) some ([length](http://purl.obolibrary.org/obo/PATO_0000122) and ([increased_in_magnitude_relative_to](http://purl.obolibrary.org/obo/pato#increased_in_magnitude_relative_to) some ([length](http://purl.obolibrary.org/obo/PATO_0000122) and ([inheres in](http://purl.obolibrary.org/obo/RO_0000052) some [eye height](http://purl.obolibrary.org/obo/HAO_0002254))))))))) | [has part](http://purl.obolibrary.org/obo/BFO_0000051) some ([scape](http://purl.obolibrary.org/obo/HAO_0000908) and ([is bearer of](http://purl.obolibrary.org/obo/BFO_0000053) some ([length](http://purl.obolibrary.org/obo/PATO_0000122) and ([increased_in_magnitude_relative_to](http://purl.obolibrary.org/obo/pato#increased_in_magnitude_relative_to) some ([length](http://purl.obolibrary.org/obo/PATO_0000122) and ([inheres in](http://purl.obolibrary.org/obo/BFO_0000052) some [compound eye](http://purl.obolibrary.org/obo/HAO_0000217))))))) |  |
| Female scape length vs. compound eye height: equal to eye height | [has part](http://purl.obolibrary.org/obo/BFO_0000051) some ([scape](http://purl.obolibrary.org/obo/HAO_0000908) and (([has part](http://purl.obolibrary.org/obo/BFO_0000051) some [proximodistal anatomical line](http://purl.obolibrary.org/obo/HAO_0002273)) and ([bearer of](http://purl.obolibrary.org/obo/RO_0000053) some ([length](http://purl.obolibrary.org/obo/PATO_0000122) and ([similar_in_magnitude_relative_to](http://purl.obolibrary.org/obo/pato#similar_in_magnitude_relative_to) some ([length](http://purl.obolibrary.org/obo/PATO_0000122) and ([inheres in](http://purl.obolibrary.org/obo/RO_0000052) some [eye height](http://purl.obolibrary.org/obo/HAO_0002254)))))))) | [has part](http://purl.obolibrary.org/obo/BFO_0000051) some ([scape](http://purl.obolibrary.org/obo/HAO_0000908) and ([is bearer of](http://purl.obolibrary.org/obo/BFO_0000053) some ([length](http://purl.obolibrary.org/obo/PATO_0000122) and ([similar_in_magnitude_relative_to](http://purl.obolibrary.org/obo/pato#similar_in_magnitude_relative_to) some ([length](http://purl.obolibrary.org/obo/PATO_0000122) and ([inheres in](http://purl.obolibrary.org/obo/BFO_0000052) some [compound eye](http://purl.obolibrary.org/obo/HAO_0000217))))))) |  |
| Fore wing 1cu-a structure: tubular | [has part](http://purl.obolibrary.org/obo/BFO_0000051) some ([wing vein](http://purl.obolibrary.org/obo/HAO_0001095) and ([bearer of](http://purl.obolibrary.org/obo/RO_0000053) some [lumenized](http://purl.obolibrary.org/obo/PATO_0001897))) |  |  |
| Fore wing 1cu-a structure: not tubular, marked by darker line | [has part](http://purl.obolibrary.org/obo/BFO_0000051) some ([wing vein](http://purl.obolibrary.org/obo/HAO_0001095) and (not ([bearer of](http://purl.obolibrary.org/obo/RO_0000053) some [lumenized](http://purl.obolibrary.org/obo/PATO_0001897)))) |  |  |
| Fore wing 1CUa length vs. width: more than two times as long as wide | [has part](http://purl.obolibrary.org/obo/BFO_0000051) some ([wing vein](http://purl.obolibrary.org/obo/HAO_0001095) and ([has part](http://purl.obolibrary.org/obo/BFO_0000051) some ([proximodistal anatomical line](http://purl.obolibrary.org/obo/HAO_0002273) and ([bearer of](http://purl.obolibrary.org/obo/RO_0000053) some ([length](http://purl.obolibrary.org/obo/PATO_0000122) and ([is quality measured as](http://purl.obolibrary.org/obo/IAO_0000417) some (([has measurement unit label](http://purl.obolibrary.org/obo/IAO_0000039) some ([width](http://purl.obolibrary.org/obo/PATO_0000921) and ([inheres in](http://purl.obolibrary.org/obo/RO_0000052) some [wing vein](http://purl.obolibrary.org/obo/HAO_0001095)))) and ([has measurement value](http://purl.obolibrary.org/obo/IAO_0000004) some [float](http://www.w3.org/2001/XMLSchema#float)[> 2.0])))))))) |  |  |
| Fore wing 1CUa length vs. width: shorter than wide | ([has part](http://purl.obolibrary.org/obo/BFO_0000051) some [wing vein](http://purl.obolibrary.org/obo/HAO_0001095)) and ([bearer of](http://purl.obolibrary.org/obo/RO_0000053) some ([width](http://purl.obolibrary.org/obo/PATO_0000921) and ([increased_in_magnitude_relative_to](http://purl.obolibrary.org/obo/pato#increased_in_magnitude_relative_to) some ([length](http://purl.obolibrary.org/obo/PATO_0000122) and ([inheres in](http://purl.obolibrary.org/obo/RO_0000052) some [wing vein](http://purl.obolibrary.org/obo/HAO_0001095)))))) |  |  |
| Fore wing 1CUa orientation: oriented posterodistally | [has part](http://purl.obolibrary.org/obo/BFO_0000051) some ([wing vein](http://purl.obolibrary.org/obo/HAO_0001095) and ([bearer of](http://purl.obolibrary.org/obo/RO_0000053) some [posterodistal_orientation](http://hymao.org/miko2014/phenotypes.owl#posterodistal_orientation))) |  |  |
| Fore wing 1CUa orientation: oriented anterodistally | [has part](http://purl.obolibrary.org/obo/BFO_0000051) some ([wing vein](http://purl.obolibrary.org/obo/HAO_0001095) and ([bearer of](http://purl.obolibrary.org/obo/RO_0000053) some [anterodistal_orientation](http://hymao.org/miko2014/phenotypes.owl#anterodistal_orientation))) |  |  |
| Fore wing 1RS count: present | [has part](http://purl.obolibrary.org/obo/BFO_0000051) some [wing vein](http://purl.obolibrary.org/obo/HAO_0001095) |  |  |
| Fore wing 1RS count: absent | not ([phenotype_23454](http://hymao.org/miko2014/phenotypes.owl#phenotype_23454)) |  |  |
| Fore wing 2A count: absent | not ([phenotype_23452](http://hymao.org/miko2014/phenotypes.owl#phenotype_23452)) |  |  |
| Fore wing 2A count: present | [has part](http://purl.obolibrary.org/obo/BFO_0000051) some [wing vein](http://purl.obolibrary.org/obo/HAO_0001095) |  |  |
| Fore wing 2R1 count: present | [has part](http://purl.obolibrary.org/obo/BFO_0000051) some [wing vein](http://purl.obolibrary.org/obo/HAO_0001095) |  |  |
| Fore wing 2R1 count: absent | not ([phenotype_23450](http://hymao.org/miko2014/phenotypes.owl#phenotype_23450)) |  |  |
| Fore wing 2RS count: absent | not ([phenotype_23458](http://hymao.org/miko2014/phenotypes.owl#phenotype_23458)) |  |  |
| Fore wing 2RS count: present | [has part](http://purl.obolibrary.org/obo/BFO_0000051) some [wing vein](http://purl.obolibrary.org/obo/HAO_0001095) |  |  |
| Fore wing 3CU distal region count: present | [has part](http://purl.obolibrary.org/obo/BFO_0000051) some [wing vein](http://purl.obolibrary.org/obo/HAO_0001095) |  |  |
| Fore wing 3CU distal region count: absent | not ([phenotype_23462](http://hymao.org/miko2014/phenotypes.owl#phenotype_23462)) |  |  |
| Fore wing 3M count: present | [has part](http://purl.obolibrary.org/obo/BFO_0000051) some [wing vein](http://purl.obolibrary.org/obo/HAO_0001095) |  |  |
| Fore wing 3M count: absent | not ([phenotype_23464](http://hymao.org/miko2014/phenotypes.owl#phenotype_23464)) |  |  |
| Fore wing distal margin in flexed position vs. metasoma distal margin: extending beyond posterior margin of metasoma | [has part](http://purl.obolibrary.org/obo/BFO_0000051) some ([fore wing](http://purl.obolibrary.org/obo/HAO_0000351) and ([has part](http://purl.obolibrary.org/obo/BFO_0000051) some ([distal margin](http://purl.obolibrary.org/obo/BSPO_0000678) and ([posterior_to](http://purl.obolibrary.org/obo/BSPO_0000099) some ([posterior margin](http://purl.obolibrary.org/obo/BSPO_0000672) and ([part of](http://purl.obolibrary.org/obo/BFO_0000050) some [metasoma](http://purl.obolibrary.org/obo/HAO_0000626))))))) |  | [has_part](http://purl.obolibrary.org/obo/BFO_0000051) some ([fore wing](http://purl.obolibrary.org/obo/HAO_0000351) and ([is bearer of](http://purl.obolibrary.org/obo/BFO_0000053) some ([length](http://purl.obolibrary.org/obo/PATO_0000122) and ([increased_in_magnitude_relative_to](http://purl.obolibrary.org/obo/pato#increased_in_magnitude_relative_to) some ([length](http://purl.obolibrary.org/obo/PATO_0000122) and ([inheres in](http://purl.obolibrary.org/obo/BFO_0000052) some metasoma)))))) |
| Fore wing m-cu structure: tubular | [has part](http://purl.obolibrary.org/obo/BFO_0000051) some ([wing vein](http://purl.obolibrary.org/obo/HAO_0001095) and ([bearer of](http://purl.obolibrary.org/obo/RO_0000053) some [lumenized](http://purl.obolibrary.org/obo/PATO_0001897))) |  |  |
| Fore wing m-cu structure: not tubular, marked by dark line | [has part](http://purl.obolibrary.org/obo/BFO_0000051) some ([wing vein](http://purl.obolibrary.org/obo/HAO_0001095) and (not ([bearer of](http://purl.obolibrary.org/obo/RO_0000053) some [lumenized](http://purl.obolibrary.org/obo/PATO_0001897)))) |  |  |
| Fore wing RS+M structure: tubular | [has part](http://purl.obolibrary.org/obo/BFO_0000051) some ([wing vein](http://purl.obolibrary.org/obo/HAO_0001095) and ([bearer of](http://purl.obolibrary.org/obo/RO_0000053) some [lumenized](http://purl.obolibrary.org/obo/PATO_0001897))) |  |  |
| Fore wing RS+M structure: not tubular, marked by dark line | [has part](http://purl.obolibrary.org/obo/BFO_0000051) some ([wing vein](http://purl.obolibrary.org/obo/HAO_0001095) and (not ([bearer of](http://purl.obolibrary.org/obo/RO_0000053) some [lumenized](http://purl.obolibrary.org/obo/PATO_0001897)))) |  |  |
| Fore wing vein 3RS structure: tubular | [has part](http://purl.obolibrary.org/obo/BFO_0000051) some ([wing vein](http://purl.obolibrary.org/obo/HAO_0001095) and ([bearer of](http://purl.obolibrary.org/obo/RO_0000053) some [lumenized](http://purl.obolibrary.org/obo/PATO_0001897))) |  |  |
| Fore wing vein 3RS structure: not tubular, marked by dark line | [has part](http://purl.obolibrary.org/obo/BFO_0000051) some ([wing vein](http://purl.obolibrary.org/obo/HAO_0001095) and (not ([bearer of](http://purl.obolibrary.org/obo/RO_0000053) some [lumenized](http://purl.obolibrary.org/obo/PATO_0001897)))) |  |  |
| Fore wing vein r-rs structure: tubular | [has part](http://purl.obolibrary.org/obo/BFO_0000051) some ([wing vein](http://purl.obolibrary.org/obo/HAO_0001095) and ([bearer of](http://purl.obolibrary.org/obo/RO_0000053) some [lumenized](http://purl.obolibrary.org/obo/PATO_0001897))) |  |  |
| Fore wing vein r-rs structure: not tubular, marked by dark line | [has part](http://purl.obolibrary.org/obo/BFO_0000051) some ([wing vein](http://purl.obolibrary.org/obo/HAO_0001095) and (not ([bearer of](http://purl.obolibrary.org/obo/RO_0000053) some [lumenized](http://purl.obolibrary.org/obo/PATO_0001897)))) |  |  |
| Gaster color: light brown | [has part](http://purl.obolibrary.org/obo/BFO_0000051) some ([gaster](http://purl.obolibrary.org/obo/HAO_0000369) and ([bearer of](http://purl.obolibrary.org/obo/RO_0000053) some [light brown](http://purl.obolibrary.org/obo/PATO_0001246))) |  |  |
| Gaster color: dark brown | [has part](http://purl.obolibrary.org/obo/BFO_0000051) some ([gaster](http://purl.obolibrary.org/obo/HAO_0000369) and ([bearer of](http://purl.obolibrary.org/obo/RO_0000053) some [dark brown](http://purl.obolibrary.org/obo/PATO_0001245))) |  |  |
| Gaster color: brown | [has part](http://purl.obolibrary.org/obo/BFO_0000051) some ([gaster](http://purl.obolibrary.org/obo/HAO_0000369) and ([bearer of](http://purl.obolibrary.org/obo/RO_0000053) some [brown](http://purl.obolibrary.org/obo/PATO_0000952))) |  |  |
| Gastral scrobe count: absent | not ([has part](http://purl.obolibrary.org/obo/BFO_0000051) some [gastral scrobe](http://purl.obolibrary.org/obo/HAO_0001933)) |  |  |
| Gastral scrobe count: present | [has part](http://purl.obolibrary.org/obo/BFO_0000051) some [gastral scrobe](http://purl.obolibrary.org/obo/HAO_0001933) |  |  |
| Head width vs. IOS: head 1.8-2.0 times as wide as IOS | [has part](http://purl.obolibrary.org/obo/BFO_0000051) some ([head width](http://purl.obolibrary.org/obo/HAO_0002268) and ([bearer of](http://purl.obolibrary.org/obo/RO_0000053) some ([length](http://purl.obolibrary.org/obo/PATO_0000122) and ([is quality measured as](http://purl.obolibrary.org/obo/IAO_0000417) some (([has measurement unit label](http://purl.obolibrary.org/obo/IAO_0000039) some ([length](http://purl.obolibrary.org/obo/PATO_0000122) and ([inheres in](http://purl.obolibrary.org/obo/RO_0000052) some [interorbital space](http://purl.obolibrary.org/obo/HAO_0000432)))) and ([has measurement value](http://purl.obolibrary.org/obo/IAO_0000004) some [float](http://www.w3.org/2001/XMLSchema#float)[>= 1.8f , <= 2.0f])))))) |  |  |
| Head width vs. IOS: head about 1.5 times as wide as IOS | [has part](http://purl.obolibrary.org/obo/BFO_0000051) some ([head width](http://purl.obolibrary.org/obo/HAO_0002268) and ([bearer of](http://purl.obolibrary.org/obo/RO_0000053) some ([length](http://purl.obolibrary.org/obo/PATO_0000122) and ([is quality measured as](http://purl.obolibrary.org/obo/IAO_0000417) some (([has measurement unit label](http://purl.obolibrary.org/obo/IAO_0000039) some ([length](http://purl.obolibrary.org/obo/PATO_0000122) and ([inheres in](http://purl.obolibrary.org/obo/RO_0000052) some [interorbital space](http://purl.obolibrary.org/obo/HAO_0000432)))) and ([has measurement value](http://purl.obolibrary.org/obo/IAO_0000004) value 1.5)))))) |  |  |
| Head+mesosoma median length vs. mesosoma height: Head+mesosoma median length 0.9–1.1 x as long as mesosoma height | [has part](http://purl.obolibrary.org/obo/BFO_0000051) some ([head+mesosoma length](http://purl.obolibrary.org/obo/HAO_0002274) and ([bearer of](http://purl.obolibrary.org/obo/RO_0000053) some ([length](http://purl.obolibrary.org/obo/PATO_0000122) and ([is quality measured as](http://purl.obolibrary.org/obo/IAO_0000417) some (([has measurement unit label](http://purl.obolibrary.org/obo/IAO_0000039) some ([length](http://purl.obolibrary.org/obo/PATO_0000122) and ([inheres in](http://purl.obolibrary.org/obo/RO_0000052) some [mesosoma height](http://purl.obolibrary.org/obo/HAO_0002251)))) and ([has measurement value](http://purl.obolibrary.org/obo/IAO_0000004) some [float](http://www.w3.org/2001/XMLSchema#float)[>= 0.9f , <= 1.1f])))))) |  |  |
| Head+mesosoma median length vs. mesosoma height: Head+mesosoma median length 1.8 x as long as mesosoma height | [has part](http://purl.obolibrary.org/obo/BFO_0000051) some ([head+mesosoma length](http://purl.obolibrary.org/obo/HAO_0002274) and ([bearer of](http://purl.obolibrary.org/obo/RO_0000053) some ([length](http://purl.obolibrary.org/obo/PATO_0000122) and ([is quality measured as](http://purl.obolibrary.org/obo/IAO_0000417) some (([has measurement unit label](http://purl.obolibrary.org/obo/IAO_0000039) some ([length](http://purl.obolibrary.org/obo/PATO_0000122) and ([inheres in](http://purl.obolibrary.org/obo/RO_0000052) some [mesosoma height](http://purl.obolibrary.org/obo/HAO_0002251)))) and ([has measurement value](http://purl.obolibrary.org/obo/IAO_0000004) value 1.8)))))) |  |  |
| Lateroventral carina of the petiole count: present | [has part](http://purl.obolibrary.org/obo/BFO_0000051) some [lateroventral carina of the petiole](http://purl.obolibrary.org/obo/HAO_0002266) | [has part](http://purl.obolibrary.org/obo/BFO_0000051) some ([abdominal segment 2](http://purl.obolibrary.org/obo/HAO_0000020) and ([has part](http://purl.obolibrary.org/obo/BFO_0000051) some ([ventro-lateral region](http://purl.obolibrary.org/obo/BSPO_0000085) and ([has part](http://purl.obolibrary.org/obo/BFO_0000051) some [carina](http://purl.obolibrary.org/obo/HAO_0000188))))) |  |
| Lateroventral carina of the petiole count: absent | not ([has part](http://purl.obolibrary.org/obo/BFO_0000051) some [lateroventral carina of the petiole](http://purl.obolibrary.org/obo/HAO_0002266)) | [has part](http://purl.obolibrary.org/obo/BFO_0000051) some ([abdominal segment 2](http://purl.obolibrary.org/obo/HAO_0000020) and ([has part](http://purl.obolibrary.org/obo/BFO_0000051) some ([ventro-lateral region](http://purl.obolibrary.org/obo/BSPO_0000085) and (not ([has part](http://purl.obolibrary.org/obo/BFO_0000051) some [carina](http://purl.obolibrary.org/obo/HAO_0000188)))))) |  |
| Malar distance vs. eye height: eye 1.5 times as high as malar distance | [has part](http://purl.obolibrary.org/obo/BFO_0000051) some ([eye height](http://purl.obolibrary.org/obo/HAO_0002254) and ([bearer of](http://purl.obolibrary.org/obo/RO_0000053) some ([length](http://purl.obolibrary.org/obo/PATO_0000122) and ([is quality measured as](http://purl.obolibrary.org/obo/IAO_0000417) some (([has measurement unit label](http://purl.obolibrary.org/obo/IAO_0000039) some ([length](http://purl.obolibrary.org/obo/PATO_0000122) and ([inheres in](http://purl.obolibrary.org/obo/RO_0000052) some [malar line](http://purl.obolibrary.org/obo/HAO_0000503)))) and ([has measurement value](http://purl.obolibrary.org/obo/IAO_0000004) value 1.5)))))) |  |  |
| Malar distance vs. eye height: eye 2x as high as malar distance | [has part](http://purl.obolibrary.org/obo/BFO_0000051) some ([eye height](http://purl.obolibrary.org/obo/HAO_0002254) and ([bearer of](http://purl.obolibrary.org/obo/RO_0000053) some ([length](http://purl.obolibrary.org/obo/PATO_0000122) and ([is quality measured as](http://purl.obolibrary.org/obo/IAO_0000417) some (([has measurement unit label](http://purl.obolibrary.org/obo/IAO_0000039) some ([length](http://purl.obolibrary.org/obo/PATO_0000122) and ([inheres in](http://purl.obolibrary.org/obo/RO_0000052) some [malar line](http://purl.obolibrary.org/obo/HAO_0000503)))) and ([has measurement value](http://purl.obolibrary.org/obo/IAO_0000004) value 2.0)))))) |  |  |
| Male petiole length vs. width: 4.9–5.2x as long as wide | [has part](http://purl.obolibrary.org/obo/BFO_0000051) some ([petiole length](http://purl.obolibrary.org/obo/HAO_0002262) and ([bearer of](http://purl.obolibrary.org/obo/RO_0000053) some ([length](http://purl.obolibrary.org/obo/PATO_0000122) and ([is quality measured as](http://purl.obolibrary.org/obo/IAO_0000417) some (([has measurement unit label](http://purl.obolibrary.org/obo/IAO_0000039) some ([width](http://purl.obolibrary.org/obo/PATO_0000921) and ([inheres in](http://purl.obolibrary.org/obo/RO_0000052) some [abdominal segment 2](http://purl.obolibrary.org/obo/HAO_0000020)))) and ([has measurement value](http://purl.obolibrary.org/obo/IAO_0000004) some [float](http://www.w3.org/2001/XMLSchema#float)[>= 4.9f , <= 5.2f])))))) |  |  |
| Male petiole length vs. width: 2.5 x as long as wide | [has part](http://purl.obolibrary.org/obo/BFO_0000051) some ([petiole length](http://purl.obolibrary.org/obo/HAO_0002262) and ([bearer of](http://purl.obolibrary.org/obo/RO_0000053) some ([length](http://purl.obolibrary.org/obo/PATO_0000122) and ([is quality measured as](http://purl.obolibrary.org/obo/IAO_0000417) some (([has measurement unit label](http://purl.obolibrary.org/obo/IAO_0000039) some ([width](http://purl.obolibrary.org/obo/PATO_0000921) and ([inheres in](http://purl.obolibrary.org/obo/RO_0000052) some [abdominal segment 2](http://purl.obolibrary.org/obo/HAO_0000020)))) and ([has measurement value](http://purl.obolibrary.org/obo/IAO_0000004) value 2.5)))))) |  |  |
| Male petiole length vs. width: 3.0 x as long as wide | [has part](http://purl.obolibrary.org/obo/BFO_0000051) some ([petiole length](http://purl.obolibrary.org/obo/HAO_0002262) and ([bearer of](http://purl.obolibrary.org/obo/RO_0000053) some ([length](http://purl.obolibrary.org/obo/PATO_0000122) and ([is quality measured as](http://purl.obolibrary.org/obo/IAO_0000417) some (([has measurement unit label](http://purl.obolibrary.org/obo/IAO_0000039) some ([width](http://purl.obolibrary.org/obo/PATO_0000921) and ([inheres in](http://purl.obolibrary.org/obo/RO_0000052) some [abdominal segment 2](http://purl.obolibrary.org/obo/HAO_0000020)))) and ([has measurement value](http://purl.obolibrary.org/obo/IAO_0000004) value 3.0)))))) |  |  |
| Male petiole length vs. width: 3.5–4.0 x as long as wide | [has part](http://purl.obolibrary.org/obo/BFO_0000051) some ([petiole length](http://purl.obolibrary.org/obo/HAO_0002262) and ([bearer of](http://purl.obolibrary.org/obo/RO_0000053) some ([length](http://purl.obolibrary.org/obo/PATO_0000122) and ([is quality measured as](http://purl.obolibrary.org/obo/IAO_0000417) some (([has measurement unit label](http://purl.obolibrary.org/obo/IAO_0000039) some ([width](http://purl.obolibrary.org/obo/PATO_0000921) and ([inheres in](http://purl.obolibrary.org/obo/RO_0000052) some [abdominal segment 2](http://purl.obolibrary.org/obo/HAO_0000020)))) and ([has measurement value](http://purl.obolibrary.org/obo/IAO_0000004) some [float](http://www.w3.org/2001/XMLSchema#float)[>= 3.5f , <= 4.0f])))))) |  |  |
| Mandible color: black with dark brown mandibular teeth | ([has part](http://purl.obolibrary.org/obo/BFO_0000051) some ([tooth](http://purl.obolibrary.org/obo/HAO_0001019) and ([bearer of](http://purl.obolibrary.org/obo/RO_0000053) some [dark brown](http://purl.obolibrary.org/obo/PATO_0001245)))) and ([has part](http://purl.obolibrary.org/obo/BFO_0000051) some ((not ([tooth](http://purl.obolibrary.org/obo/HAO_0001019))) and ([part of](http://purl.obolibrary.org/obo/BFO_0000050) some ([mandible](http://purl.obolibrary.org/obo/HAO_0000506) and ([bearer of](http://purl.obolibrary.org/obo/RO_0000053) some [black](http://purl.obolibrary.org/obo/PATO_0000317)))))) |  |  |
| Mandible color: brown | [has part](http://purl.obolibrary.org/obo/BFO_0000051) some ([mandible](http://purl.obolibrary.org/obo/HAO_0000506) and ([bearer of](http://purl.obolibrary.org/obo/RO_0000053) some [brown](http://purl.obolibrary.org/obo/PATO_0000952))) |  |  |
| Mandible color: yellowish | [has part](http://purl.obolibrary.org/obo/BFO_0000051) some ([mandible](http://purl.obolibrary.org/obo/HAO_0000506) and ([bearer of](http://purl.obolibrary.org/obo/RO_0000053) some [yellow](http://purl.obolibrary.org/obo/PATO_0000324))) |  |  |
| Mandibular teeth count: 3 | [has part](http://purl.obolibrary.org/obo/BFO_0000051) some ([mandible](http://purl.obolibrary.org/obo/HAO_0000506) and ([has component](http://purl.obolibrary.org/obo/RO_0002180) exactly 3 [tooth](http://purl.obolibrary.org/obo/HAO_0001019))) | [has part](http://purl.obolibrary.org/obo/BFO_0000051) some ([mandible](http://purl.obolibrary.org/obo/HAO_0000506) and ([has component](http://purl.obolibrary.org/obo/RO_0002180) exactly 3 [tooth](http://purl.obolibrary.org/obo/HAO_0001019))) |  |
| Mandibular teeth count: 4 | [has part](http://purl.obolibrary.org/obo/BFO_0000051) some ([mandible](http://purl.obolibrary.org/obo/HAO_0000506) and ([has component](http://purl.obolibrary.org/obo/RO_0002180) exactly 4 [tooth](http://purl.obolibrary.org/obo/HAO_0001019))) | [has part](http://purl.obolibrary.org/obo/BFO_0000051) some ([mandible](http://purl.obolibrary.org/obo/HAO_0000506) and ([has component](http://purl.obolibrary.org/obo/RO_0002180) exactly 4 [tooth](http://purl.obolibrary.org/obo/HAO_0001019))) |  |
| Median clypeal projection count: absent | [has part](http://purl.obolibrary.org/obo/BFO_0000051) some ([clypeus](http://purl.obolibrary.org/obo/HAO_0000212) and ([has part](http://purl.obolibrary.org/obo/BFO_0000051) some ([ventro-medial margin](http://purl.obolibrary.org/obo/BSPO_0000049) and (not ([has part](http://purl.obolibrary.org/obo/BFO_0000051) some [projection](http://purl.obolibrary.org/obo/HAO_0000829)))))) |  |  |
| Median clypeal projection count: present | [has part](http://purl.obolibrary.org/obo/BFO_0000051) some ([clypeus](http://purl.obolibrary.org/obo/HAO_0000212) and ([has part](http://purl.obolibrary.org/obo/BFO_0000051) some ([ventro-medial margin](http://purl.obolibrary.org/obo/BSPO_0000049) and ([has part](http://purl.obolibrary.org/obo/BFO_0000051) some [projection](http://purl.obolibrary.org/obo/HAO_0000829))))) |  |  |
| Median clypeal projection sharpness: blunt | [has part](http://purl.obolibrary.org/obo/BFO_0000051) some ([clypeus](http://purl.obolibrary.org/obo/HAO_0000212) and ([has part](http://purl.obolibrary.org/obo/BFO_0000051) some ([ventro-medial margin](http://purl.obolibrary.org/obo/BSPO_0000049) and ([has part](http://purl.obolibrary.org/obo/BFO_0000051) some ([projection](http://purl.obolibrary.org/obo/HAO_0000829) and ([bearer of](http://purl.obolibrary.org/obo/RO_0000053) some [blunt](http://purl.obolibrary.org/obo/PATO_0001950))))))) | [has part](http://purl.obolibrary.org/obo/BFO_0000051) some ([clypeus](http://purl.obolibrary.org/obo/HAO_0000212) and ([has part](http://purl.obolibrary.org/obo/BFO_0000051) some ([ventro-medial margin](http://purl.obolibrary.org/obo/BSPO_0000049) and ([has part](http://purl.obolibrary.org/obo/BFO_0000051) some ([projection](http://purl.obolibrary.org/obo/HAO_0000829) and ([is bearer of](http://purl.obolibrary.org/obo/BFO_0000053) some [blunt](http://purl.obolibrary.org/obo/PATO_0001950))))))) |  |
| Median clypeal projection sharpness: pointed | [has part](http://purl.obolibrary.org/obo/BFO_0000051) some ([clypeus](http://purl.obolibrary.org/obo/HAO_0000212) and ([has part](http://purl.obolibrary.org/obo/BFO_0000051) some ([ventro-medial margin](http://purl.obolibrary.org/obo/BSPO_0000049) and ([has part](http://purl.obolibrary.org/obo/BFO_0000051) some ([projection](http://purl.obolibrary.org/obo/HAO_0000829) and ([bearer of](http://purl.obolibrary.org/obo/RO_0000053) some [pointed](http://purl.obolibrary.org/obo/PATO_0002258))))))) | [has part](http://purl.obolibrary.org/obo/BFO_0000051) some ([clypeus](http://purl.obolibrary.org/obo/HAO_0000212) and ([has part](http://purl.obolibrary.org/obo/BFO_0000051) some ([ventro-medial margin](http://purl.obolibrary.org/obo/BSPO_0000049) and ([has part](http://purl.obolibrary.org/obo/BFO_0000051) some ([projection](http://purl.obolibrary.org/obo/HAO_0000829) and ([is bearer of](http://purl.obolibrary.org/obo/BFO_0000053) some [pointed](http://purl.obolibrary.org/obo/PATO_0002258))))))) |  |
| Median conjunctiva of male abdominal tergum 9 count: present | ([has part](http://purl.obolibrary.org/obo/BFO_0000051) some [male genitalia](http://purl.obolibrary.org/obo/HAO_0000505)) and ([has part](http://purl.obolibrary.org/obo/BFO_0000051) some [median conjunctiva of abdominal tergum 9](http://purl.obolibrary.org/obo/HAO_0002267)) |  |  |
| Median conjunctiva of male abdominal tergum 9 count: absent | (not ([has part](http://purl.obolibrary.org/obo/BFO_0000051) some [median conjunctiva of abdominal tergum 9](http://purl.obolibrary.org/obo/HAO_0002267))) and ([has part](http://purl.obolibrary.org/obo/BFO_0000051) some [male genitalia](http://purl.obolibrary.org/obo/HAO_0000505)) |  |  |
| Mesopleural carina count: absent | not ([has part](http://purl.obolibrary.org/obo/BFO_0000051) some [mesopleural carina](http://purl.obolibrary.org/obo/HAO_0000559)) |  |  |
| Mesopleural carina count: present | [has part](http://purl.obolibrary.org/obo/BFO_0000051) some [mesopleural carina](http://purl.obolibrary.org/obo/HAO_0000559) |  |  |
| Mesoscutal humeral sulcus anterior end vs. preaxilla anterior end: mesoscutal humeral sulcus anterior end is posterior to dorsal margin of preaxilla anterior end | [has part](http://purl.obolibrary.org/obo/BFO_0000051) some ([mesoscutal humeral sulcus](http://purl.obolibrary.org/obo/HAO_0000569) and ([has part](http://purl.obolibrary.org/obo/BFO_0000051) some ([anterior margin](http://purl.obolibrary.org/obo/BSPO_0000671) and ([posterior_to](http://purl.obolibrary.org/obo/BSPO_0000099) some ([anterior margin](http://purl.obolibrary.org/obo/BSPO_0000671) and ([part of](http://purl.obolibrary.org/obo/BFO_0000050) some [preaxilla](http://purl.obolibrary.org/obo/HAO_0000800))))))) | [has part](http://purl.obolibrary.org/obo/BFO_0000051) some ([mesoscutal suprahumeral sulcus](http://purl.obolibrary.org/obo/HAO_0000570) and ([is bearer of](http://purl.obolibrary.org/obo/BFO_0000053) some [split](http://purl.obolibrary.org/obo/PATO_0001786))) |  |
| Mesoscutal humeral sulcus anterior end vs. preaxilla anterior end: mesoscutal humeral sulcus anterior end is adjacent to dorsal margin of preaxilla anterior end | [has part](http://purl.obolibrary.org/obo/BFO_0000051) some ([mesoscutal humeral sulcus](http://purl.obolibrary.org/obo/HAO_0000569) and ([has part](http://purl.obolibrary.org/obo/BFO_0000051) some ([anterior margin](http://purl.obolibrary.org/obo/BSPO_0000671) and ([adjacent_to](http://www.obofoundry.org/ro/ro.owl#adjacent_to) some ([anterior margin](http://purl.obolibrary.org/obo/BSPO_0000671) and ([part of](http://purl.obolibrary.org/obo/BFO_0000050) some [preaxilla](http://purl.obolibrary.org/obo/HAO_0000800))))))) | [has part](http://purl.obolibrary.org/obo/BFO_0000051) some ([mesoscutal suprahumeral sulcus](http://purl.obolibrary.org/obo/HAO_0000570) and ([is bearer of](http://purl.obolibrary.org/obo/BFO_0000053) some [undivided](http://purl.obolibrary.org/obo/PATO_0002034))) |  |
| Mesoscutellum median length vs. anteromesoscutum median length: mesoscutellum longer than or equals anteromesoscutum | [has part](http://purl.obolibrary.org/obo/BFO_0000051) some ([mesoscutellum](http://purl.obolibrary.org/obo/HAO_0000574) and ([has part](http://purl.obolibrary.org/obo/BFO_0000051) some ([dorsal side](http://purl.obolibrary.org/obo/BSPO_0000063) and (([has part](http://purl.obolibrary.org/obo/BFO_0000051) some [median anatomical line](http://purl.obolibrary.org/obo/HAO_0002272)) and ([bearer of](http://purl.obolibrary.org/obo/RO_0000053) some ([length](http://purl.obolibrary.org/obo/PATO_0000122) and ([is quality measured as](http://purl.obolibrary.org/obo/IAO_0000417) some (([has measurement unit label](http://purl.obolibrary.org/obo/IAO_0000039) some ([length](http://purl.obolibrary.org/obo/PATO_0000122) and ([inheres in](http://purl.obolibrary.org/obo/RO_0000052) some ([median anatomical line](http://purl.obolibrary.org/obo/HAO_0002272) and (([part of](http://purl.obolibrary.org/obo/BFO_0000050) some [dorsal side](http://purl.obolibrary.org/obo/BSPO_0000063)) and ([part of](http://purl.obolibrary.org/obo/BFO_0000050) some [anteromesoscutum](http://purl.obolibrary.org/obo/HAO_0001490))))))) and ([has measurement value](http://purl.obolibrary.org/obo/IAO_0000004) some [float](http://www.w3.org/2001/XMLSchema#float)[>= 1.0f]))))))))) |  |  |
| Mesoscutellum median length vs. anteromesoscutum median length: mesoscutellum shorter than anteromesoscutum | [has part](http://purl.obolibrary.org/obo/BFO_0000051) some ([mesoscutellum](http://purl.obolibrary.org/obo/HAO_0000574) and ([has part](http://purl.obolibrary.org/obo/BFO_0000051) some ([dorsal side](http://purl.obolibrary.org/obo/BSPO_0000063) and (([has part](http://purl.obolibrary.org/obo/BFO_0000051) some [median anatomical line](http://purl.obolibrary.org/obo/HAO_0002272)) and ([bearer of](http://purl.obolibrary.org/obo/RO_0000053) some ([length](http://purl.obolibrary.org/obo/PATO_0000122) and ([decreased_in_magnitude_relative_to](http://purl.obolibrary.org/obo/pato#decreased_in_magnitude_relative_to) some ([length](http://purl.obolibrary.org/obo/PATO_0000122) and ([inheres in](http://purl.obolibrary.org/obo/RO_0000052) some ([median anatomical line](http://purl.obolibrary.org/obo/HAO_0002272) and ([part of](http://purl.obolibrary.org/obo/BFO_0000050) some ([dorsal side](http://purl.obolibrary.org/obo/BSPO_0000063) and ([part of](http://purl.obolibrary.org/obo/BFO_0000050) some [anteromesoscutum](http://purl.obolibrary.org/obo/HAO_0001490)))))))))))))) |  |  |
| Mesoscutellum posterior margin vs. metapectal-propodeal complex anterior margin medially: Posterior margin of mesoscutellum adjacent medially to anterior margin of metapectal-propodeal complex | [has part](http://purl.obolibrary.org/obo/BFO_0000051) some ([mesoscutellum](http://purl.obolibrary.org/obo/HAO_0000574) and ([has part](http://purl.obolibrary.org/obo/BFO_0000051) some ([posterior margin](http://purl.obolibrary.org/obo/BSPO_0000672) and ([adjacent_to](http://www.obofoundry.org/ro/ro.owl#adjacent_to) some ([anterior margin](http://purl.obolibrary.org/obo/BSPO_0000671) and ([part of](http://purl.obolibrary.org/obo/BFO_0000050) some [metapectal-propodeal complex](http://purl.obolibrary.org/obo/HAO_0000604))))))) |  |  |
| Mesoscutellum posterior margin vs. metapectal-propodeal complex anterior margin medially: Posterior margin of mesoscutellum not adjacent medially to anterior margin of metapectal-propodeal complex | [has part](http://purl.obolibrary.org/obo/BFO_0000051) some ([mesoscutellum](http://purl.obolibrary.org/obo/HAO_0000574) and ([has part](http://purl.obolibrary.org/obo/BFO_0000051) some ([posterior margin](http://purl.obolibrary.org/obo/BSPO_0000672) and (not ([adjacent_to](http://www.obofoundry.org/ro/ro.owl#adjacent_to) some ([anterior margin](http://purl.obolibrary.org/obo/BSPO_0000671) and ([part of](http://purl.obolibrary.org/obo/BFO_0000050) some [metapectal-propodeal complex](http://purl.obolibrary.org/obo/HAO_0000604)))))))) |  |  |
| Mesosoma color: black tegula light brown | ([has part](http://purl.obolibrary.org/obo/BFO_0000051) some ([mesosoma](http://purl.obolibrary.org/obo/HAO_0000576) and ([has part](http://purl.obolibrary.org/obo/BFO_0000051) some ((not ([tegula](http://purl.obolibrary.org/obo/HAO_0000993))) and ([bearer of](http://purl.obolibrary.org/obo/RO_0000053) some [black](http://purl.obolibrary.org/obo/PATO_0000317)))))) and ([has part](http://purl.obolibrary.org/obo/BFO_0000051) some ([tegula](http://purl.obolibrary.org/obo/HAO_0000993) and ([bearer of](http://purl.obolibrary.org/obo/RO_0000053) some [light brown](http://purl.obolibrary.org/obo/PATO_0001246)))) |  |  |
| Mesosoma color: black tegula dark brown | ([has part](http://purl.obolibrary.org/obo/BFO_0000051) some ([mesosoma](http://purl.obolibrary.org/obo/HAO_0000576) and ([has part](http://purl.obolibrary.org/obo/BFO_0000051) some ((not ([tegula](http://purl.obolibrary.org/obo/HAO_0000993))) and ([bearer of](http://purl.obolibrary.org/obo/RO_0000053) some [black](http://purl.obolibrary.org/obo/PATO_0000317)))))) and ([has part](http://purl.obolibrary.org/obo/BFO_0000051) some ([tegula](http://purl.obolibrary.org/obo/HAO_0000993) and ([bearer of](http://purl.obolibrary.org/obo/RO_0000053) some [dark brown](http://purl.obolibrary.org/obo/PATO_0001245)))) |  |  |
| Metanoto-metapectal-propodeal complex conjunctiva count: present (metanotum and metapectal propodeal complex separated) | [has part](http://purl.obolibrary.org/obo/BFO_0000051) some [metanoto-metapectal-propodeal conjunctiva](http://purl.obolibrary.org/obo/HAO_0002257) |  |  |
| Metanoto-metapectal-propodeal complex conjunctiva count: absent (metanotum and metapectal-propodeal complex fused) | not ([has part](http://purl.obolibrary.org/obo/BFO_0000051) some [metanoto-metapectal-propodeal conjunctiva](http://purl.obolibrary.org/obo/HAO_0002257)) |  |  |
| Metapleural sulcus position: vertical | [has part](http://purl.obolibrary.org/obo/BFO_0000051) some ([metapleural sulcus](http://purl.obolibrary.org/obo/HAO_0000614) and ([bearer of](http://purl.obolibrary.org/obo/RO_0000053) some [vertical](http://purl.obolibrary.org/obo/PATO_0001854))) | [has part](http://purl.obolibrary.org/obo/BFO_0000051) some ([metapleural sulcus](http://purl.obolibrary.org/obo/HAO_0000614) and ([is bearer of](http://purl.obolibrary.org/obo/BFO_0000053) some [vertical](http://purl.obolibrary.org/obo/PATO_0001854))) |  |
| Metapleural sulcus position: horizontal | [has part](http://purl.obolibrary.org/obo/BFO_0000051) some ([metapleural sulcus](http://purl.obolibrary.org/obo/HAO_0000614) and ([bearer of](http://purl.obolibrary.org/obo/RO_0000053) some [horizontal](http://purl.obolibrary.org/obo/PATO_0001855))) | [has part](http://purl.obolibrary.org/obo/BFO_0000051) some ([metapleural sulcus](http://purl.obolibrary.org/obo/HAO_0000614) and ([is bearer of](http://purl.obolibrary.org/obo/BFO_0000053) some [horizontal](http://purl.obolibrary.org/obo/PATO_0001855))) |  |
| Metatibia length vs. metabasitarsus length: metatibia 1.4x to 1.6x as long as metabasitarsus | [has part](http://purl.obolibrary.org/obo/BFO_0000051) some ([metatibia](http://purl.obolibrary.org/obo/HAO_0000631) and ([has part](http://purl.obolibrary.org/obo/BFO_0000051) some ([proximodistal anatomical line](http://purl.obolibrary.org/obo/HAO_0002273) and ([bearer of](http://purl.obolibrary.org/obo/RO_0000053) some ([length](http://purl.obolibrary.org/obo/PATO_0000122) and ([is quality measured as](http://purl.obolibrary.org/obo/IAO_0000417) some (([has measurement unit label](http://purl.obolibrary.org/obo/IAO_0000039) some ([length](http://purl.obolibrary.org/obo/PATO_0000122) and ([inheres in](http://purl.obolibrary.org/obo/RO_0000052) some ([proximodistal anatomical line](http://purl.obolibrary.org/obo/HAO_0002273) and ([part of](http://purl.obolibrary.org/obo/BFO_0000050) some [metabasitarsus](http://purl.obolibrary.org/obo/HAO_0001142)))))) and ([has measurement value](http://purl.obolibrary.org/obo/IAO_0000004) some [float](http://www.w3.org/2001/XMLSchema#float)[>= 1.4f , <= 1.6f])))))))) |  | [has_part](http://purl.obolibrary.org/obo/BFO_0000051) some ([metatibia](http://purl.obolibrary.org/obo/HAO_0000631) and ([is bearer of](http://purl.obolibrary.org/obo/BFO_0000053) some ([length](http://purl.obolibrary.org/obo/PATO_0000122) and ([has_measurement](http://hymao.org/has_measurement) some ([has_magnitude](http://hymao.org/has_magnitude) some float[>= 1.4f , <= 1.6f])) and ([has_unit](http://hymao.org/has_unit) some ([length](http://purl.obolibrary.org/obo/PATO_0000122) and ([inheres in](http://purl.obolibrary.org/obo/BFO_0000052) some metabasitarsus)))))) |
| Metatibia length vs. metabasitarsus length: metatibia 2x as long as metabasitarsus | [has part](http://purl.obolibrary.org/obo/BFO_0000051) some ([metatibia](http://purl.obolibrary.org/obo/HAO_0000631) and ([has part](http://purl.obolibrary.org/obo/BFO_0000051) some ([proximodistal anatomical line](http://purl.obolibrary.org/obo/HAO_0002273) and ([bearer of](http://purl.obolibrary.org/obo/RO_0000053) some ([length](http://purl.obolibrary.org/obo/PATO_0000122) and ([is quality measured as](http://purl.obolibrary.org/obo/IAO_0000417) some (([has measurement unit label](http://purl.obolibrary.org/obo/IAO_0000039) some ([length](http://purl.obolibrary.org/obo/PATO_0000122) and ([inheres in](http://purl.obolibrary.org/obo/RO_0000052) some ([proximodistal anatomical line](http://purl.obolibrary.org/obo/HAO_0002273) and ([part of](http://purl.obolibrary.org/obo/BFO_0000050) some [metabasitarsus](http://purl.obolibrary.org/obo/HAO_0001142)))))) and ([has measurement value](http://purl.obolibrary.org/obo/IAO_0000004) value 2)))))))) |  | [has_part](http://purl.obolibrary.org/obo/BFO_0000051) some ([metatibia](http://purl.obolibrary.org/obo/HAO_0000631) and ([is bearer of](http://purl.obolibrary.org/obo/BFO_0000053) some ([length](http://purl.obolibrary.org/obo/PATO_0000122) and ([has_measurement](http://hymao.org/has_measurement) some (([has_unit](http://hymao.org/has_unit) some ([length](http://purl.obolibrary.org/obo/PATO_0000122) and ([inheres in](http://purl.obolibrary.org/obo/BFO_0000052) some metabasitarsus))) and ([has_magnitude](http://hymao.org/has_magnitude) value 2.0f)))))) |
| Metatibia length vs. metabasitarsus length: equal | [has part](http://purl.obolibrary.org/obo/BFO_0000051) some ([metatibia](http://purl.obolibrary.org/obo/HAO_0000631) and ([has part](http://purl.obolibrary.org/obo/BFO_0000051) some ([proximodistal anatomical line](http://purl.obolibrary.org/obo/HAO_0002273) and ([bearer of](http://purl.obolibrary.org/obo/RO_0000053) some ([length](http://purl.obolibrary.org/obo/PATO_0000122) and ([similar_in_magnitude_relative_to](http://purl.obolibrary.org/obo/pato#similar_in_magnitude_relative_to) some ([length](http://purl.obolibrary.org/obo/PATO_0000122) and ([inheres in](http://purl.obolibrary.org/obo/RO_0000052) some ([proximodistal anatomical line](http://purl.obolibrary.org/obo/HAO_0002273) and ([part of](http://purl.obolibrary.org/obo/BFO_0000050) some [metabasitarsus](http://purl.obolibrary.org/obo/HAO_0001142))))))))))) |  | [has_part](http://purl.obolibrary.org/obo/BFO_0000051) some ([metatibia](http://purl.obolibrary.org/obo/HAO_0000631) and ([is bearer of](http://purl.obolibrary.org/obo/BFO_0000053) some ([length](http://purl.obolibrary.org/obo/PATO_0000122) and ([similar_in_magnitude_relative_to](http://purl.obolibrary.org/obo/pato#similar_in_magnitude_relative_to) some ([length](http://purl.obolibrary.org/obo/PATO_0000122) and ([inheres in](http://purl.obolibrary.org/obo/BFO_0000052) some metabasitarsus)))))) |
| Metatibia length vs. metabasitarsus length: metatibia 1.2x to 1.4x as long as metabasitarsus | [has part](http://purl.obolibrary.org/obo/BFO_0000051) some ([metatibia](http://purl.obolibrary.org/obo/HAO_0000631) and ([has part](http://purl.obolibrary.org/obo/BFO_0000051) some ([proximodistal anatomical line](http://purl.obolibrary.org/obo/HAO_0002273) and ([bearer of](http://purl.obolibrary.org/obo/RO_0000053) some ([length](http://purl.obolibrary.org/obo/PATO_0000122) and ([is quality measured as](http://purl.obolibrary.org/obo/IAO_0000417) some (([has measurement unit label](http://purl.obolibrary.org/obo/IAO_0000039) some ([length](http://purl.obolibrary.org/obo/PATO_0000122) and ([inheres in](http://purl.obolibrary.org/obo/RO_0000052) some ([proximodistal anatomical line](http://purl.obolibrary.org/obo/HAO_0002273) and ([part of](http://purl.obolibrary.org/obo/BFO_0000050) some [metabasitarsus](http://purl.obolibrary.org/obo/HAO_0001142)))))) and ([has measurement value](http://purl.obolibrary.org/obo/IAO_0000004) some [float](http://www.w3.org/2001/XMLSchema#float)[>= 1.2f , <= 1.4f])))))))) |  | [has_part](http://purl.obolibrary.org/obo/BFO_0000051) some ([metatibia](http://purl.obolibrary.org/obo/HAO_0000631) and ([is bearer of](http://purl.obolibrary.org/obo/BFO_0000053) some ([length](http://purl.obolibrary.org/obo/PATO_0000122) and ([has_measurement](http://hymao.org/has_measurement) some ([has_magnitude](http://hymao.org/has_magnitude) some float[>= 1.2f , <= 1.4f])) and ([has_unit](http://hymao.org/has_unit) some ([length](http://purl.obolibrary.org/obo/PATO_0000122) and ([inheres in](http://purl.obolibrary.org/obo/BFO_0000052) some metabasitarsus)))))) |
| Notaulus count: present | [has part](http://purl.obolibrary.org/obo/BFO_0000051) some [notaulus](http://purl.obolibrary.org/obo/HAO_0000647) |  |  |
| Notaulus count: absent | not ([has part](http://purl.obolibrary.org/obo/BFO_0000051) some [notaulus](http://purl.obolibrary.org/obo/HAO_0000647)) |  |  |
| Notaulus shape: sigmoid | [has part](http://purl.obolibrary.org/obo/BFO_0000051) some ([notaulus](http://purl.obolibrary.org/obo/HAO_0000647) and ([bearer of](http://purl.obolibrary.org/obo/RO_0000053) some [sigmoid](http://purl.obolibrary.org/obo/PATO_0001878))) | [has part](http://purl.obolibrary.org/obo/BFO_0000051) some ([notaulus](http://purl.obolibrary.org/obo/HAO_0000647) and ([is bearer of](http://purl.obolibrary.org/obo/BFO_0000053) some [sigmoid](http://purl.obolibrary.org/obo/PATO_0001878))) |  |
| Notaulus shape: falciform | [has part](http://purl.obolibrary.org/obo/BFO_0000051) some ([notaulus](http://purl.obolibrary.org/obo/HAO_0000647) and ([bearer of](http://purl.obolibrary.org/obo/RO_0000053) some [falciform](http://purl.obolibrary.org/obo/PATO_0002215))) | [has part](http://purl.obolibrary.org/obo/BFO_0000051) some ([notaulus](http://purl.obolibrary.org/obo/HAO_0000647) and ([is bearer of](http://purl.obolibrary.org/obo/BFO_0000053) some [falciform](http://purl.obolibrary.org/obo/PATO_0002215))) |  |
| Nucha count: present | [has part](http://purl.obolibrary.org/obo/BFO_0000051) some [nucha](http://purl.obolibrary.org/obo/HAO_0000651) | [has part](http://purl.obolibrary.org/obo/BFO_0000051) some [nucha](http://purl.obolibrary.org/obo/HAO_0000651) |  |
| Nucha count: absent | not ([has part](http://purl.obolibrary.org/obo/BFO_0000051) some [nucha](http://purl.obolibrary.org/obo/HAO_0000651)) | not ([has part](http://purl.obolibrary.org/obo/BFO_0000051) some [nucha](http://purl.obolibrary.org/obo/HAO_0000651)) |  |
| Parapsidal signum count: present | [has part](http://purl.obolibrary.org/obo/BFO_0000051) some [parapsidal line](http://purl.obolibrary.org/obo/HAO_0000694) |  | [has_part](http://purl.obolibrary.org/obo/BFO_0000051) some [parapsidal line](http://purl.obolibrary.org/obo/HAO_0000694) |
| Parapsidal signum count: absent | not ([has part](http://purl.obolibrary.org/obo/BFO_0000051) some [parapsidal line](http://purl.obolibrary.org/obo/HAO_0000694)) |  | [has_part](http://purl.obolibrary.org/obo/BFO_0000051) only (not ([parapsidal line](http://purl.obolibrary.org/obo/HAO_0000694))) |
| Petiolar scrobe pubescence count: present | [has part](http://purl.obolibrary.org/obo/BFO_0000051) some ([petiolar scrobe](http://purl.obolibrary.org/obo/HAO_0002265) and ([has part](http://purl.obolibrary.org/obo/BFO_0000051) some [seta](http://purl.obolibrary.org/obo/HAO_0000935))) |  |  |
| Petiolar scrobe pubescence count: absent | [has part](http://purl.obolibrary.org/obo/BFO_0000051) some ([petiolar scrobe](http://purl.obolibrary.org/obo/HAO_0002265) and (not ([has part](http://purl.obolibrary.org/obo/BFO_0000051) some [seta](http://purl.obolibrary.org/obo/HAO_0000935)))) |  |  |
| Petiole color: anterior region yellowish posterior region brownish | [has part](http://purl.obolibrary.org/obo/BFO_0000051) some ([abdominal segment 2](http://purl.obolibrary.org/obo/HAO_0000020) and ([has part](http://purl.obolibrary.org/obo/BFO_0000051) some ([anterior region](http://purl.obolibrary.org/obo/BSPO_0000071) and ([bearer of](http://purl.obolibrary.org/obo/RO_0000053) some [yellow](http://purl.obolibrary.org/obo/PATO_0000324)))) and ([has part](http://purl.obolibrary.org/obo/BFO_0000051) some ((not ([anterior region](http://purl.obolibrary.org/obo/BSPO_0000071) and ([bearer of](http://purl.obolibrary.org/obo/RO_0000053) some [yellow](http://purl.obolibrary.org/obo/PATO_0000324)))) and ([bearer of](http://purl.obolibrary.org/obo/RO_0000053) some [brown](http://purl.obolibrary.org/obo/PATO_0000952))))) |  |  |
| Petiole color: black | [has part](http://purl.obolibrary.org/obo/BFO_0000051) some ([abdominal segment 2](http://purl.obolibrary.org/obo/HAO_0000020) and ([bearer of](http://purl.obolibrary.org/obo/RO_0000053) some [black](http://purl.obolibrary.org/obo/PATO_0000317))) |  |  |
| Petiole color: brown | [has part](http://purl.obolibrary.org/obo/BFO_0000051) some ([abdominal segment 2](http://purl.obolibrary.org/obo/HAO_0000020) and ([bearer of](http://purl.obolibrary.org/obo/RO_0000053) some [brown](http://purl.obolibrary.org/obo/PATO_0000952))) |  |  |
| Petiole pilosity: sparse | [has part](http://purl.obolibrary.org/obo/BFO_0000051) some ([abdominal segment 2](http://purl.obolibrary.org/obo/HAO_0000020) and ([bearer of](http://purl.obolibrary.org/obo/RO_0000053) some ([pilosity](http://purl.obolibrary.org/obo/HAO_0001990) and (not ([hairy](http://purl.obolibrary.org/obo/PATO_0000454)))))) | [has part](http://purl.obolibrary.org/obo/BFO_0000051) some ([abdominal segment 2](http://purl.obolibrary.org/obo/HAO_0000020) and ([is bearer of](http://purl.obolibrary.org/obo/BFO_0000053) some ([pilosity](http://purl.obolibrary.org/obo/PATO_0000066) and (not ([hairy](http://purl.obolibrary.org/obo/PATO_0000454)))))) |  |
| Petiole pilosity: dense | [has part](http://purl.obolibrary.org/obo/BFO_0000051) some ([abdominal segment 2](http://purl.obolibrary.org/obo/HAO_0000020) and ([bearer of](http://purl.obolibrary.org/obo/RO_0000053) some [setose](http://purl.obolibrary.org/obo/PATO_0002289))) | [has part](http://purl.obolibrary.org/obo/BFO_0000051) some ([abdominal segment 2](http://purl.obolibrary.org/obo/HAO_0000020) and ([is bearer of](http://purl.obolibrary.org/obo/BFO_0000053) some [setose](http://purl.obolibrary.org/obo/PATO_0002289))) |  |
| Petiole texture: furrowed | [has part](http://purl.obolibrary.org/obo/BFO_0000051) some ([abdominal segment 2](http://purl.obolibrary.org/obo/HAO_0000020) and (([has part](http://purl.obolibrary.org/obo/BFO_0000051) some [dorsal side](http://purl.obolibrary.org/obo/BSPO_0000063)) and ([bearer of](http://purl.obolibrary.org/obo/RO_0000053) some [furrowed](http://purl.obolibrary.org/obo/PATO_0001948)))) | [has part](http://purl.obolibrary.org/obo/BFO_0000051) some ([abdominal segment 2](http://purl.obolibrary.org/obo/HAO_0000020) and ([is bearer of](http://purl.obolibrary.org/obo/BFO_0000053) some [furrowed](http://purl.obolibrary.org/obo/PATO_0001948))) |  |
| Petiole texture: foveolate | [has part](http://purl.obolibrary.org/obo/BFO_0000051) some ([abdominal segment 2](http://purl.obolibrary.org/obo/HAO_0000020) and (([has part](http://purl.obolibrary.org/obo/BFO_0000051) some [dorsal side](http://purl.obolibrary.org/obo/BSPO_0000063)) and ([bearer of](http://purl.obolibrary.org/obo/RO_0000053) some [foveate](http://purl.obolibrary.org/obo/PATO_0002296)))) |  |  |
| Petiole texture: smooth | [has part](http://purl.obolibrary.org/obo/BFO_0000051) some ([abdominal segment 2](http://purl.obolibrary.org/obo/HAO_0000020) and (([has part](http://purl.obolibrary.org/obo/BFO_0000051) some [dorsal side](http://purl.obolibrary.org/obo/BSPO_0000063)) and ([bearer of](http://purl.obolibrary.org/obo/RO_0000053) some [smooth](http://purl.obolibrary.org/obo/PATO_0000701)))) | [has part](http://purl.obolibrary.org/obo/BFO_0000051) some ([abdominal segment 2](http://purl.obolibrary.org/obo/HAO_0000020) and ([is bearer of](http://purl.obolibrary.org/obo/BFO_0000053) some [smooth](http://purl.obolibrary.org/obo/PATO_0000701))) |  |
| Posterior tooth of the mandible count: absent | not ([has part](http://purl.obolibrary.org/obo/BFO_0000051) some [posterior tooth of the mandible](http://purl.obolibrary.org/obo/HAO_0002252)) |  |  |
| Posterior tooth of the mandible count: present | [has part](http://purl.obolibrary.org/obo/BFO_0000051) some [posterior tooth of the mandible](http://purl.obolibrary.org/obo/HAO_0002252) |  |  |
| Posterodistal notch of the fore wing count: present | [has part](http://purl.obolibrary.org/obo/BFO_0000051) some [posterodistal notch of the fore wing](http://purl.obolibrary.org/obo/HAO_0002261) |  |  |
| Posterodistal notch of the fore wing count: absent | not ([has part](http://purl.obolibrary.org/obo/BFO_0000051) some [posterodistal notch of the fore wing](http://purl.obolibrary.org/obo/HAO_0002261)) |  |  |
| Poststigmal fold line count: present | [has part](http://purl.obolibrary.org/obo/BFO_0000051) some [wing crease](http://purl.obolibrary.org/obo/HAO_0001092) |  |  |
| Poststigmal fold line count: absent | not ([phenotype_23441](http://hymao.org/miko2014/phenotypes.owl#phenotype_23441)) |  |  |
| Preorbital carina count: present | [has part](http://purl.obolibrary.org/obo/BFO_0000051) some [preorbital carina](http://purl.obolibrary.org/obo/HAO_0000810) |  |  |
| Preorbital carina count: absent | not ([has part](http://purl.obolibrary.org/obo/BFO_0000051) some [preorbital carina](http://purl.obolibrary.org/obo/HAO_0000810)) |  |  |
| Prestigmal flexion line count: absent | not ([phenotype_23435](http://hymao.org/miko2014/phenotypes.owl#phenotype_23435)) |  |  |
| Prestigmal flexion line count: present | [has part](http://purl.obolibrary.org/obo/BFO_0000051) some [wing crease](http://purl.obolibrary.org/obo/HAO_0001092) |  |  |
| Profemoral scrobe of the mesopectus count: absent | not ([has part](http://purl.obolibrary.org/obo/BFO_0000051) some [profemoral scrobe of the mesopectus](http://purl.obolibrary.org/obo/HAO_0002258)) |  |  |
| Profemoral scrobe of the mesopectus count: present | [has part](http://purl.obolibrary.org/obo/BFO_0000051) some [profemoral scrobe of the mesopectus](http://purl.obolibrary.org/obo/HAO_0002258) |  |  |
| Pronotal lobe carina count: absent | [has part](http://purl.obolibrary.org/obo/BFO_0000051) some ([pronotal lobe](http://purl.obolibrary.org/obo/HAO_0000836) and (not ([has part](http://purl.obolibrary.org/obo/BFO_0000051) some [carina](http://purl.obolibrary.org/obo/HAO_0000188)))) | [has part](http://purl.obolibrary.org/obo/BFO_0000051) some ([pronotal lobe](http://purl.obolibrary.org/obo/HAO_0000836) and (not ([has part](http://purl.obolibrary.org/obo/BFO_0000051) some [carina](http://purl.obolibrary.org/obo/HAO_0000188)))) |  |
| Pronotal lobe carina count: present | [has part](http://purl.obolibrary.org/obo/BFO_0000051) some ([pronotal lobe](http://purl.obolibrary.org/obo/HAO_0000836) and ([has part](http://purl.obolibrary.org/obo/BFO_0000051) some [carina](http://purl.obolibrary.org/obo/HAO_0000188))) | [has part](http://purl.obolibrary.org/obo/BFO_0000051) some ([pronotal lobe](http://purl.obolibrary.org/obo/HAO_0000836) and ([has part](http://purl.obolibrary.org/obo/BFO_0000051) some [carina](http://purl.obolibrary.org/obo/HAO_0000188))) |  |
| Proximodistal length of pedicel vs. proximodistal length of first flagellomere in male: pedicel at least as long as first flagellomere | [has part](http://purl.obolibrary.org/obo/BFO_0000051) some ([pedicel](http://purl.obolibrary.org/obo/HAO_0000706) and ([has part](http://purl.obolibrary.org/obo/BFO_0000051) some ([proximodistal anatomical line](http://purl.obolibrary.org/obo/HAO_0002273) and ([bearer of](http://purl.obolibrary.org/obo/RO_0000053) some ([length](http://purl.obolibrary.org/obo/PATO_0000122) and ([is quality measured as](http://purl.obolibrary.org/obo/IAO_0000417) some (([has measurement unit label](http://purl.obolibrary.org/obo/IAO_0000039) some ([length](http://purl.obolibrary.org/obo/PATO_0000122) and ([inheres in](http://purl.obolibrary.org/obo/RO_0000052) some ([proximodistal anatomical line](http://purl.obolibrary.org/obo/HAO_0002273) and ([part of](http://purl.obolibrary.org/obo/BFO_0000050) some [first flagellomere](http://purl.obolibrary.org/obo/HAO_0001148)))))) and ([has measurement value](http://purl.obolibrary.org/obo/IAO_0000004) some [float](http://www.w3.org/2001/XMLSchema#float)[>= 1])))))))) |  |  |
| : Proximodistal length of pedicel vs. proximodistal length of first flagellomere in male: pedicel distinctly shorter than first flagellomere | [has part](http://purl.obolibrary.org/obo/BFO_0000051) some ([pedicel](http://purl.obolibrary.org/obo/HAO_0000706) and ([has part](http://purl.obolibrary.org/obo/BFO_0000051) some ([proximodistal anatomical line](http://purl.obolibrary.org/obo/HAO_0002273) and ([bearer of](http://purl.obolibrary.org/obo/RO_0000053) some ([length](http://purl.obolibrary.org/obo/PATO_0000122) and ([decreased_in_magnitude_relative_to](http://purl.obolibrary.org/obo/pato#decreased_in_magnitude_relative_to) some ([length](http://purl.obolibrary.org/obo/PATO_0000122) and ([inheres in](http://purl.obolibrary.org/obo/RO_0000052) some ([proximodistal anatomical line](http://purl.obolibrary.org/obo/HAO_0002273) and ([part of](http://purl.obolibrary.org/obo/BFO_0000050) some [first flagellomere](http://purl.obolibrary.org/obo/HAO_0001148)))))))) and ([has measurement value](http://purl.obolibrary.org/obo/IAO_0000004) some [float](http://www.w3.org/2001/XMLSchema#float)[>= 1])))) |  |  |
| Scrobal carina of the anteromesoscutum: absent | not ([has part](http://purl.obolibrary.org/obo/BFO_0000051) some [scrobal carina of the mesoscutum](http://purl.obolibrary.org/obo/HAO_0002256)) |  |  |
| Scrobal carina of the anteromesoscutum: present | [has part](http://purl.obolibrary.org/obo/BFO_0000051) some [scrobal carina of the mesoscutum](http://purl.obolibrary.org/obo/HAO_0002256) |  |  |
| Scutoscutellar suture sculpture: not foveate | [has part](http://purl.obolibrary.org/obo/BFO_0000051) some ([scutoscutellar suture](http://purl.obolibrary.org/obo/HAO_0000920) and (not ([has part](http://purl.obolibrary.org/obo/BFO_0000051) some [depression](http://purl.obolibrary.org/obo/HAO_0000241)))) | [has part](http://purl.obolibrary.org/obo/BFO_0000051) some ([scutoscutellar suture](http://purl.obolibrary.org/obo/HAO_0000920) and ([is bearer of](http://purl.obolibrary.org/obo/BFO_0000053) some ([texture](http://purl.obolibrary.org/obo/PATO_0000150) and (not ([foveate](http://purl.obolibrary.org/obo/PATO_0002296)))))) |  |
| Scutoscutellar suture sculpture: foveate | [has part](http://purl.obolibrary.org/obo/BFO_0000051) some ([scutoscutellar suture](http://purl.obolibrary.org/obo/HAO_0000920) and ([has part](http://purl.obolibrary.org/obo/BFO_0000051) some [depression](http://purl.obolibrary.org/obo/HAO_0000241))) | [has part](http://purl.obolibrary.org/obo/BFO_0000051) some ([scutoscutellar suture](http://purl.obolibrary.org/obo/HAO_0000920) and ([is bearer of](http://purl.obolibrary.org/obo/BFO_0000053) some [foveate](http://purl.obolibrary.org/obo/PATO_0002296))) |  |
| Setiferous patch on dorsal region of abdominal terga 4-7 in female count: present | ([has part](http://purl.obolibrary.org/obo/BFO_0000051) some ([abdominal tergum 4](http://purl.obolibrary.org/obo/HAO_0000057) and ([has part](http://purl.obolibrary.org/obo/BFO_0000051) some ([dorsal region](http://purl.obolibrary.org/obo/BSPO_0000079) and ([has part](http://purl.obolibrary.org/obo/BFO_0000051) some [setiferous patch](http://purl.obolibrary.org/obo/HAO_0000936)))))) and ([has part](http://purl.obolibrary.org/obo/BFO_0000051) some ([abdominal tergum 5](http://purl.obolibrary.org/obo/HAO_0000058) and ([has part](http://purl.obolibrary.org/obo/BFO_0000051) some ([dorsal region](http://purl.obolibrary.org/obo/BSPO_0000079) and ([has part](http://purl.obolibrary.org/obo/BFO_0000051) some [setiferous patch](http://purl.obolibrary.org/obo/HAO_0000936)))))) and ([has part](http://purl.obolibrary.org/obo/BFO_0000051) some ([abdominal tergum 6](http://purl.obolibrary.org/obo/HAO_0000059) and ([has part](http://purl.obolibrary.org/obo/BFO_0000051) some ([dorsal region](http://purl.obolibrary.org/obo/BSPO_0000079) and ([has part](http://purl.obolibrary.org/obo/BFO_0000051) some [setiferous patch](http://purl.obolibrary.org/obo/HAO_0000936)))))) and ([has part](http://purl.obolibrary.org/obo/BFO_0000051) some ([abdominal tergum 7](http://purl.obolibrary.org/obo/HAO_0000060) and ([has part](http://purl.obolibrary.org/obo/BFO_0000051) some ([dorsal region](http://purl.obolibrary.org/obo/BSPO_0000079) and ([has part](http://purl.obolibrary.org/obo/BFO_0000051) some [setiferous patch](http://purl.obolibrary.org/obo/HAO_0000936)))))) | ([has part](http://purl.obolibrary.org/obo/BFO_0000051) some ([abdominal tergum 4](http://purl.obolibrary.org/obo/HAO_0000057) and ([has part](http://purl.obolibrary.org/obo/BFO_0000051) some ([dorsal region](http://purl.obolibrary.org/obo/BSPO_0000079) and ([has part](http://purl.obolibrary.org/obo/BFO_0000051) some [setiferous patch](http://purl.obolibrary.org/obo/HAO_0000936)))))) and ([has part](http://purl.obolibrary.org/obo/BFO_0000051) some ([abdominal tergum 5](http://purl.obolibrary.org/obo/HAO_0000058) and ([has part](http://purl.obolibrary.org/obo/BFO_0000051) some ([dorsal region](http://purl.obolibrary.org/obo/BSPO_0000079) and ([has part](http://purl.obolibrary.org/obo/BFO_0000051) some [setiferous patch](http://purl.obolibrary.org/obo/HAO_0000936)))))) and ([has part](http://purl.obolibrary.org/obo/BFO_0000051) some ([abdominal tergum 6](http://purl.obolibrary.org/obo/HAO_0000059) and ([has part](http://purl.obolibrary.org/obo/BFO_0000051) some ([dorsal region](http://purl.obolibrary.org/obo/BSPO_0000079) and ([has part](http://purl.obolibrary.org/obo/BFO_0000051) some [setiferous patch](http://purl.obolibrary.org/obo/HAO_0000936)))))) and ([has part](http://purl.obolibrary.org/obo/BFO_0000051) some ([abdominal tergum 7](http://purl.obolibrary.org/obo/HAO_0000060) and ([has part](http://purl.obolibrary.org/obo/BFO_0000051) some ([dorsal region](http://purl.obolibrary.org/obo/BSPO_0000079) and ([has part](http://purl.obolibrary.org/obo/BFO_0000051) some [setiferous patch](http://purl.obolibrary.org/obo/HAO_0000936)))))) |  |
| Setiferous patch on dorsal region of abdominal terga 4-7 in female count: absent | ([has part](http://purl.obolibrary.org/obo/BFO_0000051) some ([abdominal tergum 4](http://purl.obolibrary.org/obo/HAO_0000057) and ([has part](http://purl.obolibrary.org/obo/BFO_0000051) some ([dorsal region](http://purl.obolibrary.org/obo/BSPO_0000079) and (not ([has part](http://purl.obolibrary.org/obo/BFO_0000051) some [setiferous patch](http://purl.obolibrary.org/obo/HAO_0000936))))))) and ([has part](http://purl.obolibrary.org/obo/BFO_0000051) some ([abdominal tergum 5](http://purl.obolibrary.org/obo/HAO_0000058) and ([has part](http://purl.obolibrary.org/obo/BFO_0000051) some ([dorsal region](http://purl.obolibrary.org/obo/BSPO_0000079) and (not ([has part](http://purl.obolibrary.org/obo/BFO_0000051) some [setiferous patch](http://purl.obolibrary.org/obo/HAO_0000936))))))) and ([has part](http://purl.obolibrary.org/obo/BFO_0000051) some ([abdominal tergum 6](http://purl.obolibrary.org/obo/HAO_0000059) and ([has part](http://purl.obolibrary.org/obo/BFO_0000051) some ([dorsal region](http://purl.obolibrary.org/obo/BSPO_0000079) and (not ([has part](http://purl.obolibrary.org/obo/BFO_0000051) some [setiferous patch](http://purl.obolibrary.org/obo/HAO_0000936))))))) and ([has part](http://purl.obolibrary.org/obo/BFO_0000051) some ([abdominal tergum 7](http://purl.obolibrary.org/obo/HAO_0000060) and ([has part](http://purl.obolibrary.org/obo/BFO_0000051) some ([dorsal region](http://purl.obolibrary.org/obo/BSPO_0000079) and (not ([has part](http://purl.obolibrary.org/obo/BFO_0000051) some [setiferous patch](http://purl.obolibrary.org/obo/HAO_0000936))))))) | ([has part](http://purl.obolibrary.org/obo/BFO_0000051) some ([abdominal tergum 4](http://purl.obolibrary.org/obo/HAO_0000057) and ([has part](http://purl.obolibrary.org/obo/BFO_0000051) some ([dorsal region](http://purl.obolibrary.org/obo/BSPO_0000079) and (not ([has part](http://purl.obolibrary.org/obo/BFO_0000051) some [setiferous patch](http://purl.obolibrary.org/obo/HAO_0000936))))))) and ([has part](http://purl.obolibrary.org/obo/BFO_0000051) some ([abdominal tergum 5](http://purl.obolibrary.org/obo/HAO_0000058) and ([has part](http://purl.obolibrary.org/obo/BFO_0000051)some ([dorsal region](http://purl.obolibrary.org/obo/BSPO_0000079) and (not ([has part](http://purl.obolibrary.org/obo/BFO_0000051) some [setiferous patch](http://purl.obolibrary.org/obo/HAO_0000936))))))) and ([has part](http://purl.obolibrary.org/obo/BFO_0000051) some ([abdominal tergum 6](http://purl.obolibrary.org/obo/HAO_0000059) and ([has part](http://purl.obolibrary.org/obo/BFO_0000051) some ([dorsal region](http://purl.obolibrary.org/obo/BSPO_0000079) and (not ([has part](http://purl.obolibrary.org/obo/BFO_0000051) some [setiferous patch](http://purl.obolibrary.org/obo/HAO_0000936))))))) and ([has part](http://purl.obolibrary.org/obo/BFO_0000051) some ([abdominal tergum 7](http://purl.obolibrary.org/obo/HAO_0000060) and ([has part](http://purl.obolibrary.org/obo/BFO_0000051) some ([dorsal region](http://purl.obolibrary.org/obo/BSPO_0000079) and (not ([has part](http://purl.obolibrary.org/obo/BFO_0000051) some [setiferous patch](http://purl.obolibrary.org/obo/HAO_0000936))))))) |  |
| Speculum count: absent | not ([has part](http://purl.obolibrary.org/obo/BFO_0000051) some [speculum](http://purl.obolibrary.org/obo/HAO_0000944)) | not ([has part](http://purl.obolibrary.org/obo/BFO_0000051) some [speculum](http://purl.obolibrary.org/obo/HAO_0000944)) |  |
| Speculum count: present | [has part](http://purl.obolibrary.org/obo/BFO_0000051) some [speculum](http://purl.obolibrary.org/obo/HAO_0000944) | [has part](http://purl.obolibrary.org/obo/BFO_0000051) some [speculum](http://purl.obolibrary.org/obo/HAO_0001667) |  |
| Subantennal carina count: present | [has part](http://purl.obolibrary.org/obo/BFO_0000051) some [subantennal carina](http://purl.obolibrary.org/obo/HAO_0001971) |  |  |
| Subantennal carina count: absent | not ([has part](http://purl.obolibrary.org/obo/BFO_0000051) some [subantennal carina](http://purl.obolibrary.org/obo/HAO_0001971)) |  |  |
| Subantennal groove count: present | [has part](http://purl.obolibrary.org/obo/BFO_0000051) some [subantennal groove](http://purl.obolibrary.org/obo/HAO_0000965) |  |  |
| Subantennal groove count: absent | not ([has part](http://purl.obolibrary.org/obo/BFO_0000051) some [subantennal groove](http://purl.obolibrary.org/obo/HAO_0000965)) |  |  |
| Submedian propodeal projection count: absent | not ([has part](http://purl.obolibrary.org/obo/BFO_0000051) some [anterior propodeal projection](http://purl.obolibrary.org/obo/HAO_0001968)) | [has part](http://purl.obolibrary.org/obo/BFO_0000051) some ([propodeum](http://purl.obolibrary.org/obo/HAO_0001248) and ([has part](http://purl.obolibrary.org/obo/BFO_0000051) some ([dorso-lateral region](http://purl.obolibrary.org/obo/BSPO_0000080) and (not ([has part](http://purl.obolibrary.org/obo/BFO_0000051) some [projection](http://purl.obolibrary.org/obo/HAO_0000829)))))) |  |
| Submedian propodeal projection count: present | [has part](http://purl.obolibrary.org/obo/BFO_0000051) some [anterior propodeal projection](http://purl.obolibrary.org/obo/HAO_0001968) | [has part](http://purl.obolibrary.org/obo/BFO_0000051) some ([propodeum](http://purl.obolibrary.org/obo/HAO_0001248) and ([has part](http://purl.obolibrary.org/obo/BFO_0000051) some ([dorso-lateral region](http://purl.obolibrary.org/obo/BSPO_0000080) and ([has part](http://purl.obolibrary.org/obo/BFO_0000051) some [projection](http://purl.obolibrary.org/obo/HAO_0000829))))) |  |
| Transmetapectal line count: absent | not ([has part](http://purl.obolibrary.org/obo/BFO_0000051) some [transmetapectal carina](http://purl.obolibrary.org/obo/HAO_0001887)) |  |  |
| Transmetapectal line count: present | [has part](http://purl.obolibrary.org/obo/BFO_0000051) some [transmetapectal carina](http://purl.obolibrary.org/obo/HAO_0001887) |  |  |
| Upper face sculpture: smooth | [has part](http://purl.obolibrary.org/obo/BFO_0000051) some ([upper face](http://purl.obolibrary.org/obo/HAO_0001044) and ([bearer of](http://purl.obolibrary.org/obo/RO_0000053) some [smooth](http://purl.obolibrary.org/obo/PATO_0000701))) |  | [has_part](http://purl.obolibrary.org/obo/BFO_0000051) some ([upper face](http://purl.obolibrary.org/obo/HAO_0001044) and ([is bearer of](http://purl.obolibrary.org/obo/BFO_0000053) some [smooth](http://purl.obolibrary.org/obo/PATO_0000701))) |
| Upper face sculpture: foveate | [has part](http://purl.obolibrary.org/obo/BFO_0000051) some ([upper face](http://purl.obolibrary.org/obo/HAO_0001044) and ([bearer of](http://purl.obolibrary.org/obo/RO_0000053) some [foveate](http://purl.obolibrary.org/obo/PATO_0002296))) |  | [has_part](http://purl.obolibrary.org/obo/BFO_0000051) some ([upper face](http://purl.obolibrary.org/obo/HAO_0001044) and ([is bearer of](http://purl.obolibrary.org/obo/BFO_0000053) some [punctate](http://purl.obolibrary.org/obo/PATO_0001512)) and ([is bearer of](http://purl.obolibrary.org/obo/BFO_0000053) some [foveate](http://purl.obolibrary.org/obo/PATO_0002296))) |
| Upper face sculpture: punctate and foveate | [has part](http://purl.obolibrary.org/obo/BFO_0000051) some ([upper face](http://purl.obolibrary.org/obo/HAO_0001044) and ([has part](http://purl.obolibrary.org/obo/BFO_0000051) some ([region](http://purl.obolibrary.org/obo/HAO_0000893) and ([has part](http://purl.obolibrary.org/obo/BFO_0000051) some ((not ([depression](http://purl.obolibrary.org/obo/HAO_0000241))) and ([bearer of](http://purl.obolibrary.org/obo/RO_0000053) some [punctate](http://purl.obolibrary.org/obo/PATO_0001512)))) and ([bearer of](http://purl.obolibrary.org/obo/RO_0000053) some [foveate](http://purl.obolibrary.org/obo/PATO_0002296))))) |  | [has_part](http://purl.obolibrary.org/obo/BFO_0000051) some ([upper face](http://purl.obolibrary.org/obo/HAO_0001044) and ([is bearer of](http://purl.obolibrary.org/obo/BFO_0000053) some [punctate](http://purl.obolibrary.org/obo/PATO_0001512)) and ([is bearer of](http://purl.obolibrary.org/obo/BFO_0000053) some [foveate](http://purl.obolibrary.org/obo/PATO_0002296))) |
| Ventral margin of mesopectus length: longer than ventral margin of metapectus length | [has part](http://purl.obolibrary.org/obo/BFO_0000051) some ([mesopectus](http://purl.obolibrary.org/obo/HAO_0000557) and ([has part](http://purl.obolibrary.org/obo/BFO_0000051) some ([lateral side](http://purl.obolibrary.org/obo/BSPO_0000066) and ([has part](http://purl.obolibrary.org/obo/BFO_0000051) some ([ventral margin](http://purl.obolibrary.org/obo/BSPO_0000684) and ([bearer of](http://purl.obolibrary.org/obo/RO_0000053) some ([length](http://purl.obolibrary.org/obo/PATO_0000122) and ([increased_in_magnitude_relative_to](http://purl.obolibrary.org/obo/pato#increased_in_magnitude_relative_to) some ([length](http://purl.obolibrary.org/obo/PATO_0000122) and ([inheres in](http://purl.obolibrary.org/obo/RO_0000052) some ([ventral margin](http://purl.obolibrary.org/obo/BSPO_0000684) and ([part of](http://purl.obolibrary.org/obo/BFO_0000050) some [lateral side](http://purl.obolibrary.org/obo/BSPO_0000066)) and ([part of](http://purl.obolibrary.org/obo/BFO_0000050) some [metapectus](http://purl.obolibrary.org/obo/HAO_0000605))))))))))))) | [has part](http://purl.obolibrary.org/obo/BFO_0000051) some ([mesopectus](http://purl.obolibrary.org/obo/HAO_0000557) and ([has part](http://purl.obolibrary.org/obo/BFO_0000051) some ([ventral margin](http://purl.obolibrary.org/obo/BSPO_0000684) and ([is bearer of](http://purl.obolibrary.org/obo/BFO_0000053) some ([length](http://purl.obolibrary.org/obo/PATO_0000122) and ([increased_in_magnitude_relative_to](http://purl.obolibrary.org/obo/pato#increased_in_magnitude_relative_to) some ([length](http://purl.obolibrary.org/obo/PATO_0000122) and ([inheres in](http://purl.obolibrary.org/obo/BFO_0000052) some ([ventral margin](http://purl.obolibrary.org/obo/BSPO_0000684) and ([part of](http://purl.obolibrary.org/obo/BFO_0000050) some [metapectus](http://purl.obolibrary.org/obo/HAO_0000605))))))))))) |  |
| Ventral margin of mesopectus length: shorter than ventral margin of metapectus length | [has part](http://purl.obolibrary.org/obo/BFO_0000051) some ([mesopectus](http://purl.obolibrary.org/obo/HAO_0000557) and ([has part](http://purl.obolibrary.org/obo/BFO_0000051) some ([lateral side](http://purl.obolibrary.org/obo/BSPO_0000066) and ([has part](http://purl.obolibrary.org/obo/BFO_0000051) some ([ventral margin](http://purl.obolibrary.org/obo/BSPO_0000684) and ([bearer of](http://purl.obolibrary.org/obo/RO_0000053) some ([length](http://purl.obolibrary.org/obo/PATO_0000122) and ([decreased_in_magnitude_relative_to](http://purl.obolibrary.org/obo/pato#decreased_in_magnitude_relative_to) some ([length](http://purl.obolibrary.org/obo/PATO_0000122) and ([inheres in](http://purl.obolibrary.org/obo/RO_0000052) some ([ventral margin](http://purl.obolibrary.org/obo/BSPO_0000684) and ([part of](http://purl.obolibrary.org/obo/BFO_0000050) some [lateral side](http://purl.obolibrary.org/obo/BSPO_0000066)) and ([part of](http://purl.obolibrary.org/obo/BFO_0000050) some [metapectus](http://purl.obolibrary.org/obo/HAO_0000605))))))))))))) | [has part](http://purl.obolibrary.org/obo/BFO_0000051) some ([mesopectus](http://purl.obolibrary.org/obo/HAO_0000557) and ([has part](http://purl.obolibrary.org/obo/BFO_0000051) some ([ventral margin](http://purl.obolibrary.org/obo/BSPO_0000684) and ([is bearer of](http://purl.obolibrary.org/obo/BFO_0000053) some ([length](http://purl.obolibrary.org/obo/PATO_0000122) and ([decreased_in_magnitude_relative_to](http://purl.obolibrary.org/obo/pato#decreased_in_magnitude_relative_to) some ([length](http://purl.obolibrary.org/obo/PATO_0000122) and ([inheres in](http://purl.obolibrary.org/obo/BFO_0000052) some ([ventral margin](http://purl.obolibrary.org/obo/BSPO_0000684) and ([part of](http://purl.obolibrary.org/obo/BFO_0000050) some [metapectus](http://purl.obolibrary.org/obo/HAO_0000605))))))))))) |  |
| Ventro-lateral region of mesosoma texture: foveate | [has part](http://purl.obolibrary.org/obo/BFO_0000051) some ([mesosoma](http://purl.obolibrary.org/obo/HAO_0000576) and ([has part](http://purl.obolibrary.org/obo/BFO_0000051) some ([ventro-lateral region](http://purl.obolibrary.org/obo/BSPO_0000085) and ([bearer of](http://purl.obolibrary.org/obo/RO_0000053) some [foveate](http://purl.obolibrary.org/obo/PATO_0002296))))) | [has part](http://purl.obolibrary.org/obo/BFO_0000051) some ([mesosoma](http://purl.obolibrary.org/obo/HAO_0000576) and ([has part](http://purl.obolibrary.org/obo/BFO_0000051) some ([ventro-lateral region](http://purl.obolibrary.org/obo/BSPO_0000085) and ([is bearer of](http://purl.obolibrary.org/obo/BFO_0000053) some [foveate](http://purl.obolibrary.org/obo/PATO_0002296))))) |  |
| Ventro-lateral region of mesosoma texture: areolate | [has part](http://purl.obolibrary.org/obo/BFO_0000051) some ([mesosoma](http://purl.obolibrary.org/obo/HAO_0000576) and ([has part](http://purl.obolibrary.org/obo/BFO_0000051) some ([ventro-lateral region](http://purl.obolibrary.org/obo/BSPO_0000085) and ([bearer of](http://purl.obolibrary.org/obo/RO_0000053) some [areolate](http://purl.obolibrary.org/obo/PATO_0002295))))) | [has part](http://purl.obolibrary.org/obo/BFO_0000051) some ([mesosoma](http://purl.obolibrary.org/obo/HAO_0000576) and ([has part](http://purl.obolibrary.org/obo/BFO_0000051) some ([ventro-lateral region](http://purl.obolibrary.org/obo/BSPO_0000085) and ([is bearer of](http://purl.obolibrary.org/obo/BFO_0000053) some [areolate](http://purl.obolibrary.org/obo/PATO_0002295))))) |  |
| Vertex sculpture: foveate | [has part](http://purl.obolibrary.org/obo/BFO_0000051) some ([vertex](http://purl.obolibrary.org/obo/HAO_0001077) and ([bearer of](http://purl.obolibrary.org/obo/RO_0000053) some [foveate](http://purl.obolibrary.org/obo/PATO_0002296))) |  | [has_part](http://purl.obolibrary.org/obo/BFO_0000051) some ([vertex](http://purl.obolibrary.org/obo/HAO_0001077) and ([is bearer of](http://purl.obolibrary.org/obo/BFO_0000053) some [foveate](http://purl.obolibrary.org/obo/PATO_0002296))) |
| Vertex sculpture: smooth | [has part](http://purl.obolibrary.org/obo/BFO_0000051) some ([vertex](http://purl.obolibrary.org/obo/HAO_0001077) and ([bearer of](http://purl.obolibrary.org/obo/RO_0000053) some [smooth](http://purl.obolibrary.org/obo/PATO_0000701))) |  | [has_part](http://purl.obolibrary.org/obo/BFO_0000051) some ([vertex](http://purl.obolibrary.org/obo/HAO_0001077) and ([is bearer of](http://purl.obolibrary.org/obo/BFO_0000053) some [smooth](http://purl.obolibrary.org/obo/PATO_0000701))) |
